# Engineering the protein dynamics of an ancestral luciferase

**DOI:** 10.1038/s41467-021-23450-z

**Published:** 2021-06-14

**Authors:** Andrea Schenkmayerova, Gaspar P. Pinto, Martin Toul, Martin Marek, Lenka Hernychova, Joan Planas-Iglesias, Veronika Daniel Liskova, Daniel Pluskal, Michal Vasina, Stephane Emond, Mark Dörr, Radka Chaloupkova, David Bednar, Zbynek Prokop, Florian Hollfelder, Uwe T. Bornscheuer, Jiri Damborsky

**Affiliations:** 1 International Clinical Research Center, St. Anne’s University Hospital Brno, Brno, Czech Republic; 2 Loschmidt Laboratories, Department of Experimental Biology and RECETOX, Faculty of Science, Masaryk University, Brno, Czech Republic; 3 Research Centre for Applied Molecular Oncology, Masaryk Memorial Cancer Institute, Brno, Czech Republic; 4 Department of Biochemistry, University of Cambridge, Cambridge, UK; 5 Department of Biotechnology and Enzyme Catalysis, Institute of Biochemistry, University of Greifswald, Greifswald, Germany

**Keywords:** Enzymes, Hydrolases, Protein design, Protein design, X-ray crystallography

## Abstract

Protein dynamics are often invoked in explanations of enzyme catalysis, but their design has proven elusive. Here we track the role of dynamics in evolution, starting from the evolvable and thermostable ancestral protein Anc^HLD-RLuc^ which catalyses both dehalogenase and luciferase reactions. Insertion-deletion (InDel) backbone mutagenesis of Anc^HLD-RLuc^ challenged the scaffold dynamics. Screening for both activities reveals InDel mutations localized in three distinct regions that lead to altered protein dynamics (based on crystallographic B-factors, hydrogen exchange, and molecular dynamics simulations). An anisotropic network model highlights the importance of the conformational flexibility of a loop-helix fragment of *Renilla* luciferases for ligand binding. Transplantation of this dynamic fragment leads to lower product inhibition and highly stable glow-type bioluminescence. The success of our approach suggests that a strategy comprising (i) constructing a stable and evolvable template, (ii) mapping functional regions by backbone mutagenesis, and (iii) transplantation of dynamic features, can lead to functionally innovative proteins.

## Abstract (web summary)

Directed evolution commonly relies on point mutations but InDels frequently occur in evolution. Here the authors report a protein-engineering framework based on InDel mutagenesis and fragment transplantation resulting in greater catalysis and longer glow-type bioluminescence of the ancestral luciferase.

## Introduction

Contemporary biocatalysts may originate from a small number of possibly multifunctional common ancestors and a limited number of structural folds. Natural enzymes have undergone billions of years of evolution to generate vast functional diversity and strikingly precise and efficient activities. This diversity resulted from complex evolutionary processes^1^, which have been grouped into two complementary mechanisms: creeping and leaping evolution^2^. The former involves relatively minor functional changes that generally increase specificity or activity, whereas the latter involves radical shifts that introduce functional innovations such as the ability to bind a completely different substrate or change an enzyme’s mechanism. While small adaptive changes may result from point substitutions introduced during evolution^3^, larger functional leaps may require more profound rearrangements of the protein backbone^4^. However, the potential for innovation comes at the price of disruption and destabilization. One way to harvest the effects of profound modifications is to infer ancestral enzymes^5^ with features that enhance evolvability: stability and promiscuity^6,7^. This enables the creation of robust generalist scaffolds that may be more catalytically versatile because they are less burdened by adaptive pressure towards a specific function than their modern-day counterparts^8,9^. If ancestors are indeed more stable, their capacity to buffer the large mutational load arising from backbone modifications^4^ may be enhanced.

Most directed evolution efforts rely on point substitutions. There have been far fewer reports of experimental insertions or deletions (InDels), despite their relatively frequent and beneficial occurrence in natural evolution^10^. Various genomic analyses show that the ratio of InDels to point substitutions in protein-coding regions typically ranges from 1:5 in primates to 1:20 in bacteria, indicating that InDels are typically subjected to stronger purifying selection than substitutions during evolution. This is due to their potentially more deleterious effects, including losses of stability, disruptions of secondary structure elements and/or perturbations of folding pathways^11^. To explore the potential effects of InDel mutagenesis on functional proteins, we previously developed TRIAD (transposition-based random insertions and deletions)^12,13^, a method for generating random InDel libraries that provides ready access to variants that cannot be obtained by substitution mutagenesis 10.21203/rs.3.pex-1448/v1. TRIAD is transposon-based and was shown to have only minimal sequence bias, with >85% of all possible sites shown to be targeted by the transposon^12^. In the present work, TRIAD is applied to a particularly stable ancestral protein^14^, the recently designed and characterized Anc^HLD-RLuc^ that was reconstructed from the catalytically distinct but evolutionarily and structurally related haloalkane dehalogenases^15^ (HLD, EC 3.8.1.5) and *Renilla* luciferase^16^ (RLuc, EC 1.13.12.5). This ancestor^14^ is bifunctional and catalytically versatile, with a dehalogenase activity comparable to contemporary HLDs and a promiscuous luciferase activity almost 7000-fold lower than that of the stabilized RLuc8, a popular molecular probe. The light-producing reaction catalysed by the *Renilla*-type luciferase is one of the most widely used biochemical reactions in molecular and cell biology research. Based on conformational differences of the RLuc8 backbone in crystal structures^17^, it had been suggested that the opening of the L9 loop might be relevant for luciferase substrate binding. The availability of Anc^HLD-RLuc^, a stable bi-functional scaffold^14^, enabled investigation of structure-function relationships of luciferase activity.

**Fig. 1:**
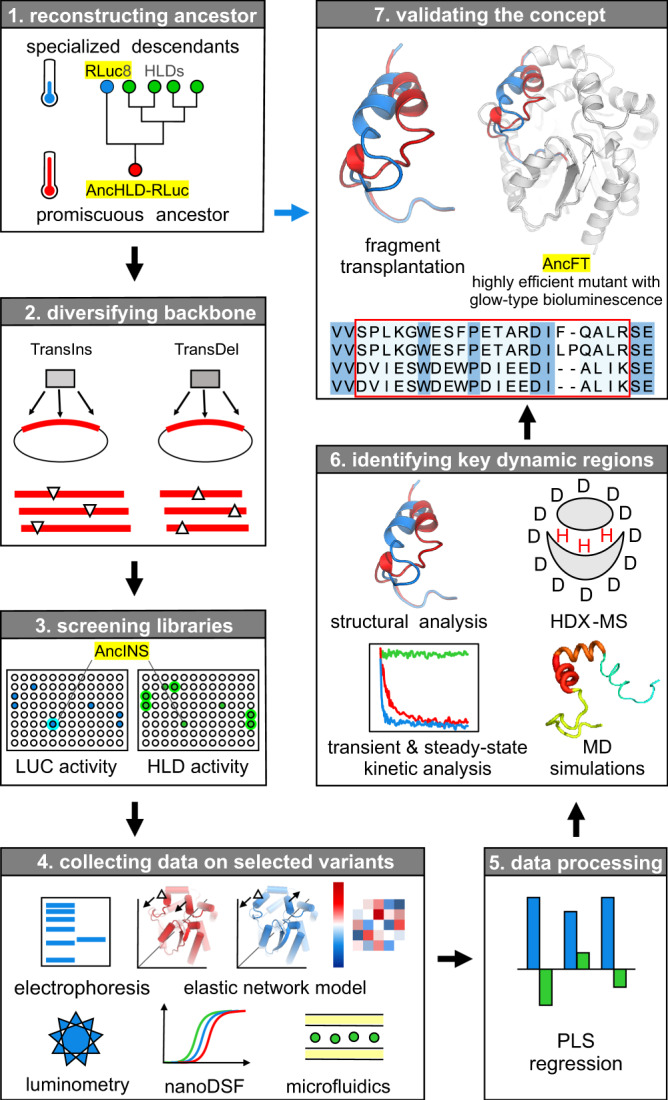
Illustration of the strategy for semi-rational engineering of protein dynamics. Exploratory phase—**1** A thermostable ancestral protein, Anc^HLD-RLuc^, provided a robust and evolvable template that can withstand the destabilizing effects of protein backbone engineering. **2** Libraries of single triplet insertion and deletion variants were created following the TRIAD method based on the use of engineered transposons (TransIns and TransDel)^12,13^. **3** Screening of the libraries led to identification of the improved insertion mutant AncINS. **4** Twenty-five mutants with significant changes in luciferase (LUC) and haloalkane dehalogenase (HLD) activities were characterized using bioinformatics, microscale techniques (nano differential scanning fluorimetry—nanoDSF), and microfluidics. **5** Structure-function relationships were described employing partial least squares (PLS) multivariate statistics. **6** Dynamic elements required for efficient catalysis were identified by structural, kinetic, biophysical and computational characterization (molecular dynamics (MD) simulations) of AncINS. Validation phase—**7** Knowledge obtained during the exploratory phase was validated by transplanting a relevant dynamic fragment from the specialized descendant into the ancestor, yielding an enzyme, AncFT, with 7000-fold higher catalytic efficiency than Anc^HLD-RLuc^ and 100-fold longer glow-type bioluminescence than the flash-type *Renilla* luciferase RLuc8. Key mutants discussed in this study are highlighted in yellow.

Here we present a three-step protein engineering strategy designed to exploit the effects of InDels as promotors of evolutionary innovation: (i) constructing a robust and evolvable template, (ii) mapping functional regions by backbone mutagenesis and multivariate statistics and (iii) transplantation of a dynamic structural (loop-helix) feature. Its application to the bifunctional protein Anc^HLD-RLuc^ (Fig. 1) results in an engineered 7000-fold more efficient catalyst with 100-fold longer glow-type bioluminescence applicable as a molecular probe for use in bacterial as well as mammalian cells.

## Results

### Structure–function relationships by analysis of InDel libraries

**Fig. 2:**
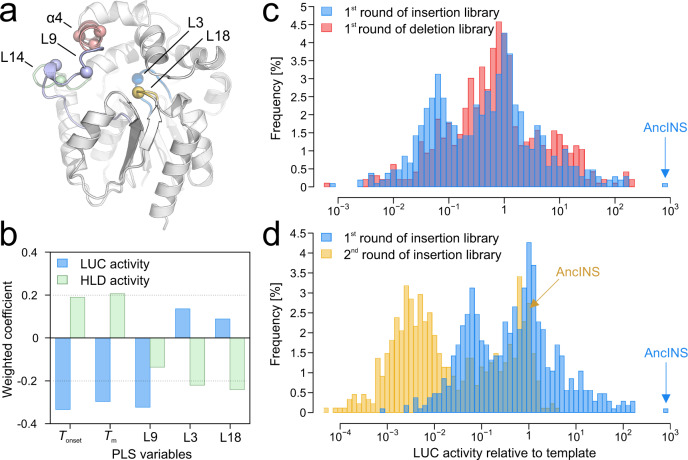
Quantitative structure-function relationships and analysis of the effects of template and mutation type on activity. **a** Crystal structure of the thermostable Anc^HLD-RLuc^ (PDB ID 6G75) showing the positions of the L9 loop (light blue), the α4 helix (salmon) and the L14 loop (pale green), where InDels (spheres) resulted in an increase of luciferase (LUC) activity. Correlated motions of L3 (marine) and L18 (yellow) loops carrying two of the five catalytic amino acids (spheres) were identified as significant contributors to dehalogenase (HLD) activity by the partial least squares (PLS) regression analysis. **b** Weighted coefficients quantifying contributions of variables indicated in the PLS models to explain the variance in HLD (green) and LUC (blue) activity. Note that different directionality of these coefficients for all variables, except loop L9, suggests different mechanistic and structural requirements for the two enzymatic functions studied. PLS generated models that explained substantial amounts of the variation in both LUC activity (*R*^2^ = 0.73, *Q*^2^ = 0.67, *n* = 25 is the number of InDel variants) and HLD activity (*R*^2^ = 0.63, *Q*^2^ = 0.54, *n* = 25 is the number of InDel variants). To obtain *Q*^2^ the model was recalculated 999 times with a randomly re-ordered dependent variables. **c** Comparison of frequencies of variants with their activities relative to the ancestral protein in the first-round insertion (R1I, blue) and deletion (R1D, red) libraries. The template for R1I and R1D was Anc^HLD-RLuc^. The best insertion variant AncINS is indicated by an arrow. **d** Comparison of frequencies of variants with indicated activities relative to their respective templates observed in the first round of insertion library (R1I, blue) and the second round of insertion library (R2I, yellow). Note that the starting template Anc^HLD-RLuc^ for R1I and R1D is less active than the starting template AncINS for R2I. The template for R2I was AncINS. Source data is available as a Source data file for Fig. 2.

Libraries of Anc^HLD-RLuc^ with random single amino acid insertions (1st round, R1I) and deletions (1st round, R1D) distributed over the length of the protein were constructed using TRIAD^12^ and screened for LUC and HLD activities (Supplementary Note 1). All hits with significantly improved LUC activity had backbone alterations localized in one of three regions of the enzyme cap domain: the L9 loop, the α4 helix or the L14 loop (Fig. 2a, Supplementary Table 1). We selected 11 variants (10 from R1I, 1 from R1D) for further analysis and complemented them with 15 variants with low LUC activity that retained HLD activity (8 from R1D and 7 from R1I, Source data file). All variants were expressed, purified and subjected to activity and stability measurements and thermodynamic analyses using a microfluidic approach (Supplementary Fig. 1). To understand the role of cooperativity and long-range interactions after mutagenesis, we used an anisotropic network model (ANM, Supplementary Note 2) to calculate the cross-correlation of motions of selected structural fragments (Supplementary Tables 2, 3, Supplementary Note 3) to the regions carrying the InDel mutations of the selected variants.

Multivariate partial least squares (PLS) regression (Supplementary Note 4) revealed that the initiation of protein unfolding (*T*_onset_) and its midpoint (*T*_m_) were the strongest predictors for both LUC and HLD activities. At *T*_onset_ (0.5% deviation from a linear fit on the 330 nm/350 nm vs. temperature curve) a protein starts to unfold, while at *T*_m_ (inflection point on the 330 nm/350 nm vs. temperature curve) 50% of the protein is unfolded. The directionality of the relationships was the opposite for HLD and LUC activities (Fig. 2b). This suggested that stabilities are linked, positively and negatively, to the two activities with their distinct chemical mechanisms and structural requirements. Moreover, cross-correlated motions of the mutated positions to the L9 loop were identified as very strong contributors to LUC activity, and cross-correlated motions to the L3 and L18 loops (which encompass catalytic residues^14,18^) as key contributors to HLD activity.

All the positive hits had significantly reduced thermal stability compared to the ancestral protein (up to –20 °C lower *T*_onset_ and *T*_m_ values; Source data file), in agreement with the notion that a stable template is crucial for obtaining functional mutants. Moreover, we assumed that using a highly stable starting point for engineering would promote evolvability by allowing the protein to accept a wider range of mutations while retaining its native fold^19^, thereby enabling multiple rounds of directed evolution. To probe this hypothesis, the best variant from R1I library (AncINS) carrying an insertion and substitution in the α4 helix was used as the template for construction of another amino acid insertion library (2nd round, R2I) that was screened for further improvement in LUC activity. Statistical analysis of activity data shows that R1I and R1D yielded populations of variants with similar LUC activity patterns, and most of the increases in relative activity obtained in these rounds were compromised in R2I (Fig. 2c, d). The proportions of variants showing at least doubled activity of the starting template generated in R1I, R1D and R2I were 13%, 15.5% and just 0.5%, respectively. As no significant activity improvements were obtained in the second round of insertion mutagenesis, no further rounds were pursued.

The finding that a single round of InDel mutagenesis of the stabilized template already provided mutants with >100-fold increases in LUC activity raises the question: why are the structural elements (L9 and L14 loops and α4 helix), highlighted by directed evolution experiments, crucial for LUC activity? To address this question, the mutant AncINS, carrying an insertion in the α4 helix, was selected as a representative and studied by transient and steady-state kinetics.

### Kinetic analysis underlines the importance of conformational flexibility for substrate binding

**Fig. 3:**
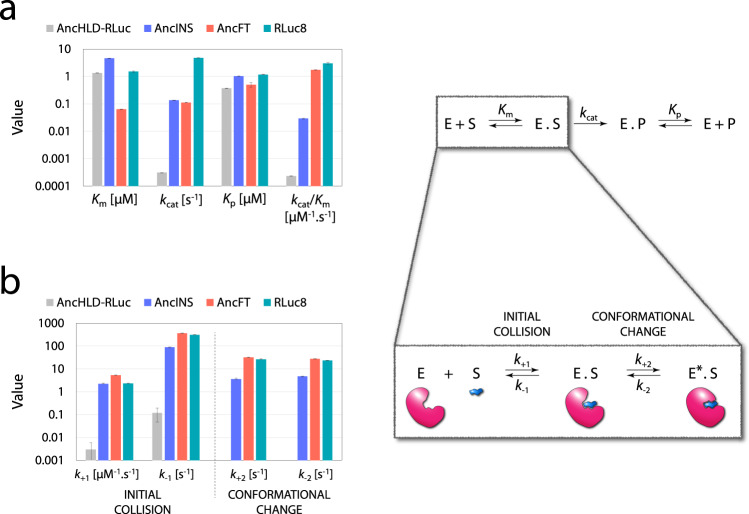
Steady-state and transient kinetic analysis of CTZ conversion. The kinetic models consist of an enzyme E, a substrate S, an enzyme-substrate complex in two conformations (E.S and E*.S), an enzyme-product complex E.P, and a product P. **a** Steady-state kinetic parameters (Michaelis constant *K*_m_, turnover number *k*_cat_, enzyme-product complex dissociation constant *K*_p_) were determined with the substrate CTZ in 100 mM phosphate buffer at pH 7.5 and 37 °C by global analysis of triplicates of full progress curves recorded with at least five concentrations of CTZ. **b** Results of pre-steady-state kinetic analysis of the CTZ substrate binding in 100 mM phosphate buffer at pH 7.5 and a lower temperature (15 °C). This enabled identification of the induced fit substrate binding mechanism, involving initial collision of the enzyme and the substrate (described by forward rate constant *k*_+1_ and reverse rate constant *k*_−1_), followed by a conformational change of the enzyme induced by the bound substrate (described by forward rate constant *k*_+2_ and reverse rate constant *k*_−2_). A simple binding mechanism including only the first step was observed for Anc^HLD-RLuc^. The kinetic parameters were determined by global fitting of tryptophan fluorescence traces obtained with at least 10 concentrations of CTZ and 10 concentrations of each tested enzyme, in each case with seven replicates (Supplementary Note 6). The data are presented as best fit values ± standard errors (S.E.) calculated from the covariance matrix during nonlinear regression. Source data is available as a Source data file for Fig. 3.

To gain insight into the catalytic mechanism, steady-state kinetics of LUC activity of the three luciferases (AncINS, the template Anc^HLD-RLuc^ and the modern variant RLuc8; Fig. 1). were measured with the substrate coelenterazine (CTZ). Complete luminescence progress curves (Supplementary Fig. 5) gave, by numerical simulation, estimates of the turnover number (*k*_cat_) and the enzyme specificity constant (*k*_cat_/*K*_m_). *k*_cat_ provides a lower limit for each first-order rate constant following binding of a substrate through product release, while *k*_cat_/*K*_m_ indicates a lower limit of the rate for substrate binding (Fig. 3a). The parameters obtained for RLuc8 correspond well with previously reported values derived from initial rate measurements combined with quantum yield calibration (Supplementary Table 4)^20^. Moreover, following a time course of the substrate-to-product conversion, our data provide a sensitive estimate of the equilibrium dissociation constants for each enzyme–product complex (*K*_p_) that cannot be obtained by conventional initial rate analysis. Both *k*_cat_ and *k*_cat_/*K*_m_ values of AncINS were in-between those of Anc^HLD-RLuc^ and RLuc8, indicating a 124-fold enhancement of its catalytic efficiency relative to that of the ancestral enzyme.

We also investigated by pre-steady-state kinetic analysis, whether conformational changes of the protein are essential for effective binding of the bulky CTZ substrate (Fig. 3b). The combination of analytical data processing and global fitting by numerical integration revealed a two-step induced fit substrate-binding mechanism: initial collision of the enzyme with the substrate, followed by an induced conformational change of the enzyme (Fig. 3b, Supplementary Fig. 6). Details and the rationale of the mechanistic analysis are described in the Supplementary Information (Supplementary Note 5). The kinetic constants are consistent with a model in which the reaction catalysed by Anc^HLD-RLuc^ involves a slow simple binding mechanism, with no sign of conformational flexibility. By contrast, we detected conformational flexibility of AncINS, accompanied by 1000-fold faster binding, based on determined *k*_+1_ and *k*_-1_. The changes in AncINS increased rates of the initial collision step to match those of RLuc8, but a subsequent conformational change was still 10-fold slower. Faster kinetics of substrate binding after backbone modification in the α4 helix suggest that this region plays a significant role in the initial step of the catalytic cycle.

### Backbone conformational differences in crystal structures

**Fig. 4:**
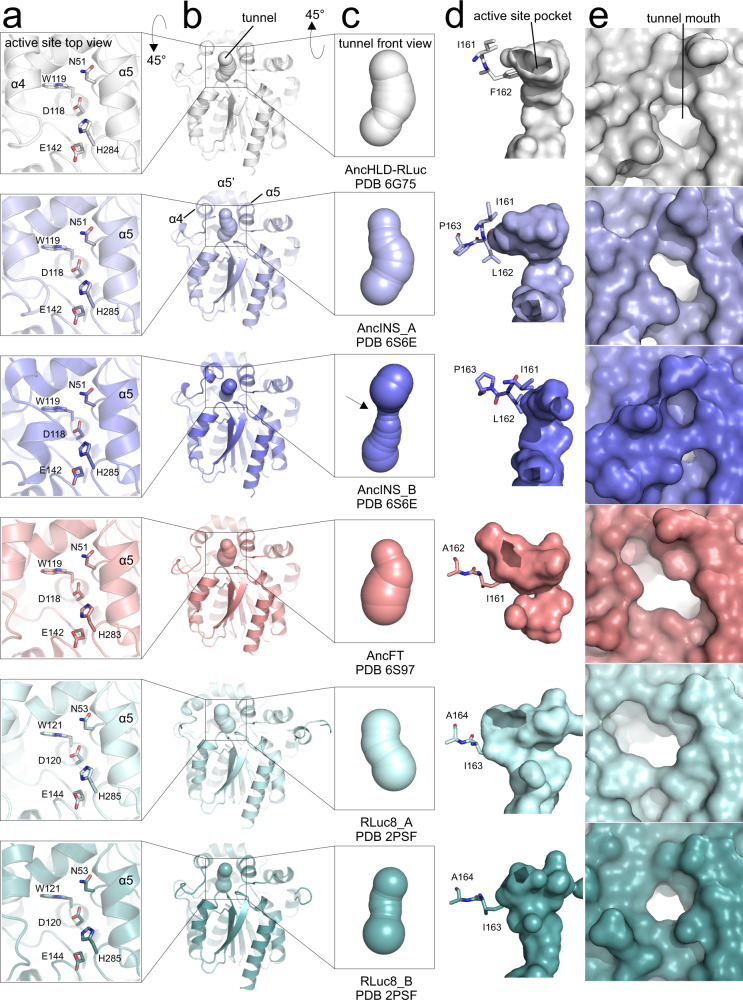
Comparison of structural changes in the apo forms of the enzymes. Anc^HLD-RLuc^ (white), AncINS (chain A light blue, chain B slate), AncFT (salmon), RLuc8 (chain A pale cyan, chain B light teal). **a** Top view of the active site. The putative catalytic pentad and α5 helix occupy the same position in all proteins, but the α4 helix adopts different conformations. **b** Crystal structure of the whole protein, with the main access tunnel (identified using Caver 3.02^23^) shown as spheres. The outward/inward movement of the α4 helix causes opening/closing of the tunnel. **c** Front view of the main tunnel. The black arrow indicates the visible constriction due to closure of the α4 helix. **d** The size of the active site cavity is changed by repositioning of amino acids in the α4 helix. **e** The size of the main tunnel mouth is changed by movements of the α4 helix and L9 loop.

To investigate the relationship between the structural changes induced in Anc^HLD-RLuc^ by InDel mutagenesis and enhanced kinetics of substrate binding, we crystallized AncINS (PDB ID 6S6E, Supplementary Table 6) and compared its structure to previously published structures of Anc^HLD-RLuc^ (PDB ID 6G75) and RLuc8 (PDB ID 2PSF). These proteins have identical α/β-hydrolase folds but differ in their cap domains. Important changes were identified in the conformation of the α4 helix and L9 loop in the cap domain, size of the active site cavity, width of the tunnel mouth, and active site accessibility (Fig. 4).

The asymmetric unit of the crystal lattice of AncINS contains two monomers (chains A and B). Chain A has a structure similar to the Anc^HLD-RLuc^ template but features a π-helix bulge in the α4 helix where the L162 insertion and F163P substitution occurred. In AncINS chain B, the α4 helix is markedly distorted towards the α5 helix. Moreover, the electron density map for the L9-α4 fragment is not perfectly resolved. Some side chains are poorly visible or invisible, suggesting that appreciable internal motion occurs in this region. RLuc8 also has two monomers in the asymmetric unit; chain B is similar to that of Anc^HLD-RLuc^, while chain A is in an open conformation with the α4 helix pointing away from the α5 helix.

In AncINS, the inserted L162 occupies the same position as the bulky F162 in Anc^HLD-RLuc^, making the active site cavity of AncINS bigger than that of Anc^HLD-RLuc^. The F163P substitution in AncINS disrupts the α4 helix because proline acts as a helix breaker, introducing a kink into the polypeptide backbone. The resulting distortion closes the main access tunnel in the chain B of AncINS. In RLuc8, the situation is a little bit more complex. The sequence alignment (Supplementary Fig. 9) indicates that I163 is a conserved amino acid, but it does not occupy the same position as I161 in Anc^HLD-RLuc^. Instead, it is flipped down into the position occupied by the bulky F162 in Anc^HLD-RLuc^. The outward conformation of the α4 helix in the cap domain of RLuc8 chain A gives it the largest active site cavity of the crystal structures studied.

**Fig. 5:**
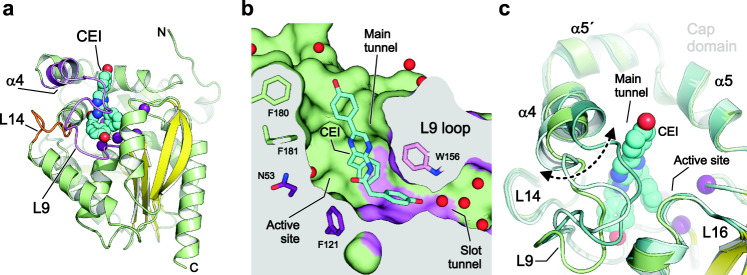
Structural characterization of coelenteramide binding to the catalytically defective RLuc8-W121F/E144Q mutant. **a** Cartoon representation of the overall structure of the RLuc8-W121F/E144Q mutant with coelenteramide (CEI) in its active site. CEI is the product of the LUC reaction and shown as cyan space-filling spheres. Residues of the conserved catalytic pentad are shown as purple spheres; the central eight-stranded β-sheet is coloured yellow; the α4 helix and L9 loop (L9-α4 element) are coloured violet, and the L14 loop is coloured orange. **b** Cutaway surface representation of the enzyme active site cavity with the bound CEI (shown as cyan sticks). The colouring is the same as in panel (**a**). Water molecules are shown as red spheres. **c** Close-up view of structural superposition of RLuc8-W121F/E144Q (green), RLuc8 (PDB ID 2PSF A; cyan) and RLuc8 (PDB ID 2PSF B; teal). CEI is shown as cyan space-filling spheres. Note the conformational sampling of the L9-α4 fragment. The bottom 4-hydroxyphenyl group connected to the CEI acetamide moiety is deeply buried in the active site cleft, where it is anchored in the slot tunnel through multiple hydrophobic (P224, I223 and I266) and aromatic π-stacking (W156) interactions. The 4-hydroxyphenyl group interacts with the indole NH group of W156 through a water-mediated hydrogen bond bridge. The acetamide moiety of CEI is positioned close to the conserved catalytic centre. In chain B, the top 4-hydroxyphenyl group linked with the CEI pyrazine ring interacts with a side chain of K189 through a water-mediated hydrogen bond bridge and forms a hydrogen bond with the carboxylate group of D162 from a symmetry-related enzyme molecule.

To ascertain the role of the L9-α4 fragment in substrate binding, we tried to co-crystallize RLuc8 with CTZ. Despite our best efforts, we did not obtain sufficiently well diffracting crystals. In contrast, mixing the RLuc8-W121F/E144Q mutant with CTZ yielded a high-resolution complex structure (Fig. 5a; Supplementary Table 6) with coelenteramide (CEI), which is the product of the LUC reaction. Unlike in an RLuc8-CEI complex structure^17^, the CEI molecule in our complex is inverted by nearly 180°, which allows its accommodation in the luciferase active site cavity (Fig. 5b, Supplementary Fig. 10). The rest of the CEI molecule occupies the main enzyme access tunnel, where it is tightly wrapped by multiple, predominantly hydrophobic, residues: V146, I150, I159, V185, F181, F261, F262, H285 and I266.

Interestingly, many of the residues directly interacting with the CEI are located on the loop L9 (V146, I150, W156 and I159) and the loop L14 (I223 and P224), identified as important for catalysis by our directed evolution experiments. The α4 helix is in an open conformation relative to the α5 helix (Fig. 5c). Ultimately, the L9-α4 fragment directly affects the opening and closing of the access tunnels and we deduce that it is involved in substrate binding and product release. Thus, our structural data support conclusions from InDel mutagenesis and kinetic studies that the L9-α4 fragment is the key hotspot region for the introduction of LUC activity.

### Profiling protein dynamics by mass spectrometry and molecular dynamics

**Fig. 6:**
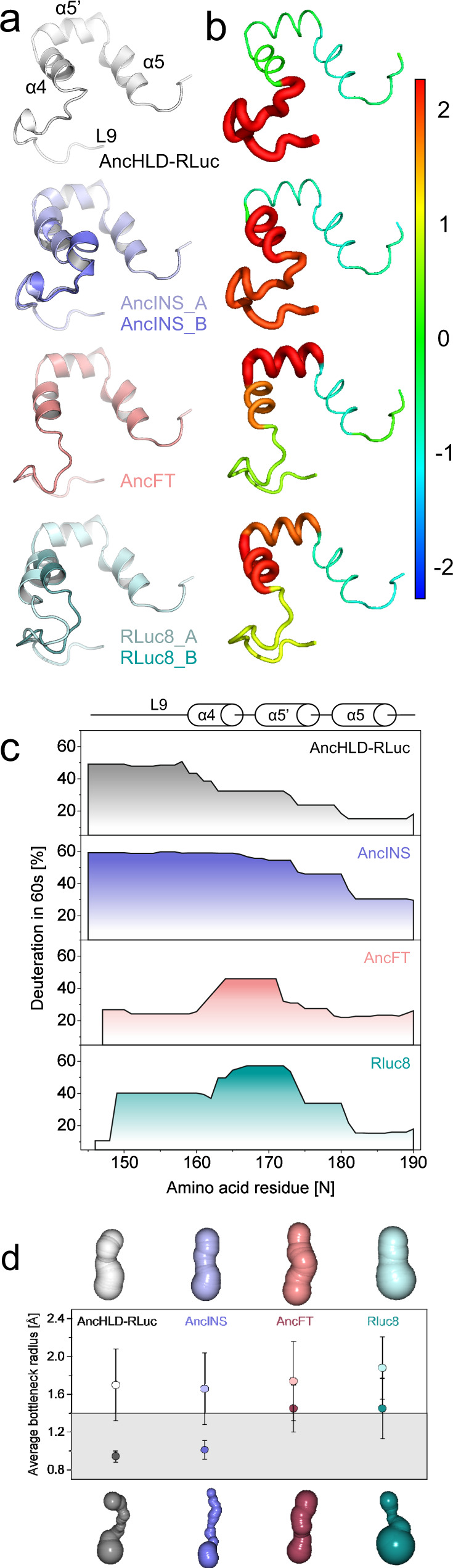
Engineering conformational dynamics by backbone modifications. **a** Conformational dynamics in the cap domains of crystal structures: AncINS (PDB ID 6S6E) has two monomers in the asymmetric unit differing in conformation of the α4 helix carrying the insertion. Chains A (light blue) and B (slate) are in the open and closed conformations, respectively. RLuc8 (PDB ID 2PSF) has analogous conformations, with an open chain A (pale cyan) and a closed chain B (light teal). AncFT (PDB ID 6S97) has one monomer in the asymmetric unit, in which helix α4 is more open than in Anc^HLD-RLuc^, AncINS, and even RLuc8 chain B. **b** Conformational dynamics in the cap domains observed during molecular dynamics simulations. B-factors of backbone atoms standardized across all protein variants, ranging from −2 (blue) to 2 (red). B-factor values standardized for each protein are indicated by the thickness of the lines representing the protein backbone. Values were averaged per secondary structure element. **c** Dynamics and hydration of the cap domain based on HDX-MS assessments of peptide deuteration after 60 s. Anc^HLD-RLuc^ is most heavily deuterated in the L9 loop. The deuteration pattern of AncFT is most similar to that of RLuc8. **d** Main access tunnel geometries of the proteins: representative snapshots of the open (lighter shades, upper row) and closed (darker shades, lower row) conformations. Average bottleneck radii and standard deviations from *n* = 1000 snapshots from MD simulations were calculated by Caver 3.02^23^. The grey area corresponds to closed conformational states with tunnel radii below 1.4 Å (the radius of a water molecule). Source data is available as a Source data file for Fig. 6b and c.

To analyse the effects of dynamics on LUC activity, we recorded hydrogen-deuterium exchange mass spectrometry (HDX-MS) time courses and complementary molecular dynamics (MD) simulations (Fig. 6). In this context, it should be emphasized that HDX-MS reveals changes occurring over seconds or minutes, whereas MD simulations cover a few microseconds (in this case 4.8 μs). Kinetic analyses were carried out with the substrate, while HDX-MS and MD simulations were performed in the absence of any ligand. HDX-MS captures both dynamics and solvation.

Backbone amide hydrogen-deuterium exchange is particularly sensitive to hydrogen bonding and thus reflects hydrogen bonding strength, conformational dynamics, and the solvent accessibility of protein structures. The HDX-MS profiles (Fig. 6c, Supplementary Fig. 11) show that AncINS was deuterated more rapidly overall than the template Anc^HLD-RLuc^, and that the modification of its α4 helix backbone leads to greater solvation and more pronounced dynamics, particularly in the cap domain region (Supplementary Fig. 11). Moreover, AncINS exhibited very high overall deuteration within 10 and 60 s, with the amino acids between residues W151 (L9 loop) and F180 (α5 helix) of the cap domain being most extensively deuterated. Residues S212-E235 (which comprise the L14 loop) were also appreciably more deuterated than their counterparts in Anc^HLD-RLuc^. The deuteration of the α4-α5 fragment in RLuc8 reached a deuteration level comparable to the level observed in AncINS within 60 s, but it increased further in 300 s. In addition, the L9 loop of RLuc8 was less extensively deuterated than the corresponding loop in Anc^HLD-RLuc^ and the region S212-E235 (L14 loop) of RLuc8 was more heavily deuterated than in Anc^HLD-RLuc^. These deuteration patterns in HDX-MS indicate a possible correlation between the dynamics of hot spot regions comprising the α4 and α5′ helices and L14 loop and high LUC activity. The protein dynamics are coupled to efficient binding of the bulky substrate and release of the structurally similar product, based on the kinetic constants *k*_+1_ and *k*_-1_ obtained by the pre-steady state kinetics (Fig. 3b, Supplementary Fig. 6).

The regions encompassing the L9-α4 fragment and L14 loop are important structural elements that line the main access tunnels connecting the buried active site to the surrounding solvent. Their motions are concerted based on ANM and significantly affect the active site’s volume, while fully preserving the catalytic residues’ geometry. Since HDX-MS experiments measure the exchange of backbone amide hydrogens with deuterium, we used backbone B-factors from MD simulations to characterize the enzymes’ dynamics^14,21^. The overall B-factors obtained for the cap domain of RLuc8 were highest in the α4 helix, followed by the α5′ helix and L9 loop (Fig. 6b). The B-factors for the L14 loop were similar to those of the L9 loop, but values for the rest of the cap domain were below average (Supplementary Fig. 12). The dynamic profile of the Anc^HLD-RLuc^ template differs significantly: unlike in RLuc8, the dynamics are most pronounced in the L9 loop. Interestingly, the AncINS mutant exhibits markedly higher B-factors in the α4 helix, making it the most dynamic region together with the L9 loop (Fig. 6b).

### Validation by fragment transplantation yields stable glow-type bioluminescence

To verify the importance of the hotspot regions highlighted by biophysical analysis, we transplanted the sequence corresponding to the L9-α4 fragment from the modern RLuc8 enzyme into the ancestral Anc^HLD-RLuc^. The resulting AncFT protein had lower thermal stability than the ancestral variant (with ca. 7 °C lower *T*_m_ value) but folded correctly (with a CD spectrum similar to that of Anc^HLD-RLuc^ and AncINS; Supplementary Fig. 13). We also constructed a second variant with L14 transplanted from RLuc8 onto the ancestral scaffold, but this protein easily aggregated, preventing its biochemical characterization.

**Fig. 7:**
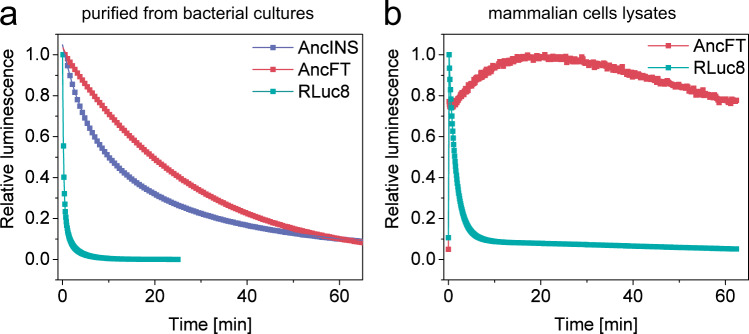
Comparison of bioluminescence of enzymes purified from bacterial cultures and in lysates from mammalian cells. **a** Full decay kinetics of the conversion of 2.2 μM CTZ by 50 nM of RLuc8, AncINS and AncFT purified from bacterial cultures. The data are presented as relative values to initial luminescence; Anc^HLD-RLuc^ is not plotted due to low activity leading to large signal scattering. Solid lines represent the best fit to the experimental data. Experiments were repeated independently three times with consistent results. **b** Bioluminescence signal steadiness in relative values in lysates from mammalian cells expressing AncFT and RLuc8. Experiments were repeated independently three times with consistent results. Activity was measured using the commercial *Renilla* luciferase assay kit (Promega) with 20 μL of cell lysates and 100 μL of assay buffer. Luminescence signal in lysates from mammalian cells expressing Anc^HLD-RLuc^ and AncINS was not detectable under tested conditions. Source data is available as a Source data file for Fig. 7.

Transplantation of the L9-α4 flexible region from RLuc8 into the scaffold of Anc^HLD-RLuc^ affected substrate-binding kinetics (Fig. 3): AncFT was found to have comparable initial collision kinetics to AncINS and RLuc8, but the fragment transplantation enhanced the following conformational change and (unlike AncINS) AncFT reached the overall binding efficiency of the modern luciferase. These findings correspond well with the results of the steady-state analysis. Similar values of the specificity constant *k*_cat_/*K*_m_ were obtained for AncFT and RLuc8, indicating comparable efficiency of substrate binding. Thus, AncFT’s lower LUC activity is due to a lower rate of the chemical transformation (i.e., the step after binding), as indicated by a 10-fold lower *k*_cat_ value for AncFT compared to RLuc8 (Fig. 3a, Supplementary Table 4). On the other hand, AncFT exhibited markedly weaker product inhibition than RLuc8. This was apparent from a significantly lower *K*_m_/*K*_p_ ratio, quantifying a binding of the substrate and the product, which has an important effect on the bioluminescence signal’s stability. The significant product inhibition of RLuc8, indicated by the high *K*_m_/*K*_p_, is one of the reasons why the bioluminescence signal provided by the luciferase rapidly decays after a strong initial flash. The fast inactivation and signal instability are major limitations of this popular molecular probe. Re-engineering of RLuc8 by ancestral reconstruction and subsequent backbone modification changed the unstable flash-type of bioluminescence to a significantly more stable glow-type bioluminescence. The half-life (*t*_1/2_) of AncFT bioluminescence was two orders of magnitude longer than that of RLuc8 (Supplementary Table 7, Fig. 7a, Supplementary Fig. 14), being consistent with a significantly lower *K*_m_/*K*_p_ ratio determined for AncFT, in comparison to RLuc8. The highly stable glow-type bioluminescence of AncFT persisted when this protein was expressed heterologously in mammalian (mouse fibroblast) cells (Fig. 7b, Supplementary Fig. 14). This result indicates that AncFT could be used as a reporter protein^22^ for in vivo experiments.

Crystallographic analysis of AncFT (PDB ID 6S97, Supplementary Table 6) revealed a canonical α/β-hydrolase fold similar to that of Anc^HLD-RLuc^, but with some structural features reminiscent of RLuc8. Most importantly, both the active site cavity and the α4 helix conformation of AncFT are more open than in Anc^HLD-RLuc^, AncINS, and even the RLuc8 chain B (Fig. 4). Chain A of RLuc8 (PDB ID 2PSF) has the most open conformation seen in any of the structures (Figs. 4, 6a).

HDX-MS experiments showed that the deuteration pattern of AncFT was almost identical to that of Anc^HLD-RLuc^, with differences only in the transplanted region (Fig. 6c, Supplementary Fig. 11). Specifically, the end of the L9 loop was less extensively deuterated than in Anc^HLD-RLuc^ while the α4-α5′ helices were more heavily deuterated. A similar trend was observed for RLuc8, providing experimental evidence that cap domain dynamics of the mutant with the transplanted fragment mimic those of RLuc8 (Fig. 6c).

MD simulations of AncFT revealed that the B-factor profile of its cap domain closely resembled that of RLuc8 (Fig. 6b). The B-factors were highest in the α5′ and α4 helices, while those of the L9 and L14 loops were close to the average for the rest of the enzyme and the rest of the cap domain had lower values (Supplementary Fig. 12). Tunnel analysis using Caver^23^ showed that tunnel dynamics of Anc^HLD-RLuc^ and AncINS clearly differ from those in AncFT and RLuc8 (Fig. 6d). The former two enzymes can adopt conformations with closed tunnels (average bottleneck radii: 0.9 and 1.0 Å, respectively), and conformations with open tunnels (average bottleneck radii: 1.7 Å in both cases). We consider a tunnel to be closed when its bottleneck radius drops below the radius of a water molecule (1.4 Å). In contrast, there is a clear overlap in bottleneck values calculated for the extremes of the conformational spectra of AncFT and RLuc8: the conformations that form the narrowest tunnels have identical average bottleneck radii of 1.5 Å, while those forming open tunnels have average bottleneck radii of 1.7 and 1.9 Å, respectively (Fig. 6d).

In addition, we performed an analysis of the cavity size of the proteins to test whether changes in the catalytic properties correlate with the changes in the volume of the active site. When analysing 100 random snapshots from MD simulations we noticed that cavity volumes of all proteins can adopt wide ranges, but they also converge to similar minimal volume of 200 Å^3^ in the closed state. The volumes calculated from the simulations starting from the open and closed conformations showed different absolute values, except for AncFT, which does not have multiple monomers in the asymmetric unit (Supplementary Fig. 15). Neither analysis of volumes of the static structures nor 100 representative snapshots obtained from MD simulations show any sign of correspondence between the catalytic characteristics and the active site volumes. Furthermore, on interpreting whether the system dynamics or the cavity volume is more important for the catalytic activity, the analyses of the ANM presented above agree with the importance of the dynamics.

## Discussion

The present work validates a strategy for engineering enzyme backbone dynamics based on InDel mutagenesis of a stable (high melting temperature) and catalytically promiscuous (evolvable) template. We used TRIAD^12^ mutagenesis to generate single amino acid insertion and deletion libraries of a stabilized bi-functional ancestral enzyme^14^ of haloalkane dehalogenase and *Renilla*-type luciferase enzymes (Fig. 1). Alternatively, other published methods for introducing insertions and deletions could be used^24–27^. The most potent insertion variant, AncINS, has both an insertion and a substitution in the α4 helix. These mutations markedly improved substrate binding, linked to changes in dynamics of the cap domain, but not the putative catalytic pentad. Remarkably, this was achieved in a single round of mutagenesis. In contrast, routinely used substitution mutagenesis protocols often require multiple time-consuming rounds of directed evolution and high-throughput screenings^28–30^. Systematic mutagenesis strategies, that allow a single-round selection to identify regions of interest for more focused study, are available^31^. In the future, maximizing information obtained from the selection through deep sequencing of gene libraries^32^ may also provide information to predict beneficial mutations based on a single round.

The crystal structure of the insertion variant AncINS (PDB ID 6S6E) revealed that its asymmetric unit contains two monomers with different spatial arrangements of the α4 helix (implying they represent two conformers), and MD simulations confirmed that the mutations caused substantial structural rearrangements, most probably because the substituted residue P163 acts as a helix breaker. We conclude that the LUC activity benefits from conformational dynamics of the protein’s rigid scaffold to allow binding of a bulky substrate. The outward movement of the α4 helix has an immediate effect on the opening of the access tunnel and increasing of the buried cavity’s volume, thus facilitating binding of the bulky substrate CTZ. However, once CTZ is bound inside of the active site cavity, the protein has to provide tight binding of the molecule inside it and provide a desolvated environment for the subsequent monooxygenation reaction and bioluminescence to occur^33,34^. If the excited product of the reaction, CEI, would not be tightly bound, the energy would probably not be released in the form of light during decay to the ground state, but in another form, e.g., heat. Accordingly, we propose that the evolutionary processes that generated the modern *Renilla* luciferase altered its dynamics in a way that facilitated substrate binding to the open state. While the movement of the α4 helix was shown to be important for CTZ binding, the dynamic profile of AncINS, as determined by HDX-MX experiments and MD simulations, still differs from that of RLuc8. To validate the proposed conclusions, we transplanted the α4 helix and L9 loop from the highly efficient modern RLuc8 into the poorly active ancestral Anc^HLD-RLuc^. The resulting enzyme, AncFT, was highly efficient, with significantly decreased product inhibition compared to RLuc8, providing glow-type luminescence. Crystallographic analysis revealed that its structure (PDB ID 6S97) was more open than those of both the ancestral protein and B chain of RLuc8, facilitating the entrance of the bulky substrate. Based on MD simulations and HDX-MS experiments, we conclude that the transplantation reduced motions of the L9 loop, but increased motion of the α4-α5′ helices, making the mutant protein’s dynamics more similar to RLuc8.

Our results provide experimental evidence for the role of protein dynamics in enzymatic catalysis. An ancestral protein (Anc^HLD-RLuc^) with sub-optimal dynamics has its catalytic activity limited by substrate binding, while mutants with tailored dynamism resulting from insertion/substitution (AncINS) or fragment transplantation (AncFT) have catalytic activity limited by the subsequent step of the chemical reaction. Transplantation of the most dynamic region of the extant RLuc onto the ancestral Anc^HLD-RLuc^ improved its catalytic efficiency 7000-fold to match the catalytic efficiency of RLuc, while extending the half-life of its light output 100-fold. Long-lasting glow-type light emission of AncFT was confirmed in mammalian cells, which paves the way towards its use as an efficient molecular reporter or biosensor^22^. Given the growing sensitivity of optical devices, signal stability is becoming a key requirement for modern molecular probes. In comparison with the short-term flash type of signal, a stable glow signal enables continuous detection in long-term biological experiments or maintenance of a stable response required during high-throughput screening campaigns^22^.

Thus, both the introduction of InDels and transplantation of dynamic elements into stable ancestors appear to be viable protein engineering strategies for improving enzymes or introducing novel functions. The potential effectiveness of grafting dynamic loops to introduce novel enzymatic functions has been discussed^35–37^ and experimentally demonstrated by the successful introduction of β-lactamase activity into the αβ/βα metallohydrolase scaffold^38^, thereby generating an enzyme that lacked its original activity but catalysed hydrolysis of cefotaxime. The potential of loop remodelling for engineering enzyme functions has also been illustrated by the deletion of a specific loop and introduction of a point mutation, leading to emergence of homoserine lactonase activity in a phosphotriesterase^39^. The potential of loop modification in enzyme engineering has also been discussed in several recent reviews^35–37^.

There are several preconditions necessary for the application of our engineering strategy. Ancestral sequence reconstruction requires about 150 sequences^40^, but a phylogenetic tree can also be reconstructed with a lower number of sequences. Mutational events leading to insertions and deletions make the reconstruction less reliable^41^. The InDel mutagenesis strategy is applicable to any protein of interest, but a screening assay is necessary for probing a sufficient number of mutants to identify the regions of interest and to allow robust statistical analysis. For the loop transplantation strategy, structural information about the proteins is necessary. The strategy is applicable for engineering catalytic efficiency, substrate specificity, and selectivity, and may enable the design and evolution of multifunctional enzymes. It is specifically suitable for enzymes with active sites flanked by loops and enzymes with reaction rates limited by substrate binding or product release.

In summary, we have developed a strategy for engineering backbone dynamics based on InDel mutagenesis of a stable template, multivariate statistics of the data from microscale and microfluidic experiments, and transplantation of a highly dynamic element. We validated its utility by an engineering effort in which catalytic efficiency was increased, and the bioluminescence was stabilized to achieve a long-lasting glow-type signal. The results achieved support the conceptual ideas, which guided our experiments and stepwise implementation of the strategy. The developed catalyst can serve as a molecular probe in bacterial and mammalian cells. The strategy may provide a useful addition to the repertoire of methods available for engineering catalytically efficient enzymes^40,41^ by exploring less travelled parts of protein sequence space. The loop transplantation strategy is being implemented in the web application LoopGrafter, which will make it accessible to a broad community (https://loschmidt.chemi.muni.cz/loopgrafter/).

## Methods

### Reagents and procedures used in TRIAD

FastDigest restriction endonucleases, MuA transposase and T4 DNA ligase were purchased from Thermo Fisher Scientific. DNA Polymerase I, Large (Klenow) Fragment, was purchased from New England Biolabs. All DNA modifying enzymes were used according to the manufacturer’s conditions. All DNA purification procedures were performed according to the manufacturers’ instructions using kits, including GeneJET Plasmid Miniprep kit (Thermo Fischer Scientific) for plasmid extractions from *E. coli* cells, Zymoclean Gel DNA Recovery kit (Zymo Research) for agarose gel extraction of DNA fragments upon electrophoresis and DNA Clean & Concentrator kit (Zymo Research) for DNA purification and concentration. Bacterial transformations were performed by electroporation using *E. cloni* 10G ELITE electrocompetent cells (Lucigen).

### Generation of insertion and deletion libraries of Anc^HLD-RLuc^ using TRIAD

InDel libraries were prepared following the TRIAD^12,42^ method. The sequence encoding Anc^HLD-RLuc^ was subcloned from pET21b::*anc*^*HLD-RLuc*^ to the TRIAD-dedicated vector pID-Tet using NdeI and BamHI and yielding pID-Tet::*anc*^*HLD-RLuc*^. This construct was used as template for the generation of transposition insertion libraries with engineered TRIAD transposons TransDel and TransIns. The transposons (~1 kbp) were extracted from pUC57 by BglII digestion and recovered by gel electrophoresis and purification. Insertion of TransDel or TransIns in pID-Tet::*anc*^*HLD-RLuc*^ (pID-Tet plasmid: ~2.7 kbp; *anc*^*HLD-RLuc*^: ~950 bp) was performed by in vitro transposition using ~300 ng of plasmid, ~50 ng of transposon and 0.22 μg MuA transposase in a 20 μL reaction volume. After incubation for 2 h at 30 °C, the MuA transposase was heat-inactivated for 10 min at 75 °C. DNA products were purified and concentrated in 7 μL deionized water. Two microlitres of the purified DNA was used to transform *E. cloni* 10G ELITE electrocompetent cells by electroporation. The transformants (~30,000–50,000 CFU) were selected on LB agar containing ampicillin (amp; 100 μg/mL) and chloramphenicol (cam; 34 μg/mL). The resulting colonies were pooled, and their plasmid DNA extracted. The fragments corresponding to *anc*^*HLD-RLuc*^ containing the inserted transposon (~2 kbp) were obtained by double restriction digestion (NdeI/BamHI) followed by gel extraction and ligated back into pID-Tet (50–100 ng). The ligation products were then transformed into *E. cloni* 10G cells. Upon selection on LB-agar-amp-cam, transformants (~1–2 × 10^6^ CFU) were pooled and their plasmid DNA extracted, yielding TransDel or TransIns insertion libraries depending on the TRIAD transposon used at the start. For the generation of the triplet nucleotide deletion library of Anc^HLD-RLuc^, TransDel insertion library plasmids were first digested with MlyI to remove TransDel. The fragments corresponding to linearized pID-Tet::*anc*^*HLD-RLuc*^ (with a −3 bp deletion in Anc^HLD-RLuc^) were isolated by gel electrophoresis and purified. Self-circularization was then performed using T4 DNA ligase and 10–50 ng linearized plasmid in 50 μL reaction volume (final DNA concentration: ≥1 ng/μL). Upon purification and concentration, the ligation products were transformed into electrocompetent into *E. cloni* 10G cells subsequently selected on LB-agar-amp, yielding a library of gene of interest variants with random triplet nucleotide deletions. For the generation of the triplet nucleotide insertion library of Anc^HLD-RLuc^, TransIns insertion library plasmids were first digested with NotI and MlyI to remove TransIns. The linearized pID-Tet::*anc*^*HLD-RLuc*^ plasmids were recovered by gel electrophoresis and purification. TRIAD cassette Ins1 (containing randomized 3 bp at one extremity and a kanamycin-resistance gene) was extracted from pUC57 by NotI/MlyI digestion, isolated by gel electrophoresis, purified and inserted into the linearized pID-Tet::*anc*^*HLD-RLuc*^ plasmid (50–100 ng) in a 1:3 molar ratio. After purification and concentration, these ligation products were transformed into *E. cloni* 10G cells and the transformants (~2 × 10^6^ CFU) were selected on LB-agar supplemented with 100 μg/mL ampicillin and 50 μg/mL kanamycin. The resulting plasmid ins1 insertion library was then extracted from the transforming colonies and subsequently digested with AcuI. The linearized pID-Tet::*anc*^*HLD-RLuc*^ plasmids (with a 3 bp insertion) were recovered by gel electrophoresis, purified and subsequently treated with the Klenow fragment of DNA Polymerase I to remove 3′ overhangs created by AcuI digestion. After that blunting step, the plasmids were self-circularized. The resulting ligation products were transformed into electrocompetent *E. cloni* 10G cells subsequently plated on LB-agar-amp, yielding libraries of Anc^HLD-RLuc^ variants with random triplet nucleotide insertions (one per variant). The same procedure was applied to generate a second-round triplet nucleotide insertion library of AncINS. All the libraries were purified and stored in the form of plasmid solutions prior to transformation into *E. coli* for protein variant expression and screening. Plasmid DNA from 10 randomly selected colonies from all three libraries were sequenced for quality control (Eurofins Genomics, Germany).

### Point mutagenesis of RLuc8

Site-directed PCR-based mutagenesis was applied to create *rLuc8-W121F/E144Q* in two steps using QuikChange site-directed mutagenesis kit via manufacturer’s protocol (Agilent, USA). First, *rLuc8-W121F* was created using oligonucleotides RLuc8-W121F-FWD1 and RLuc8-W121F-RVS1 with *rLuc8* gene serving as a template. Then *rLuc8-W121F* gene was used as a template to create *rLuc8-W121F/E144Q* using RLuc8-E144Q-FWD2 and RLuc8-E144Q-RVS2. Error-free clones were confirmed by DNA sequencing (Eurofins Genomics, Germany). Mutagenic primers are available in Supplementary Table 8.

### Transplantation of secondary structure elements

The Anc^HLD-RLuc^ and RLuc8 sequences were aligned using Jalview^43^ and T-coffee^44^ with default settings (Supplementary Fig. 9). The construct pET21b::*anc*^*HLD-RLuc*^ (NdeI/BamHI) was chosen as a template for mutagenesis using Phusion® High-Fidelity DNA Polymerase according to the manufacturer’s protocol (New England BioLabs, USA). Mutagenic primers (Supplementary Table 8) (Sigma-Aldrich, USA) were designed manually for two separate PCR runs (Supplementary Fig. 16). In the next step, a standard fusion PCR protocol was used to fuse DNA fragments from the first two PCR runs. The resulting fused DNA fragment was cloned into pET21b or pID-Tet vectors. The error-free status of the clones was confirmed by sequencing (Eurofins Genomics, Germany).

### Cultivation of InDel libraries in 96-well plates

Chemocompetent *Escherichia coli* BL21 cells (♯C2530H, New England BioLabs, USA) were transformed with the *anc*^*HLD-RLuc*^ insertion/deletion library (in the pID-Tet vector) and grown on LB agar plates containing 100 μg/ml ampicillin overnight at 37 °C. The cells transformed with the vector pID-Tet (lacking an insert) and pID-Tet::*anc*^*HLD-RLuc*^ were used as negative and positive controls, respectively, when screening the libraries. Single colonies of transformed cells carrying InDel variants of *anc*^*HLD-RLuc*^ were transferred into sterile 96-well plates containing 150 μl LB medium with 100 μg/ml ampicillin in each well. The plates were covered with AeraSealTM film (Sigma-Aldrich, USA) and incubated for 15 h at 37 °C under shaking at 200 rpm. After cultivation, 100 μl of the culture was transferred into new microtiter plates (MTPs) and 100 μl of fresh LB medium with 100 μg/ml ampicillin and anhydrotetracycline (to a final concentration 200 ng/ml) was added to each well. To make *replica* plates to be stored at −70 °C, 50 μl of 30% glycerol was added to the remaining 50 μl of the culture. The MTP was incubated at 30 °C and 200 rpm for 4 h. Cell cultures were harvested by centrifugation at 1600 × *g* for 20 min. The supernatant was discarded and the MTP was frozen at −70 °C. Before screening of the libraries, MTPs were defrosted and kept at laboratory temperature for 10 min. Then, 70 μl of lysis buffer (20 mM potassium phosphate, 20 mM Na_2_SO_4_ and 1 mM EDTA, pH 8.0) containing lysozyme (1 mg/ml) was added to each well. Cell debris was removed from the lysate by centrifugation at 1600 × *g* for 20 min after incubation at 23 °C and 100 rpm for 1 h. Robotic cultivation and cell lysis were performed analogously to the manual screening described above using a colony picking robot (Colony Picker, Molecular Devices QPix 420), a liquid handling robot (Bravo, Agilent) with a 96-tip head and the LARA robotic system (Greifswald, Germany).

### Screening of luciferase activity in 96-well plates

Luciferase activity was measured using a previously described procedure^14^, with adaptation, in both the manual and robotic screenings. For manual screening, 30 μl of cell lysate was transferred into a new MTP and 220 μl of assay buffer (100 mM potassium phosphate, 1 mM Na_2_SO_4_, pH 7.5) with 2.2 μM coelenterazine (CTZ) was added. The luminescence signal was immediately measured for 22 s with the gain value set to 3250. Before the addition of the assay buffer with CTZ, the sample’s baseline luminescence signal was measured for 10 s. Luminescence was measured at 30 °C in a FLUOstar OPTIMA Microplate Reader (BMG Labtech, Germany). Luciferase activity was expressed in relative light units (RLU) s^−1^ mg^−1^ of an enzyme^14^. The RLUs were integrated over the first 72.5 s immediately after injection of the substrate into the enzyme solution.

For robotic screening, 50 μl of cell lysate was pipetted by a Liquid Handling Robot into a new white MTP (Nunc™ F96 MicroWell™ White Polystyrene Plate, Nunclon Delta Surface, Thermo Fisher Scientific) and the reaction was initiated by adding 50 μl of a CTZ stock solution (1 mg of CTZ dissolved in 2 ml of pure EtOH) diluted 1000x in MilliQ water. The luminescence signal was measured using a VarioscanLUX reader (Thermo Fisher Scientific, SkanIt Software 4.1 for Microplate Readers RE, ver. 4.1.0.43). Each measurement consisted of 50 readings, each of which took 200 ms (standard optics, automatic dynamic range). The CTZ solution was dispensed during reading 15 at a moderate dispensation speed. Luciferase activity was expressed in relative light units (RLU) s^−1^ mg^−1^ of an enzyme^14^.

### Screening of dehalogenase activity in 96-well plates

Haloalkane dehalogenase activity measurements were based on a pH assay^45^ according to Holloway and co-workers^45^. The principle of the assay is based on the detection of protons produced during the dehalogenation reaction. Substrate 1-bromobutane (100 μl) was incubated in the reaction buffer (200 ml; 1 mM HEPES, 20 mM Na_2_SO_4_, 1 mM EDTA and 25 μg/ml phenol red, pH 8.2) at 37 °C for 30 min. Fifteen microlitres of cell lysate was transferred into new MTP and 185 μl of assay buffer with 1-bromobutane were added. The MTP plate was properly closed by the Adhesive film for microplates (VWR, USA) and incubated at laboratory temperature for 15 h. The change in colour of pH indicator was measured at 540 nm by using an Eon spectrometer (BioTek, USA).

### Overproduction and purification of Anc^HLD-RLuc^ variants

Anc^HLD-RLuc^ variants were overexpressed from pID-Tet (amp^R^) in *E. coli* BL21 cells (#C2530H, New England BioLabs, USA) cultivated in LB medium supplemented with ampicillin (100 μg/ml) at 37 °C. Protein production was induced under the TET promotor at 20 °C once the OD_600_ reached ~0.5 by adding anhydrotetracycline (Cayman Chemical, USA) to a final concentration of 200 ng/ml. At small scale, proteins were purified using the MagneHis^TM^ Protein Purification System (Promega, USA), dialysed using a Slide-A-Lyzer^TM^ MINI Dialysis Device (Thermo Scientific, USA), and their purity was verified by SDS-polyacrylamide gel electrophoresis. At large scale, proteins were purified by affinity chromatography targeting their C-terminal hexahistidine tags. The monomer fraction was separated on a HiLoad^TM^ 16/600 Superdex^TM^ 200 pg column (GE Healthcare, UK) equilibrated with 100 mM potassium phosphate buffer (pH 7.5). All enzymes were concentrated using Amicon^R^ Ultra-15 Ultracel^R^−10K Centrifugal Filter Units (Merck Millipore Ltd., Ireland). The purity of all enzyme preparations was checked by SDS-polyacrylamide gel electrophoresis; in all cases, only one band corresponding to the monomer fraction was visible.

### Large-scale overproduction and purification of RLuc8-W121F/E144Q

The *E. coli* BL21 (DE3) (C2527H, New England BioLabs, USA) cells were transformed by the heat shock method with the plasmid pET21b::*rLuc8-W121F/E144Q*. Ampicillin-resistant colonies were inoculated in LB medium (10 ml) (1xLB medium, ampicillin 100 μg/ml), which was incubated (200 rpm) overnight at 37 °C. On the next day, a bacterial culture was used to inoculate 5-liter Erlenmeyer flasks containing 1 liter of 1xLB medium with ampicillin (100 μg/ml) where cells were grown (200 rpm, 37 °C) until the culture reached OD_600_ = 0.5. Induction of expression was done at 22 °C by adding 0.5 ml of 1 M IPTG, and the culture was then incubated overnight (typically 12–16 h) at 22 °C/115 rpm. Next day, the bacterial biomass was harvested by centrifugation (3000 × *g*/10 min/4 °C) and re-suspended in a purification buffer (500 mM NaCl, 20 mM potassium phosphate buffer pH 7.5, 10 mM imidazole) and lysed by sonication (50% amplitude, 32 min (5 s pulse/5 s pause) using a sonicator Sonic Dismembrator Model 705 (Fisher Scientific, USA). The sonicated lysate was clarified by centrifugation (21,000 *×* *g*, 60 min, 4 °C). The supernatant was then collected, filtered and applied on Ni-NTA Superflow Cartridge (Qiagen, Germany) column equilibrated with the purification buffer. The target enzyme was eluted by a linear gradient of the purification buffer supplemented with 300 mM imidazole. The eluted protein fractions of RLuc8-W121F/E144Q were pooled and dialysed overnight against 100 mM NaCl, 10 mM Tris-HCl pH = 7.0. The dialysed protein was subsequently loaded onto 16/60 Superdex 200 gel filtration column (GE Healthcare, UK) pre-equilibrated with the corresponding buffer. The purified RLuc8-W121F/E144Q protein was concentrated with an Amicon Ultra centrifugal filter units (Millipore, USA), and protein concentrations were assayed by the DS-11 Spectrophotometer (DeNovix, USA).

### Mammalian cells experiments

NIH/3T3 mouse fibroblast cells (ATCC^®^ CRL-1658™) were transfected according to manufacturer’s protocol using Lipofectamine 2000 (Thermo Fisher, USA) with pcDNA3.1(+) plasmids containing genes codon-optimized for expression in mammalian cells (Gene Art, Thermo Fisher, USA). Cells were lysed 24 h after transfection and luciferase activity was measured in lysate via Microplate Reader FLUOstar Omega (BMG Labtech, Germany) using a commercial *Renilla* Luciferase Assay System (Promega, USA) and also using an in-house prepared assay buffer 100 mM PBS pH = 7.5 with 4.5 μM CTZ (final concentration in the reaction mixture). Cells transfected with pcDNA3.1(+) plasmid were used as a negative control. The measurements were done in triplicates.

### Circular dichroism spectroscopy

Circular dichroism (CD) spectra were recorded at 20 °C using a Chirascan spectropolarimeter (Applied Photophysics, UK). Data were collected from scans of the focal proteins from 185 to 260 nm at 100 nm/min with a 1 s response time and 1 nm bandwidth in 0.1 cm quartz cuvettes. Each presented spectrum (Fig. S13) is an average of five individual scans, corrected for absorbance of the buffer. The CD data were expressed in terms of mean residue ellipticity Θ_MRE_ described by Eq. (1).

1 $${\Theta }_{{\rm{MRE}}}=\frac{{\Theta }_{{\rm{obs}}}.{M}_{{\rm{W}}}.100}{n.c.l}$$

where Θ_obs_ is the observed ellipticity in degrees, *M*_w_ is the scanned protein’s molecular weight, *n* is the number of residues, *l* is the cell path length, *c* is the protein concentration (in mg/ml) and the factor of 100 originates from the conversion of the molecular weight to mg/dmol.

### Thermal stability

Thermal unfolding was studied by using a NanoDSF Prometheus instrument (NanoTemper, Germany) to monitor Trp fluorescence during heating at 1 °C/min from 20 to 90 °C. The melting temperatures (*T*_onset_ and *T*_m_) were evaluated directly by ThermControl v2.0.2.

### Microfluidic determination of temperature profiles and thermodynamics

Temperature profiles of specific activities of individual enzyme variants towards the substrate 1,3-dibromopropane were measured in 2.5 °C increments from 20 to 40 °C using a capillary-based droplet microfluidic platform, enabling characterization of enzymatic activity within droplets for multiple enzymes in a single run^46^. The droplets were generated using the Mitos Dropix (Dolomite, UK). A custom sequence of droplets (150 nl aqueous phase, 300 nl oil spacing) was generated using negative pressure (microfluidic pump) and the droplets were guided through polyethylene tubing to the incubation chamber. Within the incubation chamber, the halogenated substrate was delivered to the droplets via a combination of microdialysis and partitioning between the oil (FC 40) and the aqueous phase. The reaction solution consisted of a weak buffer (1 mM HEPES, 20 mM Na_2_SO_4_, pH 8.2) and a complementary fluorescent indicator 8-hydroxypyrene-1,3,6-trisulfonic acid (50 μM HPTS). The fluorescence signal was obtained by using an optical setup with excitation laser (450 nm), a dichroic mirror with a cut-off at 490 nm filtering the excitation light and a Si-detector. By employing a pH-based fluorescence assay, small changes in the pH were observed and enabling monitoring of the enzymatic activity. The reaction progress was analysed as an end-point measurement recorded after passing of 7 or 10 droplets/sample through the incubation chamber. The reaction time was 4 min. The raw signal was processed by a droplet detection script^46^ written in MATLAB 2017b (Mathworks, USA) to obtain the specific activities. Natural logarithms of the specific activities were used in subsequent thermodynamic analysis to generate both Arrhenius and Eyring plots and to derive thermodynamic parameters (Supplementary Fig. 1).

### Anisotropic network modelling

Secondary structure elements were defined in Anc^HLD-RLuc^ and RLuc8 based on their respective crystal structures (PDB ID 6G75, chain A; and 2PSF, chain B) using DSSP^47^ followed by manual edition after visual inspection (Supplementary Tables 2, 3). Anisotropic network models were computed using the Prody 1.10.8 standalone package^48^ and the position-specific vector of squared fluctuations and matrix of motion cross-correlations were obtained. These matrices were further extended by calculating the averaged cross-correlation values corresponding to each secondary structure element. To facilitate distinction of differences in predicted motions, the matrix calculated for RLuc8 was subtracted from that calculated for Anc^HLD-RLuc^. The final matrix *M* encompassed values ranging from −2 (motions more cross-correlated in RLuc8) to 2 (motions more cross-correlated in Anc^HLD-RLuc^), where values close to 0 indicate similar motions in the two structures. To improve understanding of effects of mutations on the 25 selected variants, the positions and secondary structure elements of mutations resulting in extreme values were mapped to both reference crystals (Anc^HLD-RLuc^ and RLuc8) based on their alignment (Supplementary Fig. 9). Then, three groups of interesting regions were defined and used to annotate each variant with 18 values obtained from matrix *M*: nine for the cross-correlation of motions of the specific position and nine for the secondary structure element.

### Calculation of segment-centred cross-correlation values from ANM

Let *i*_1_ and *i*_2_ be the indices of the beginning and ending position of a given secondary structure element (SSE_*i*_) in the structure for which the ANM is computed; *j*_1_ and *j*_2_ be the indices of the beginning and ending position of a different secondary structure element (SSE_*j*_) on the same structure; *var*(*x*) and *var*(*y*) be the squared fluctuation value for the *x*th and *y*th residue on the structure, respectively; and *r*(*x,y*) the cross-correlation value for the pair formed by the *x*th and *y*th residues on the structure obtained from the same ANM calculation. The averaged cross-correlation value for the pair (SSE_*i*_, SSE_*j*_) can be calculated according to Eq. (2).

2 $${Ccor}\left({{{\mathrm{SSE}}}}_{i},{{{\mathrm{SSE}}}}_{j}\right)\frac{\mathop{\sum }\nolimits_{x={i}_{1}}^{x={i}_{2}}\mathop{\sum }\nolimits_{y={j}_{1}}^{y={j}_{2}}\left(r\left(x,y\right)\sqrt{{var}\left(x\right)\cdot {var}\left(y\right)}\right)}{\sqrt{\mathop{\sum }\nolimits_{x={i}_{1}}^{x={i}_{2}}\mathop{\sum }\nolimits_{y={i}_{1}}^{y={i}_{2}}\left(r\left(x,y\right)\sqrt{{var}\left(x\right)\cdot {var}\left(y\right)}\right).\mathop{\sum }\nolimits_{x={j}_{1}}^{x={j}_{2}}\mathop{\sum }\nolimits_{y={j}_{1}}^{y={j}_{2}}r\left(x,y\right)\sqrt{{var}\left(x\right)\cdot {var}\left(y\right)}}}$$

Note that if additionally to the *E* SSE_*x*_ elements defined in each of the Supplementary Tables S2, S3, each of the *P* amino-acids of each analysed protein is considered as one of such elements, the dimension of the resulting cross-correlation matrix is (*E* + *P*)^2^. Such matrix would be formed by two diagonal matrices with dimensions *E*^2^ and *P*^2^ representing the SSE to SSE and amino-acid to amino-acid cross-correlations, respectively; and by two mirroring rectangular matrices (*E* × *P* and *P* × *E*) representing the amino-acid to SSE cross-correlations.

### Multivariate statistical analysis

Partial least squares (PLS) regression analysis^49^ was used to explore relationships between structural and molecular variables (X) of the proteins and their enzymatic activities (Y), all of which were autoscaled and centred. The complete data matrix used in the PLS analysis is provided in Source data file. Variable importance in the projection (VIP)^50^ and variable weight plots^51^ were used to assess the importance of every descriptor in the model, with further validation by cross-validation^49^ and permutation testing. During the cross-validation procedure, parts of the Y data are not considered during model building, and the ability of the resulting model to predict those data is assessed by comparing predicted and actual values, providing cross-validated *Q*^2^ values. In this study, 1/7 of the mutants were deleted in each cross-validation round. In the permutation testing, the model was recalculated 999 times with a randomly re-ordered dependent variable. SIMCA-P version 12 (Umetrics, Umea, Sweden) was used for statistical analyses.

### Success rate frequency analysis

Luminescence readings were processed to determine each tested variant’s luciferase activity relative to the mutagenesis template. Each microtiter plate used for this analysis included negative and positive controls (in sets of four wells) as well as the tested variants, and transformations were applied to obtain relative activities of the preparation in each well. First, the negative control wells were averaged time-point by time-point. Second, the rest of the wells were blanked using subtracting from each of their time-point values of the corresponding averaged time-point from the negative controls. Third, two segments for each data series were defined: pre- and post-injection of the coelenterazine substrate, which occurred ten seconds after the beginning of the readings on the FLUOstar OPTIMA instrument. The pre-injection series consisted of all data points before injection time. The post-injection series consisted of all data points collected from the moment the reading of the signal was stable (0.2 s after the injection time) until the end of the readings. From each data series, outliers defined as *Q*1 − 1.5*(*Q*3 − *Q*1) and *Q*3 + 1.5*(*Q*3 − *Q*1) (where *Q*1 and *Q*3 represent the first and third quartile values of each distribution) were omitted to remove noise from the data. Fourth, the average value of the pre-injection series was subtracted from the average value of the post-injection one, to obtain the raw intensity value of each well. Fifth, the reference intensity value of the plate was calculated by averaging the raw intensity values of the positive control wells. Sixth, the relative intensity value was calculated as the ratio of the raw intensity value of each well over the reference intensity value of the plate.

### Steady-state kinetics measurement and data analysis

Solid coelenterazine was dissolved in ice-cold ethanol and stored under nitrogen atmosphere in dark glass vials at –20 °C. Before measurement, concentration and quality of the ethanol stock solution were verified spectrophotometrically. Series of buffer solutions with different coelenterazine concentration was prepared by manual injection of an appropriate volume of the ethanol stock solution into 10 ml of 100 mM phosphate buffer pH 7.5 immediately before the measurement. The reaction mixture was composed of 10% (v/v) enzyme solution in 100 mM phosphate buffer pH 7.5 and 90% (v/v) of buffer solution of coelenterazine. All reactions were carried out at 37 °C in microtiter plates using the microplate reader FLUOstar OPTIMA (BMG Labtech, Germany) set to broad-spectrum luminescence reading. Microplate well with pre-pipetted 25 μl of enzyme solution was first monitored for background light for 10 s, after which, 225 μl of a buffer solution with coelenterazine was added via an automatic syringe. The luminescence of the reaction mixture was then measured for the desired time until the luminescence intensity decreased under 0.5% of its maximal measured value. Each reaction was performed in three repetitions. The gain of the reader was tailored to each enzyme separately, however, for each enzyme, all readings at different substrate concentrations were obtained using the same gain value.

An updated protocol applying new standards for collecting and fitting steady-state kinetic data^52,53^ was used. Unlike the classical initial velocity analysis, which requires a sophisticated luminometer calibration and quantum yield evaluation to obtain complete kinetic data^54^, luminescence data were recorded when letting the reaction proceed to completion beyond the initial linear phase. The recorded luminescence traces (rate vs. time) were transformed to reaction progress curves corresponding to cumulative luminescence in time (Supplementary Fig. 5). The transformed steady-state kinetic data (product vs. time) were fitted globally by numerical methods with the KinTek Explorer (KinTek Corporation, USA) to obtain direct estimates of turnover number *k*_cat_, Michaelis constant *K*_m_, specificity constant *k*_cat_/*K*_m_, and equilibrium dissociation constant for enzyme-product complex *K*_p_ with no need for luminometer quantum yield calibration. The software allows for the input of a given kinetic model via a simple text description, and the programme then derives the differential equations needed for numerical integration automatically. Numerical integration of rate equations searching a set of kinetic parameters that produce a minimum *χ*^2^ value was performed using the Bulirsch–Stoer algorithm with adaptive step size, and nonlinear regression to fit data was based on the Levenberg–Marquardt method^55^. To account for fluctuations in experimental data, enzyme or substrate concentrations were slightly adjusted (±5%) to derive best fits. Residuals were normalized by sigma value for each data point. The standard error (S.E.) was calculated from the covariance matrix during nonlinear regression. In addition to S.E. values, more rigorous analysis of the variation of the kinetic parameters was accomplished by confidence contour analysis by using FitSpace Explorer (KinTek Corporation, USA). In these analyses, the lower and upper limits for each parameter were derived (Supplementary Table 4) from the confidence contour obtained from setting *χ*^2^ threshold at 0.9^56^. The scaling factor, relating luminescence signal to product concentration, was applied as one of the fitted parameters, well constrained by end-point levels of kinetic traces recorded at particular substrate concentrations. Depletion of the available substrate after the reaction was ensured by repeated injection of the fresh enzyme. The steady-state model (Eqs. (3)–(6)) was used to obtain the values of turnover number *k*_cat_, Michaelis constant *K*_m_ and equilibrium dissociation constant for enzyme-product complex *K*_p_. A conservative estimate for diffusion-limited substrate and product binding *k*_+1_ and *k*_−3_ (100 μM^−1^.s^−1^) was used as a fixed value to mimic rapid equilibrium assumption. An alternative form of the model (Eqs. (7) and (8)) was used to obtain estimates of *k*_cat_/*K*_m_ directly by setting *k*_−1_ = 0^52,53^.

3 $${\rm{E}}+{\rm{S}}\begin{array}{c}\mathop{\to }\limits^{100}\\ \mathop{\leftarrow }\limits_{{k}_{-1}}\end{array}{\rm{E}}.{\rm{S}}\mathop{\to }\limits^{{k}_{+2}}{\rm{E}}.{\rm{P}}\begin{array}{c}\mathop{\to }\limits^{{k}_{+3}}\\ \mathop{\leftarrow }\limits_{100}\end{array}{\rm{E}}+{\rm{P}}$$

4 $${k}_{{\rm{cat}}}={k}_{+2}$$

5 $${K}_{{\rm{m}}}={k}_{-1}/100$$

6 $${K}_{{\rm{p}}}={k}_{+3}/100$$

7 $${\rm{E}}+{\rm{S}}\mathop{\to }\limits^{{k}_{+1}}{\rm{E}}.{\rm{S}}\mathop{\to }\limits^{{k}_{+2}}{\rm{E}}.{\rm{P}}\begin{array}{c}\mathop{\to }\limits^{{k}_{+3}}\\ \mathop{\leftarrow }\limits_{100}\end{array}{\rm{E}}+{\rm{P}}$$

8 $${k}_{{\rm{cat}}}/{K}_{{\rm{m}}}={k}_{+1}$$

### Bioluminescence decay kinetics

Bioluminescence decay kinetics were monitored in the same conditions as the steady-state kinetics, with final concentrations of 2.2 μM CTZ and 50 nM enzyme. The obtained luminescence kinetic data were fitted to an exponential decay or logistic model using Origin 6.1 (OriginLab, USA) to obtain the half-life *t*_1/2_ of the luminescence signal.

### Transient kinetics of substrate binding experiments and data analysis

Transient kinetic traces of the protein variants were monitored after rapidly mixing components in 100 mM potassium phosphate buffer (pH 7.5) using the Stopped-Flow SFM 3000 mixing system and the MOS-500 spectrometer (BioLogic, France). In each case, the reaction was initiated by mixing 75 µl of an enzyme solution with 75 µl of CTZ solution and then monitored by the changes of fluorescence at 340 ± 13 nm after excitation at 295 nm. In experiments with each protein variant, concentrations of the CTZ substrate and an enzyme were separately varied in sets of seven consecutive replicates and the resulting kinetic traces were averaged. The experiments were based on monitoring changes in native tryptophan fluorescence that was quenched by the coelenterazine substrate upon its binding by an enzyme. The drop of the initial fluorescence level caused by the non-specific interactions and inner filter effects of coelenterazine was corrected based on experiments with bovine serum albumin, which exhibited the same initial decrease of the fluorescence signal. At room temperatures, the kinetics of the substrate binding was too fast, and most of the kinetic information was lost in the dead time of the instrument (0.3 ms). After decreasing the temperature down to 15 °C, kinetic traces collected for AncINS, AncFT and RLuc8 exhibited a triple-exponential decay of the fluorescence signal.

The two initial kinetic phases related to the binding of the substrate were fit with a double exponential equation (Eq. (9)) for each fluorescence trace (Supplementary Fig. 8). The dependence of the observed rates on the substrate concentration for both the fast and the slow kinetic phases were fit according to the induced-fit (IF) mechanism (Eqs. (10)–(12)) and the conformational selection (CS) mechanism (Eqs. (13)–(15)), assuming that *k*_−1_ >> *k*_−2_, *k*_+2_^57^. The obtained values were used as initial estimates of the elementary rate constants describing the binding process.

9 $$F={F}_{{\rm{SS}}}+{A}_{1}\cdot {e}^{-{k}_{{\rm{fast}}}\cdot t}+{A}_{2}\cdot {e}^{-{k}_{{\rm{slow}}}\cdot t}$$

10 $${\rm{E}}+{\rm{S}}\begin{array}{c}\mathop{\to }\limits^{{k}_{+1}}\\ \mathop{\leftarrow }\limits_{{k}_{-1}}\end{array}{\rm{E}}.{\rm{S}}\begin{array}{c}\mathop{\to }\limits^{{k}_{+2}}\\ \mathop{\leftarrow }\limits_{{k}_{-2}}\end{array}{{\rm{E}}}^{\ast }.{\rm{S}}$$

11 $${k}_{{\rm{fast}},{\rm{IF}}}={k}_{+1}\cdot \left[S\right]+{k}_{-1}$$

12 $${k}_{{\rm{slow}},{\rm{IF}}}=\frac{{k}_{+2}\cdot \frac{{k}_{+1}}{{k}_{-1}}\cdot [S]}{\frac{{k}_{+1}}{{k}_{-1}}\cdot \left[S\right]+1}+{k}_{-2}$$

13 $${\rm{E}}+{\rm{S}}\begin{array}{c}\mathop{\to }\limits^{{k}_{+1}}\\ \mathop{\leftarrow }\limits_{{k}_{-1}}\end{array}{{\rm{E}}}^{\ast }+{\rm{S}}\begin{array}{c}\mathop{\to }\limits^{{k}_{+2}}\\ \mathop{\leftarrow }\limits_{{k}_{-2}}\end{array}{{\rm{E}}}^{\ast }.{\rm{S}}$$

14 $${k}_{{\rm{fast}},{\rm{CS}}}={k}_{+2}\cdot \left[S\right]+{k}_{-1}$$

15 $${k}_{{\rm{slow}},{\rm{CS}}}=\frac{{k}_{+1}\cdot \left(\frac{{k}_{+1}}{{k}_{-1}}\cdot {k}_{+2}\cdot \left[S\right]+{k}_{-2}\right)}{{k}_{+1}\cdot \frac{{k}_{+1}}{{k}_{-1}}\cdot {k}_{+2}\cdot \left[S\right]+{k}_{-2}}$$

In the case of Anc^HLD-RLuc^, the kinetic traces exhibited slow equilibration with low signal amplitudes, resulting in a limited precision of analysis (Supplementary Fig. 8). The data could be fit with only a single exponential equation (Eq. 16), and the dependence of the observed rate constant and the amplitude on the substrate concentration were fit according to a one-step binding mechanism (Eqs. (17)–(19)). Such a result pointed out that the rigidified ancestral enzyme shows no profound conformational flexibility and that only a simple substrate binding could be detected.

16 $$F={F}_{{\rm{SS}}}+A\cdot {e}^{-{k}_{{\rm{obs}}}\cdot t}$$

17 $${\rm{E}}+{\rm{S}}\begin{array}{c}\mathop{\to }\limits^{{k}_{+1}}\\ \mathop{\leftarrow }\limits_{{k}_{-1}}\end{array}{\rm{E}}.{\rm{S}}$$

18 $${k}_{{\rm{obs}}}={k}_{+1}\cdot \left[S\right]+{k}_{-1}$$

19 $$A=\frac{{A}_{{\rm{lim}}}\cdot [S]}{{K}_{{\rm{d}}}+\left[S\right]}$$

The original raw kinetic data with residuals normalized by sigma values were subsequently analysed globally by numerical simulation using the KinTek Explorer software (KinTek Corporation, USA)^52,55,56^ and following the same protocol as described in the steady-state kinetics section. The time-course of the fluorescence quenching upon substrate binding was described with Eqs. (20)–(22) for the induced-fit, conformational-selection, and one-step binding mechanisms, respectively. The estimates of the rate constants obtained by analytical fitting were used as starting points, and the final values were obtained by fitting the mechanism directly to the collected kinetic data. The quality of the best fits for different mechanisms was compared in terms of *χ*^2^ value, “chi-by-eye” assessment, and confidence contour analysis assessing constraint and independency of the determined parameters. Based on this analysis, the correct mechanism of the substrate binding and the respective elementary rate constants were identified. Lower and upper limits for each parameter were derived for the *χ*^2^ threshold of 0.95^56^.

20 $${F}_{{\rm{IF}}}=f\cdot \left([E]+a\cdot [{ES}]+b\cdot [{E}^{\ast }S]\right)$$

21 $${F}_{{\rm{CS}}}=f\cdot \left([E]+a\cdot [{E}^{\ast }]+b\cdot [{E}^{\ast }S]\right)$$

22 $${F}_{{\rm{one}}-{\rm{step}}}=f\cdot \left([E]+a\cdot [{ES}]\right)$$

### Hydrogen/deuterium exchange (HDX) mass spectrometry

Enzyme samples were diluted with 100 mM phosphate buffer in H_2_O (pH 7.5) to prepare undeuterated controls and for peptide mapping, or with 100 mM phosphate buffer in D_2_O (pD 7.1) to prepare deuterated samples. The final concentrations of the enzyme samples used for analysis was 2 µM. HDX was carried out at room temperature and was quenched after 10, 60 or 300 s by adding 1 M HCl in 1 M glycine with pepsin. Each sample was directly injected into an LC-system (UltiMate 3000 RSLCnano, Thermo Fisher Scientific, USA) with an immobilized nepenthesin enzymatic column (Affipro, CZ; 15 µl bed volume, flow rate 20 µl/min, 2% acetonitrile/0.05% trifluoroacetic acid). Peptides were trapped and desalted on-line on a peptide microtrap (Michrom Bioresources, CA) for 3 min at a flow rate of 20 µl/min. Next, the peptides were eluted onto an analytical column (Jupiter C18, 1.0 × 50 mm, 5 µm, 300 Å, Phenomenex, CA) and separated by linear gradient elution starting with 10% buffer A in buffer B and rising to 40% buffer A over 2 min. This was followed by 31 min isocratic elution at 40% B. Buffers A and B consisted of 0.1% formic acid in water and 80% acetonitrile/0.08% formic acid, respectively. The immobilized nepenthesin column, trap cartridge, and analytical column were kept at 1 °C using a refrigerated system (Science Instruments and Software, CZ).

Mass spectrometric analysis was carried out using an Orbitrap Elite mass spectrometer (Thermo Fisher Scientific, USA) with ESI ionization connected on-line to a robotic system based on the HTS-XT platform (CTC Analytics, Switzerland). The instrument was operated in a data-dependent mode for peptide mapping (HPLC-MS/MS). Each MS scan was followed by MS/MS scans of the three most intensive ions from both CID and HCD fragmentation spectra. Tandem mass spectra were searched using SequestHT against the cRap protein database (ftp://ftp.thegpm.org/fasta/cRAP) containing the sequences of Rluc8, Anc^HLD-RLuc^ and the studied mutants with the following search settings: mass tolerance for precursor ions of 10 ppm, mass tolerance for fragment ions of 0.6 Da, no enzyme specificity, two maximum missed cleavage sites and no-fixed or variable modifications. The false discovery rate at the peptide identification level was set to 1%. Sequence coverage was analysed with Proteome Discoverer version 1.4 (Thermo Fisher Scientific, USA) and graphically visualized with the MS Tools application^58^. Analysis of deuterated samples was done in HPLC-LC-MS mode with ion detection in the orbital ion trap. The MS raw files together with the list of peptides (peptide pool) identified with high confidence characterized by requested parameters (amino acid sequence of each peptide, its retention time, XCorr, and ion charge) were processed using HDExaminer version 2.2 (Sierra Analytics, CA). The software used to analyse protein and peptide behaviour, creates the uptake plots that measured peptide deuteration over time with the calculated confidence level.

### HDX mass spectrometry data analysis

The acquired data were analysed as follows. The raw MS files together with the list of peptides (peptide pool) identified with high confidence characterized by requested parameters (retention time, XCorr, and charge) were processed using HDExaminer version 2.2 (Sierra Analytics, Modesto, CA). The software analysed the proteins’ and peptides’ behaviour and generated uptake data that were mapped to the proteins’ amino acid sequences via the following procedure. Each residue was assigned the uptake data from any peptide solved with high confidence. Medium confidence peptides were also accepted for positions without previously assigned data. Low confidence peptides were rejected. The final uptake value (expressed as % of deuteration) assigned to each amino acid corresponded to the average of all assigned values for its position.

### MD simulations

MD simulations were carried out on the crystal structures of RLuc8 (PDB ID 2PSF^17^), Anc^HLD-RLuc^ (PDB ID 6G75^14^), AncINS (PDB ID 6S6E) and AncFT (PDB ID 6S97). Hydrogen atoms were added to the structures using the H++ web server^59^, at pH 7.5. Water molecules from the crystal structures, which did not overlap with the protonated structures, were retained. The systems were solvated using the solvate module of high-throughput molecular dynamics (HTMD) in a cubical water box of TIP3P water molecules so that all atoms were at least 10 Å from the surface of the box^60^. Cl^−^ and Na^+^ ions were added to neutralize the protein’s charge and get a final concentration of 0.1 M. In all the simulations periodic boundary conditions were applied, the particle mesh Ewald method was used to treat interactions beyond a 9 Å cut-off, electrostatic interactions were suppressed >4 bond terms away from each other, and the smoothing and switching of van der Waals and electrostatic interactions were cut-off at 7.5 Å^61^. HTMD was used for adaptive sampling of the RMSD of the Cα atoms of residues 10–290. The production runs (20 ns) were started with the files resulting from the equilibration using settings from the final equilibration step. The trajectories were saved every 100 ps. The adaptive epochs were updated every 6 h to run with a minimum of five simulations and a maximum of 10 simulations for the total maximum of 110 epochs using TICA in 1 dimension^62^.

### Equilibration of systems for MD simulations

The systems were equilibrated using the Equilibration_v2 module of HTMD 1.23.4^59^. The system was first minimized using the conjugate-gradient method for 500 steps, after which the system was heated and minimized as follows (i) 500 steps (2 ps) of NVT heating, with the Berendsen barostat, to 310 K, with constraints on all heavy atoms of the protein; (ii) 2.5 ns of NPT equilibration, with the Langevin thermostat, with 1 kcal mol^−1^ Å^−2^ constraints on all the heavy atoms of the protein; and (iii) 2.5 ns of NPT equilibration, with the Langevin thermostat, without constraints. During the equilibration simulations, holonomic constraints were applied to all hydrogen-heavy atom bond terms and the mass of hydrogen atoms was scaled by a factor of 4, enabling the use of a 4 fs timestep^60,61^. This approach transfers some mass from the heavy atom to the hydrogen to slow the fastest vibrations in the hydrogen atom, which is acceptable because the thermodynamic properties of biological systems are insensitive to the distribution of atomic masses^63,64^.

### Adaptive sampling

Adaptive sampling here refers to the MD method that performs simulations in small sequential batches of 10 simulations. Before the next batch of simulations is submitted, the first batch is analysed considering a certain parameter (RMSD in this study) and the less sampled conformational regions are used to start a new batch. This way the method ensures that the sampled regions are maximized. This sampling analysis is done using Markov state models making a list of metastable states distributed through the conformational space.

### Calculation of B-factors

B-factors were calculated from all snapshots obtained from the MD simulations for the backbone atoms of each enzyme. The Metric Fluctuation tool from the HTMD package was used to calculate the RMSF, from which B-factors were calculated using Eq. (23).

23 $$B=\left[\frac{8* {\pi }^{2}}{3}\right]* {{{\mathrm{RMSF}}}}^{2}$$

The B-factors obtained from the MD simulations were standardized for each enzyme to enable comparisons between enzymes. This was done using Eq. (24).

24 $$Z=\frac{X-\mu }{\delta }$$

Here, *Z* is the standardized value, *X* is the original B-factor value, *μ* is the average of the B-factors, and *δ* is the sample standard deviation. The simulations were made into a simulation list using HTMD, water was filtered out, and crashed simulations with lengths below 20 ns were omitted^59^. The total simulation time required for the calculations was in excess of 4 μs for each enzyme. The dynamics of the enzymes were evaluated based on the RMSD of the Cα atoms of residues 10–290 to avoid problems due to the misleadingly high RMSD values of the dynamically free residues at each end of the protein. The data were clustered into 200 clusters using the MiniBatchKmeans algorithm. The implied timescale plot (based on a Markov state model with various lag times) was constructed to select a lag time for Markov model construction, using the RMSD of the full protein as a metric. Because the timescales mostly stabilized after 15 ns lag time, this value was used in the models to construct the 2 Markov states (open and closed). The final data were calculated from 1000 bootstrapping runs using 50% of the data.

### Tunnel calculations

The tunnels were calculated with Caver 3.02^23^ using 1000 random frames belonging to each state using Caver Analyst 2.0.31^65^, with Probe radius: 0.9 Å, clustering threshold: 5, shell depth: 5 Å and shell radius: 5 Å. For the Anc^HLD-RLuc^ the starting point of the tunnel was defined by: ND2 of the Asn51 and OE1 of Glu142. For other enzymes, the starting point was defined by ND2 and OE1 in analogous residues.

### Cavities calculations

The cavity calculations for the four proteins were carried out in the duplicates using the software Caver Analyst 2.0.31^65^. We have used 100 random snapshots to obtain the cavity volumes from the open conformations and 100 random snapshots from the closed conformations. A probe of 1.4 Å was used for the cavities and a maximum probe size of 3.0 Å was used to delimit the interface between solvent and cavity. The same parameters for the probes were used when calculating the cavity volume of the crystal structures. When two chains were present, the cavities were calculated for both chains. Cavities were also calculated for the minimized crystal structures, both chains when present.

### Crystallizations

Diffraction-quality crystals of AncINS were obtained using the sitting-drop vapour diffusion technique in a 96-well crystallization plate (UVXPO 2 Lens Crystallization Plate, SWISSCI, Switzerland) at 20 °C after 5 days by mixing equal volumes of AncINS (4.5 or 10.5 mg/ml) with the reservoir solution from Morpheus crystallization screen condition C5, which consists of 0.09 M NPS additive mix (sodium nitrate, sodium phosphate dibasic, ammonium sulfate) and 0.1 M buffer system 2 (sodium HEPES, MOPS) at pH 7.5 with 30% v/v precipitant mix 1 (PEG 500 MME, PEG 20,000). The crystals were fished out and directly flash-frozen in liquid nitrogen for diffraction analysis at ESRF beamline ID23-1 in Grenoble (France).

AncFT crystals were obtained using the hanging-drop vapour diffusion technique in EasyXtal 15-well plates (Qiagen, Germany) with seeding at 20 °C after 2 days by mixing 0.5 µl of seed, 2 µl of AncFT (9.2 mg/ml), and 1 µl of reservoir solution (0.2 M sodium acetate, 0.1 M Tris pH 8.5, 19% PEG 3350). Crystals were cryo-protected with 20% glycerol and flash-cooled in liquid nitrogen. Crystallographic data were collected at cryogenic temperature at SLS beamline PXIII in Villigen (Switzerland), with wavelength of 1 Å.

Prior to the co-crystallization screening, the purified protein RLuc8-W121F/E144Q was concentrated to 6.0 or 8.5 mg/ml and mixed with 3–5 molar excess of coelenterazine (stock solution of coelenterazine was 11.8 mM in isopropanol). The mixture was incubated 1 h at 4 °C, and then precipitated material was removed by centrifugation (13,000 rpm/10 min/4 °C), and the supernatant containing enzyme-ligand complexes was directly crystallized. The crystallization was performed in Easy-Xtal 15-well crystallization plates in a hanging drop vapour diffusion technique, where enzyme-ligand drops (typically 1.5 μl) were mixed with the reservoir solution (2.2 M NaHPO_4_/K_2_HPO_4_, 0.1 M sodium acetate pH = 4.5) in the ratio 1:1 and equilibrated against 500 μl of the reservoir solution. Diffraction quality crystals of RLuc8-W121F/E144Q were obtained at 20 °C after 3–5 days. All crystals used for X-ray diffraction analysis were flash-frozen in liquid nitrogen in the corresponding reservoir solutions supplemented with 20–25% glycerol. All data obtained in this project were collected at 100 K on SLS beamline PX3 (Villigen, Switzerland).

### Structure determination, model building and refinement

The crystallographic data were processed and scaled using XDS^66^ and Aimless^67^. Structures of AncINS and AncFT were solved by molecular replacement using Anc^HLD-RLuc^ (PDB ID 6G75^14^) as a search model with the help of Phaser^68^ as implemented in the Phenix package^69^. The structure of RLuc8-W121F/E144Q was solved by molecular replacement with Phenix2 using the RLuc8 structure (PDB ID 2PSD) as a search model. Multiple cycles of automated refinement were performed in the phenix.refine programme^69^ and manual model building was performed in Coot^70^. The final models were validated using tools provided in Coot^70^ and Molprobity^71^. Ramachandran favoured 95.3%, 95.9% and 96.6%, Ramachandran allowed 4.4%, 3.8% and 3.4%, Ramachandran outliers 0.3%, 0.3% and 0% for AncINS, AncFT and RLuc8-W121F/E144Q, respectively. Structural data were graphically visualized with PyMol Molecular Graphics System, version 1.8 (Schrödinger, LLC). Atomic coordinates and structure factors for AncINS, AncFT and RLuc8-W121F/E144Q were deposited in the Protein Data Bank under the codes 6S6E, 6S97, and 6YN2, respectively.

### Reporting summary

Further information on research design is available in the Nature Research Reporting Summary linked to this article.

## Supplementary information

Supplementary Information

Reporting Summary

## Data Availability

Atomic coordinates and experimental data have been deposited in the Protein Data Bank (www.wwpdb.org). They are publicly available for accession codes 6S6E and 6S97; 6YN2 will be released upon article publication. The validation reports are provided in Supplementary Notes 7, 8 and 9. The primary data from microfluidics are available in figshare with the identifier 10.6084/m9.figshare.14453700.v1. Source data are provided with this paper.

## Source data

Source Data
