# Supplementary material for: Engineering the protein dynamics of an ancestral luciferase
Source: Nat Commun. 2021 Jun 14;12:3616. doi: 10.1038/s41467-021-23450-z (PMC8203615; doi:10.1038/s41467-021-23450-z)
Supplement: Supplementary file 1 — Supplementary Information [file 41467_2021_23450_MOESM1_ESM.pdf]

SUPPLEMENTARY INFORMATION

Engineering Protein Dynamics of Ancestral Luciferase

Andrea Schenkmayerova[1,2], Gaspar P. Pinto[1,2], Martin Toul[1,2], Martin Marek[2], Lenka Hernychova[3], Joan Planas-Iglesias[1,2], Veronika Liskova[1,2], Daniel Pluskal[2], Michal Vasina[1,2], Stephane Emond[4], Mark Dörr[5], Radka Chaloupkova[2], David Bednar[1,2], Zbynek Prokop[1,2], Florian Hollfelder[4*], Uwe T Bornscheuer[5*], Jiri Damborsky[1,2*]

1. International Clinical Research Center, St. Anne's University Hospital Brno, Pekarska 53, 656 91 Brno, Czech Republic

2. Loschmidt Laboratories, Department of Experimental Biology and RECETOX, Faculty of Science, Masaryk University, Kamenice 5, Bld. A13, 625 00 Brno, Czech Republic

3. Regional Centre for Applied Molecular Oncology, Masaryk Memorial Cancer Institute, Zluty kopec 7, 656 53 Brno, Czech Republic

4. Department of Biochemistry, University of Cambridge, 80 Tennis Court Road, Cambridge CB2 1GA, UK

5. Department of Biotechnology and Enzyme Catalysis, Institute of Biochemistry, University of Greifswald, Felix-Hausdorff-Str. 4, 17487 Greifswald, Germany

These authors contributed equally: Andrea Schenkmayerova, Gaspar P. Pinto, Martin Toul, Martin Marek

[*] emails: fh111@cam.ac.uk; uwe.bornscheuer@uni-greifswald.de; jiri@chemi.muni.cz

Supplementary Note 1. Screening of InDel libraries for haloalkane dehalogenase (HLD) and luciferase (LUC) activity. Libraries of $Anc^{HLD\text{-}RLuc}$ with random single amino acid InDels distributed over the protein's length were constructed using transposon-based mutagenesis (TRIAD)[1]. In our previous paper[1] on the TRIAD method we present next generation sequencing data that suggest no appreciable bias of the engineered Mu transposon towards GC rich sites as reported earlier[2]. Quality control of the deletion library (10 randomly selected variants sent for sequencing) confirmed 100% success rate of random 3 bp deletion along the $anc^{HLD\text{-}RLuc}$ gene. Deletions in randomly selected sequenced variants occurred at positions: 55, 63, 128, 151, 202, 209, 223, 287, 291 and 307. Quality control of the insertion library (10 randomly selected variants sent for sequencing) confirmed 50% success rate of random 3 bp insertion along the $anc^{HLD\text{-}RLuc}$. Insertions in randomly selected sequenced variants occurred at positions: 18, 120, 186, 227 and 309. The rest of the libraries contained 1 or 2 bp insertion causing a shift of reading frame that led to a stop codon insertion. The theoretical diversity at the gene level of the $anc^{HLD\text{-}RLuc}$ three-nucleotide deletion library is 918 variants, and of the insertion library is 58,752 variants (inserted triplet NNN), with the additional possibility of a juxtaposed point substitution. To determine the effects of InDels on the ancestral template, 968 deletion variants and 2,696 insertion variants were screened for enhanced LUC activity towards coelenterazine in microtiter plates. 968 deletion variants and 1,056 insertion variants were screened for HLD activity towards 1-bromobutane in microtiter plates. The insertion library of AncINS was constructed analogously to insertion library of $Anc^{HLD\text{-}RLuc}$ with the same success rate and 880 variants were screened for LUC activity towards coelenterazine.

Supplementary Table 1. A list of variants that exhibited ≥ 100-fold higher LUC activity in screening towards coelenterazine compared to the template Anc$^{HLD\text{-}RLuc}$ during the screening of the 3bp insertion and the 3bp deletion library of *anc*$^{HLD\text{-}RLuc}$ in microtiter plates. Hot spot regions where the insertion or deletion event took place are α4 helix, L9 and L14 loops. The mutants selected for small-scale purification and characterization are highlighted in bold.

	Variant of Anc$^{HLD\text{-}RLuc}$	Location
1	**G150_W151insF**	L9 loop
2	**G150_W151RinsF**	
3	G150_W151insI	
4	G150_W151insV	
5	G150_W151insL	
6	G150_W151insY	
7	F154_P155insF	
8	D160_I161insL	α4 helix
9	**I161_F162insL**	
10	I161_F162GinsL	
11	I161_F162IinsL	
12	**I161_F162PinsL**	
13	I161_F162SinsL	
14	I161_F162VinsL	
15	I161T_F162insF	
16	**F162_Q163insF**	
17	**F162_Q163insP**	
18	F162_Q163EinsP	
19	F162_Q163insT	
20	F162_Q163insH	
21	F162_Q163EinsH	
22	**F162_Q163insY**	
23	**delQ163-A164P**	
24	**P223_I224insA**	L14 loop
25	**P223_I224insS**	
26	**P223_I224insT**	

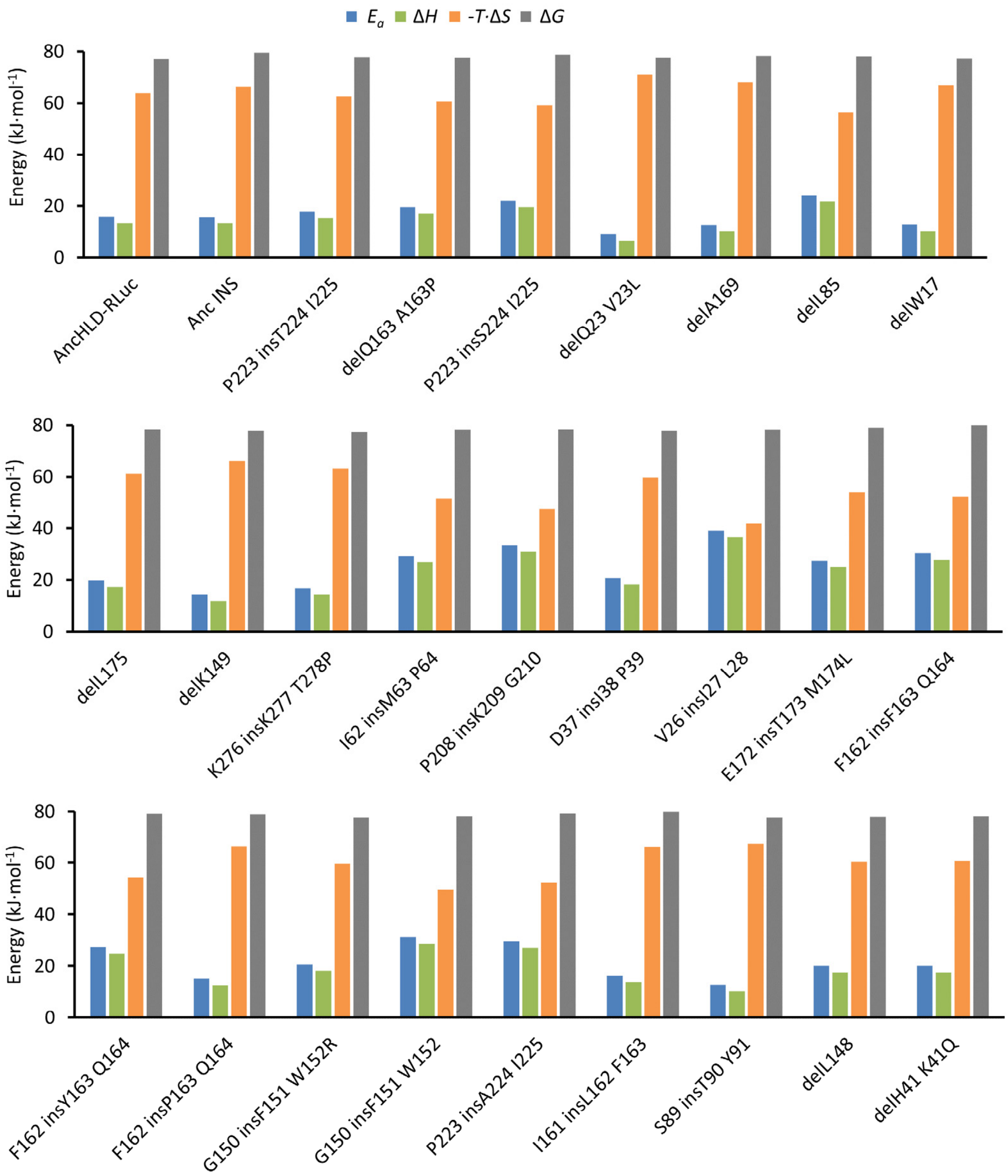

Supplementary Figure 1. Summary of the thermodynamics data. Data were collected for HLD reaction of InDel mutants using the capillary microfluidics in 1 mM HEPES and 20mM Na_2SO_4 at pH 8.2. The data represent the averages of 10 replicates at 8 different temperatures (20°C to 40 °C). E_a = activation energy, ΔH = enthalpy of activation, $-T.\ \Delta S$ = entropy of activation powered by the absolute temperature, ΔG = Gibbs energy. All thermodynamic terms are in the same units of $kJ.mol^{-1}$.

Supplementary Note 2. Anisotropic Network Model. The Anisotropic Network Model (ANM)[3,4] is an elastic network-based analytical approach[5]. It is an application of normal mode analysis aimed to predict protein motions from their crystallographic three-dimensional coordinates. ANM is a coarse-grained model that represents the protein as a mesh of beads and springs. The beads represent mass units in the protein, generally each of its amino acids, and the springs the interactions among them. Such interactions are limited in a distance radius and are modelled by a single harmonic potential. ANM specifically accounts for direction preferences of each of the identified motions. The inputs of the calculation are the three-dimensional coordinates of the beads in the system (alpha carbons of the protein) and the radius threshold for interactions. The output used in this work is the cross-correlation of the predicted motions of each pair of beads in the system, this is whether any two residues are predicted to move along the same direction over space and time. This residue-residue cross-correlation of motions is extended to the cross-correlation of the predicted motions of a single residue and a number of consecutive ones (residue-segment), or the cross-correlation of the predicted motions of two different sets of consecutive residues (segment-segment). The calculation of segment-centred cross-correlation values from Anisotropic Network Model is explained in the corresponding section in the Supplementary Methods. The boundaries (beginning and ending) of such segments need to be predefined. We used the boundaries of the regular secondary structure elements in our analysed systems to define the segments for the extended cross-correlation analysis under the assumption that the residues forming a regular secondary structure element are to some extent constricted to a solidary motion[6]. The sets of boundaries used for RLuc8 and $Anc^{HLD\text{-}RLuc}$ are given in Tables S2 and S3, respectively. These boundaries are not part of the standard input of ANM, and are only required for the extended application of the cross-correlation of motions analysis we propose in this manuscript.

Supplementary Table 2. Manually edited boundaries of secondary structure elements in RLuc8. First and second columns, respectively, correspond to the index of the amino acid starting and ending a given secondary structure element; the third column shows the name given to such element in this study.

INI	END	NAME
17	23	H0
24	24	L0
25	29	E1
30	31	L1
32	38	E2
39	44	L2
45	50	E3
51	56	L3
57	70	H1
71	71	L4
72	76	E4
77	93	L5
94	108	H2
109	113	L6
114	119	E5
120	132	H3
133	138	L8
139	143	E6
144	159	L9
160	167	H4
168	168	L10
169	178	H5'
179	179	L11
180	189	H5
190	195	L12
196	204	H6
205	210	L13
211	222	H7
223	230	L14
231	246	H8
247	251	L15
252	258	E7
259	262	L16
263	272	H9
273	275	L17
276	283	E8
284	286	L18
287	290	H10'
291	291	L19
292	309	H10

Supplementary Table 3. Manually edited boundaries of secondary structure elements in Anc$^{HLD\text{-}RLuc}$. First and second columns respectively correspond to the index of the amino acid starting and ending a given secondary structure element; the third column shows the name given to such element in this study.

INI	END	NAME
3	9	H0
10	10	L0
11	15	E1
16	17	L1
18	24	E2
25	32	L2
33	38	E3
39	43	L3
44	57	H1
58	58	L4
59	63	E4
64	80	L5
81	94	H2
95	100	L6
101	106	E5
107	119	H3
120	124	L8
125	130	E6
131	147	L9
148	155	H4
156	156	L10
157	166	H5'
167	167	L11
168	177	H5
178	183	L12
184	192	H6
193	198	L13
199	210	H7
211	218	L14
219	234	H8
235	239	L15
240	246	E7
247	249	L16
250	258	H9
259	263	L17
264	271	E8
272	274	L18
275	278	H10'
279	279	L19
280	294	H10

Supplementary Note 3. Target regions for Anisotropic Network Model (ANM) cross-correlations. Loops 9, 14 and 16 were defined as regions of interest based on their previously described properties[7,8]. Loops 3 and 18 and strand 5 (corresponding to the 0-length loop 7) were considered because they encompassed catalytic residues[7,8]. Loops 6, 13, and 17 were picked as representative surface loops for comparison purposes (negative control).

Supplementary Note 4. Partial Least Squares (PLS). Partial Least Squares (PLS) is a multivariate statistical analysis for comparing response outcomes (Y) and explanatory variables (X). The method is well suited for the analysis of situations where the number of explanatory variables exceed the number of samples or are highly collinear[9]. PLS explores the data structure to find the set of underlying or latent variables that account for most of the variation in the response. Both response outcomes and explanatory variables are projected to a latent space that maximizes the covariance in between them. As a result, a model using a smaller number of latent variables that maximally explain the response outcomes is obtained where the contribution or weight of each of the original explanatory variables to the model can be assessed. The final model describes the relative importance of each relevant explanatory variable (X) for the prediction of the response outcomes (Y). In the work herein presented, the response outcomes are the levels of both luciferase and haloalkane dehalogenase activities, and the explanatory variables those summarized in Source Data File. To assess the quality of the developed PLS model and internal validation can be performed[10] by cross-validation and permutation testing. During cross-validation[11], a portion of the Y data is excluded during model development, and the resulting model is used to predict the missing data. The predictions are then compared to the original data to obtain a Q^2 value, which provides a more realistic estimate of a model predictive power than the squared regression coefficient R^2. PLS was applied for the analysis of data patterns in order to better understand subjacent structure-function relationships in the data (Supplementary Figures 2, 3). A statistically significant model for LUC and HLD activity as dependent variables was constructed. The model has two components and shows the following statistics: R^2 = 0.73 and Q^2 = 0.67 for LUC activity (n = 25) and R^2 = 0.63 and Q^2 = 0.54 for HLD activity (n = 25). Cross-validation and permutation tests confirmed the lack of chance correlation and thus a good predictive power for our model. The small gap between R^2 and Q^2 (Supplementary Figure 4) values suggests that the model is not over-fitted and thus the variables fixed in the model contribute to explanation of the measured enzymatic activities.

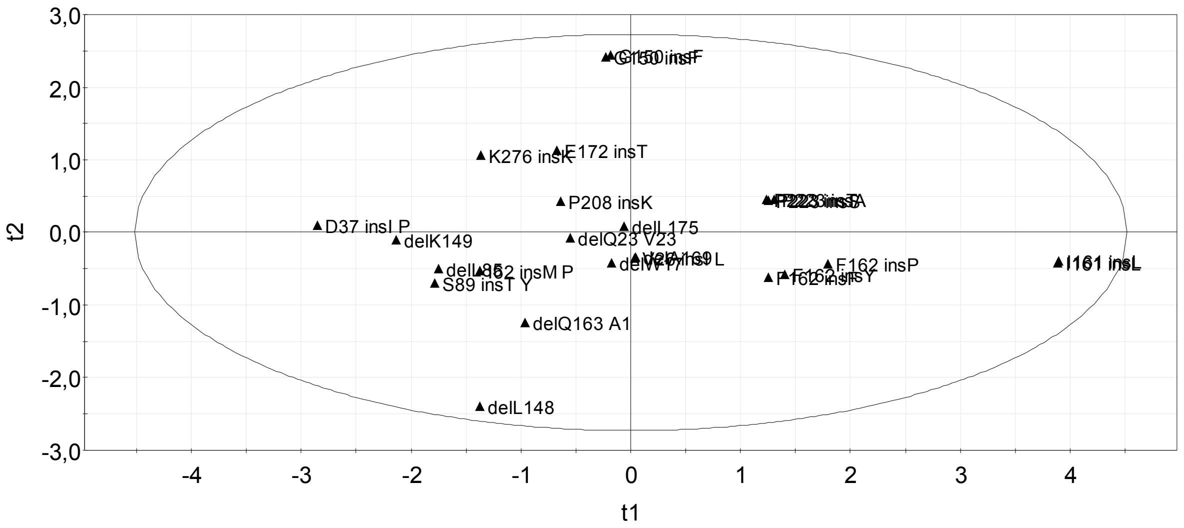

Supplementary Figure 2. Scores plot t1 *versus* t2 from the Partial Least Squares analysis. The plot shows the distribution of 25 variants along to two principal components t1 and t2. Position of the variants in the space directly corresponds to the position of the variables in the loadings plot. Mutants with similar properties are located close to each other, while those with different properties are far away.

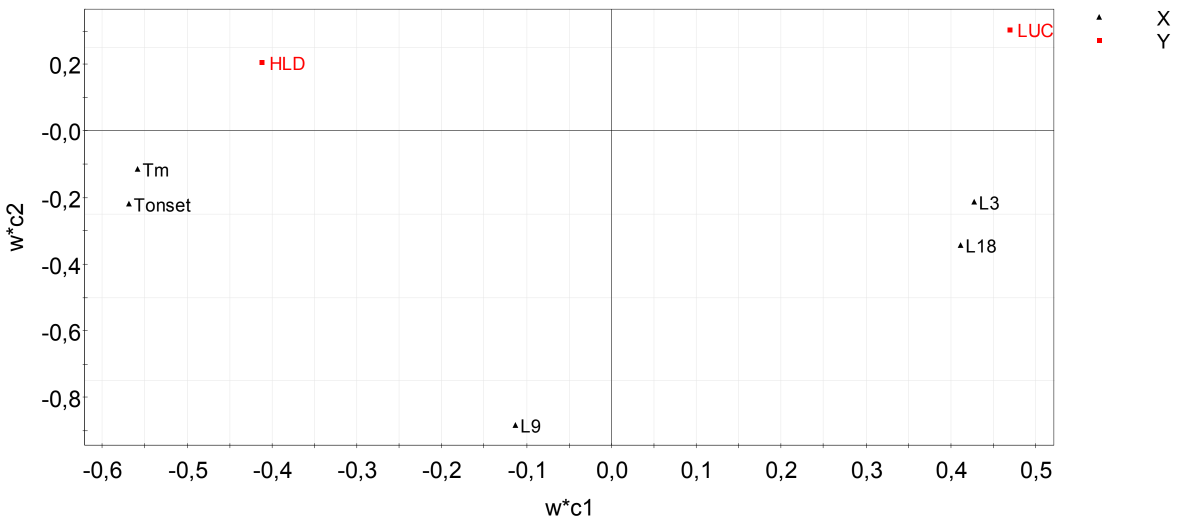

Supplementary Figure 3. Loadings plot wc1 *versus* wc2 from the Partial Least Squares analysis. The plot shows the distribution of variables along with two principal components wc1 and wc2. Position of the variables in the space corresponds to the position of mutants in the scores plot.

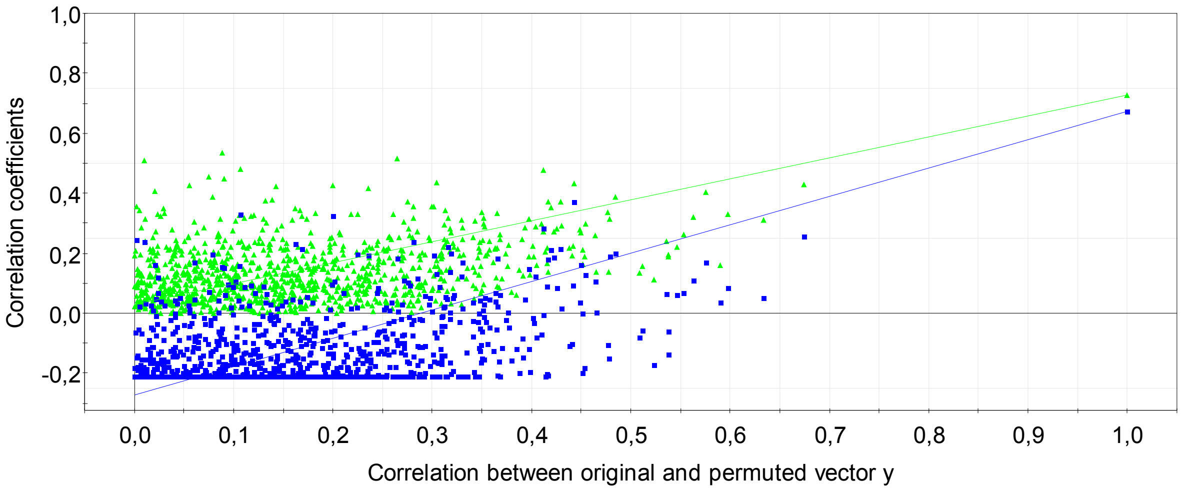

Supplementary Figure 4. Validation plot from permutation testing of the Partial Least Squares model. The plot presents 999 permutations of the original vector y. R^2 values are shown in green, Q^2 values are shown in blue. As the correlation between permuted and original y vectors decreases, the coefficients of determination for the permuted data show significantly lower values than those obtained for the model (the rightmost points), confirming that the results are unlikely to be based on the chance correlation.

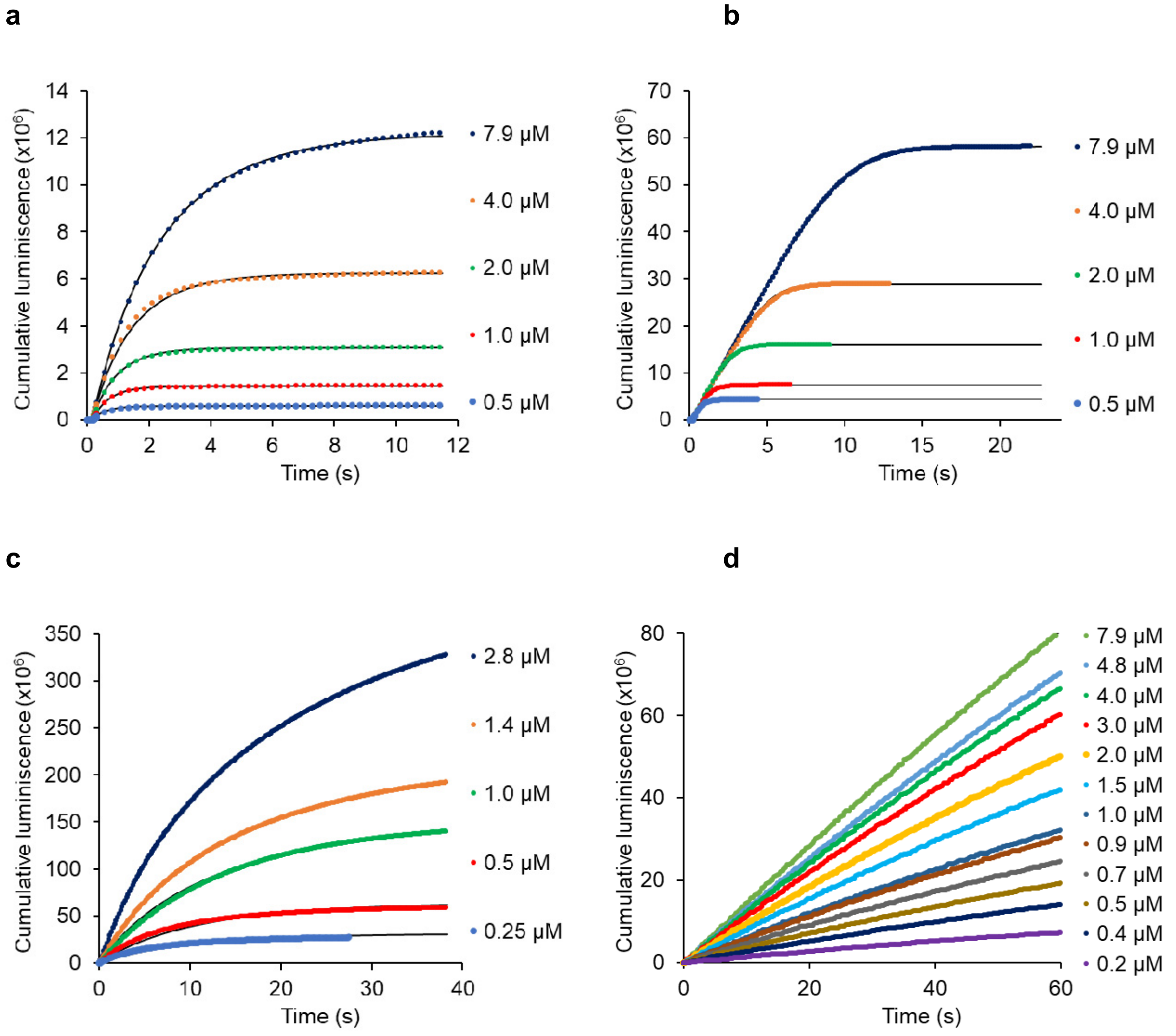

Supplementary Figure 5. Steady-state kinetic data. Progress curves obtained by transformation of luminescence data recorded upon mixing 0.017 µM RLuc8 with 0.5 to 7.9 µM coelenterazine **a**, 0.13 µM AncFT with 0.5 to 7.9 µM coelenterazine **b**, 0.063 µM AncINS with 0.25 to 2.8 µM coelenterazine **c**, and 0.64 µM Anc$^{HLD\text{-}RLuc}$ with 0.2 to 7.9 µM coelenterazine **d**. Each progress curve was measured in 3 replicates and then averaged. The solid lines represent the best fit.

Supplementary Table 4. Steady-state kinetic parameters, their respective standard errors (S.E.), and confidence intervals determined for the final set of enzymes with coelenterazine. The experiments were carried out in triplicate in 100 mM phosphate buffer at pH 7.5 and temperature 37 °C. The standard errors were calculated from the covariance matrix during nonlinear regression. The lower and upper limits for steady-state kinetic parameters were derived by using confidence contour analysis applying $\chi2$ threshold at the boundary of 0.9.

Parameter	$Anc^{HLD\text{-}RLuc}$		AncINS		AncFT		RLuc8		RLuc8[12]		RLuc[12]	
	Value ± S.E.	Confidence intervals	Value ± S.E.	Confidence intervals	Value ± S.E.	Confidence intervals	Value ± S.E.	Confidence intervals	Value ± S.E.	Confidence intervals	Value ± S.E.	Confidence intervals
K_m [µM]	1.329 ± 0.001	1.31–1.35	4.6 ± 0.2	3.3–6.9	0.064 ± 0.001	0.017–0.100	1.5 ± 0.1	1.27–1.92	1.6 ± 0.2	n.d.	2.9 ± 1.0	n.d.
k_{cat} [s^{-1}]	(3.09 ± 0.01) × 10^{-4}	(3.06–3.11) × 10^{-4}	0.134 ± 0.005	0.113–0.167	0.111 ± 0.001	0.109–0.112	4.7 ± 0.1	4.5–5.1	4.9 ± 0.1	n.d.	3.9 ± 0.4	n.d.
K_p [µM]	0.37 ± 0.01	0.34–0.41	1.02 ± 0.02	0.92–1.15	0.5 ± 0.1	0.14–0.85	1.18 ± 0.05	0.97–1.41	n.d.	n.d.	n.d.	n.d.
k_{cat}/K_m [$\mu M^{-1}.s^{-1}$]	(2.33 ± 0.01) × 10^{-4}	(2.27–2.37) × 10^{-4}	0.029 ± 0.001	0.028–0.031	1.6 ± 0.2	1.0–5.2	3.0 ± 0.1	2.7–3.6	3.1 ± 0.4 [a]	n.d.	1.3 ± 0.5 [a]	n.d.
K_m/K_p	3.6 ± 0.1 [a]	n.d.	4.5 ± 0.2 [a]	n.d.	0.13 ± 0.03 [a]	n.d.	1.3 ± 0.1 [a]	n.d.	n.d.	n.d.	n.d.	n.d.

[a] error estimate calculated from respective standard error estimates and values of measured parameters with consideration of the propagation of arithmetic error; n.d. = not determined

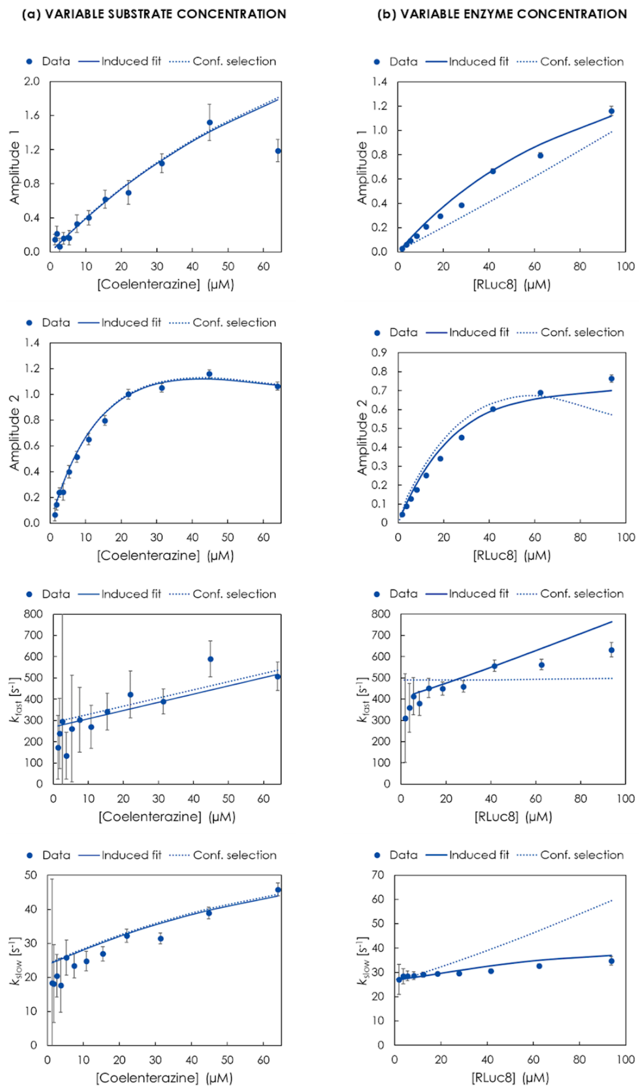

Supplementary Figure 6. Concentration dependence of amplitudes and observed rates of the double exponential fluorescence decrease observed upon binding of coelenterazine by RLuc8. The experiment was performed in 100 mM potassium phosphate buffer pH 7.5 at 15 °C and each kinetic trace was measured in 7 replicates and averaged. The experimental values and their respective standard errors were obtained from the double-exponential fit of the tryptophan fluorescence signal. The solid and the dashed lines represent the best fit according to the induced-fit mechanism and the conformational selection mechanism, respectively. The datapoints are presented as best fit values ± standard errors (S.E.) calculated from nonlinear exponential regression. The values of k_{fast} and k_{slow} correspond to exponential observed rates of the initial fast phase and the following slow phase, respectively. **a** The standard concentration dependence based on the variable concentration of the substrate cannot distinguish between the induced fit and the conformational selection mechanism when measured alone. **b** Including the concentration dependence based on the variable enzyme concentration allowed the identification of the correct mechanism which occurs during coelenterazine binding.

Supplementary Note 5. Analytical fitting of observed rates and amplitudes together with numerical fitting of the raw kinetic data provided satisfying results with well-constrained parameters for a two-step substrate binding mechanism in the case of AncINS, AncFT, and RLuc8. More complex mechanisms (thermodynamic cycle, conformational selection followed by induced fit in a sequence, etc.) were tested as well but these scenarios did not improve the goodness of the fit and, in contrast, expanded the model with additional rate constants whose values were not well constrained. This led to the conclusion that the two-step binding mechanism was sufficiently accounting for the data. To differentiate between the two possible and until then indistinguishable two-step mechanisms, induced fit or conformational selection (Supplementary Figure 6a), a targeted experiment using a variable concentration of an enzyme was performed. When the trends of observed rates and amplitudes were compared with synthetic data generated by the KinTek Explorer software (KinTek Corporation, USA)[13–15] using the previously determined rate constants, the experimental data points were in a very good agreement with the predicted trends according to the induced-fit mechanism (Supplementary Figure 6b). Additionally, the global numerical fitting of both the variable substrate and the variable enzyme datasets clearly distinguished between the two assumed mechanisms when the conformational selection model yielded significantly worse χ^2 values, and the determined rate constants were not sufficiently constrained, as revealed by the confidence contour analysis[15]. For all these reasons, it was concluded that the binding of the coelenterazine substrate occurred via the induced-fit mechanism as the minimal mechanism sufficiently accounting for all the experimental data while providing satisfactory statistical parameters. The values, standard errors, and lower bound and upper bound limits of all the variable parameters obtained by the global fitting are summarized in Supplementary Table 5.

Supplementary Table 5. Values, standard errors (S.E.), and confidence intervals of parameters describing coelenterazine binding by the final set of enzymes. The experiments were carried out in 100 mM phosphate buffer at pH 7.5 and temperature 15 °C. The parameters and their statistics were obtained by global fitting using numerical integration and by confidence contour analysis for χ^2 threshold of 0.95.

Parameter	AncHLD-RLuc		AncINS		AncFT		RLuc8	
	Value ± S.E.	Confidence intervals	Value ± S.E.	Confidence intervals	Value ± S.E.	Confidence intervals	Value ± S.E.	Confidence intervals
k_{+1} [μM^{-1}.s^{-1}]	0.003 ± 0.003	0–0.061	2.26 ± 0.06	1.45–2.59	5.21 ± 0.07	4.65–6.56	2.35 ± 0.04	2.07–2.94
k_{-1} [s^{-1}]	0.118 ± 0.071	0–3.77	86.8 ± 2.6	44.4–107	367 ± 5	295–515	305 ± 5	222–392
k_{+2} [s^{-1}]	n.a.	n.a.	3.61 ± 0.27	2.31–6.70	31.9 ± 1.1	23.8–43.2	26.6 ± 1.1	19.4–35.9
k_{-2} [s^{-1}]	n.a.	n.a.	4.60 ± 0.10	2.94–5.75	27.5 ± 0.4	22.4–35.0	23.4 ± 0.2	21.3–26.7
a	0.905 ± 0.017	0–0.968	0.775 ± 0.004	0.735–0.791	0.047 ± 0.010	0–0.074	0.211 ± 0.013	0.106–0.289
b	n.a.	n.a.	0.095 ± 0.047	0–0.396	0.042 ± 0.012	0–0.061	0.022 ± 0.019	0–0.063
f_1	2.097 ± 0.002	2.09–2.11	1.248 ± 0.001	1.24–1.25	2.382 ± 0.002	2.38–2.39	2.782 ± 0.002	2.78–2.79
f_2	0.079 ± 0.001	0.078–0.080	0.184 ± 0.001	0.183–0.184	0.242 ± 0.001	0.242–0.243	0.234 ± 0.001	0.233–0.235

n.a. = not applicable

Supplementary Note 6. Analysis of substrate binding by transient kinetics. Kinetic traces collected for AncINS, AncFT, and RLuc8 exhibited a triple-exponential decay of the fluorescence signal. The first two kinetic phases were significantly faster and corresponded to the steps preceding the chemical transformation of the substrate. Due to its overlapping with the rise of the luminescence signal generated by the oxidative cleavage of coelenterazine (Supplementary Figure 7), the third exponential phase was assigned as a chemical transformation step. Since the rate of the chemical step was significantly slower, a rapid equilibrium of the prior steps was assumed, and a separate analysis of the substrate-binding process was feasible (Supplementary Figure 6, 8). Decreasing the temperature of the experiment down to 15 °C resulted in capturing both the fast and the slow kinetic phase while preparing the substrate at concentrations close to solubility limits allowed independent determination of all the elementary rate constants describing the substrate-binding process. Confidence intervals of all the kinetic parameters and a full description of kinetic analyses are provided in Supplementary Methods.

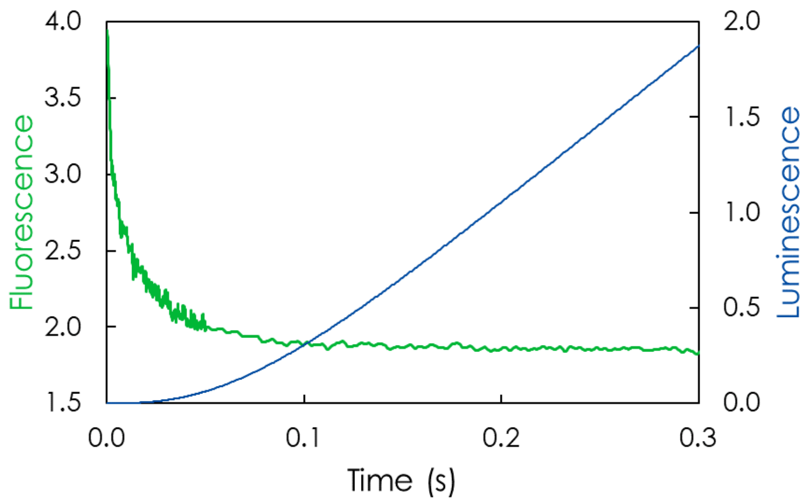

Supplementary Figure 7. Comparison of fluorescence/luminescence changes during the reaction catalysed by RLuc8. The change in native tryptophan fluorescence (green) exhibits three exponential phases. The two initial phases overlap with a lag phase of the luminescence signal (blue) while the third phase overlaps with an increase of the luminescence signal produced during the reaction. Kinetic experiments were carried out in 100 mM potassium phosphate buffer at 15 °C and pH 7.5.

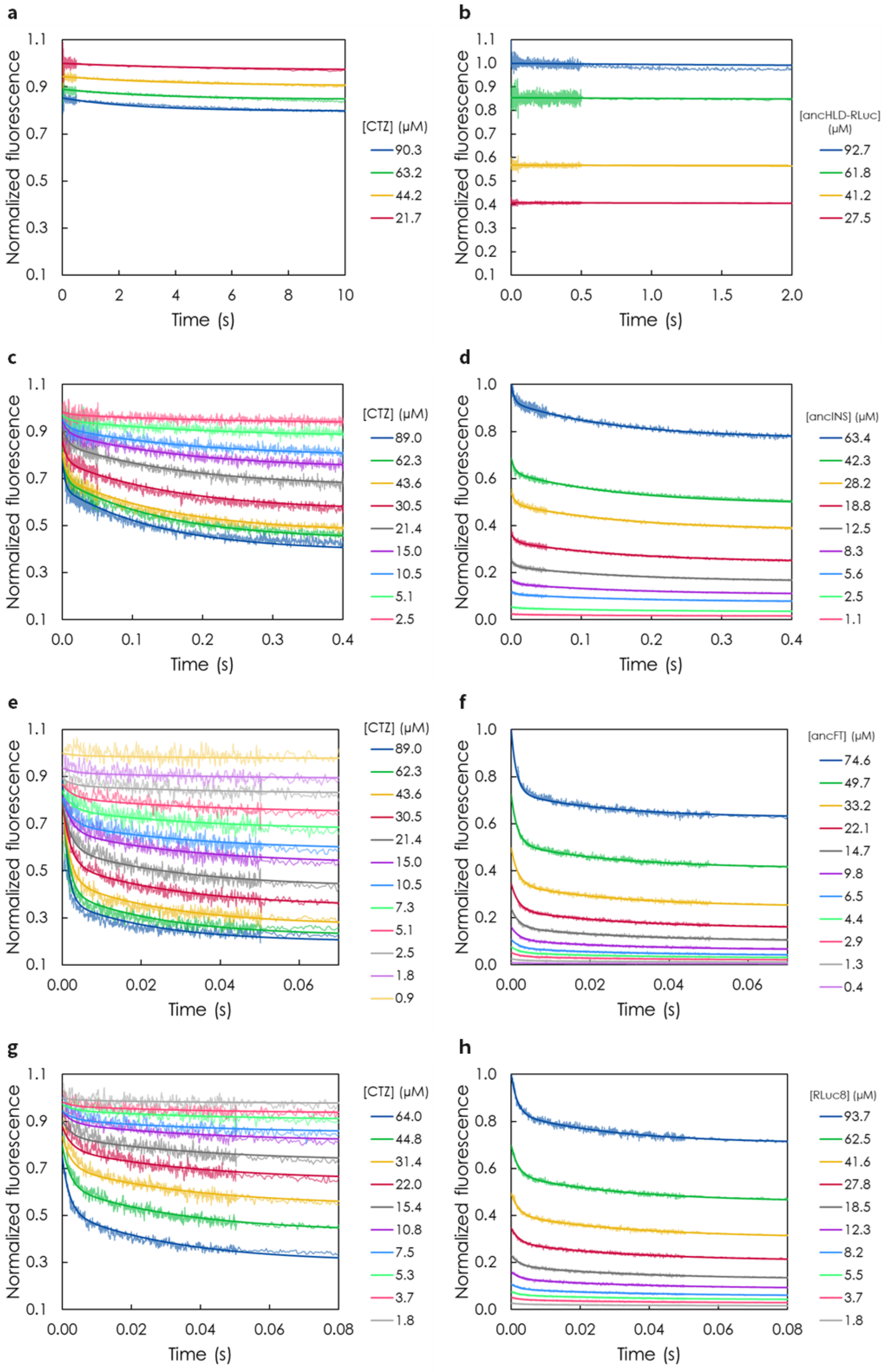

Supplementary Figure 8. Time course of the tryptophan fluorescence signal quenching upon binding of the coelenterazine substrate. The experiment was performed in 100 mM potassium phosphate buffer pH 7.5 at 15 °C. Each fluorescence trace was

measured in 7 consecutive replicates and then averaged. Solid lines represent the best fit. **a** Fixed 2.5 μM concentration of Anc$^{HLD\text{-}RLuc}$ mixed with varying concentration of coelenterazine. **b** Varying concentration of Anc$^{HLD\text{-}RLuc}$ mixed with fixed 16 μM concentration of coelenterazine. **c** Fixed 2.9 μM concentration of AncINS mixed with varying concentration of coelenterazine. **d** Varying concentration of AncINS mixed with fixed 16 μM concentration of coelenterazine. **e** Fixed 2.6 μM concentration of AncFT mixed with varying concentration of coelenterazine. **f** Varying concentration of AncFT mixed with fixed 16 μM concentration of coelenterazine. **g** Fixed 2.0 μM concentration of RLuc8 mixed with varying concentration of coelenterazine. **h** Varying concentration of RLuc8 mixed with fixed 16 μM concentration of coelenterazine.

Supplementary Table 6. Crystallographic data collection and refinement statistics.

	AncINS	AncFT	RLuc8-W121F/E144Q–CEI
**Data collection***			
Space group	$P2_12_12_1$	$P3_121$	$I222$
Cell dimensions			
a, b, c (Å)	44.71, 84.17, 160.26	87.556, 87.556, 102.125	112.765, 131.077, 142.03
α, β, γ (°)	90, 90, 90	90, 90, 120	90, 90, 90
Resolution (Å)	45.09 – 2.001 (2.073 – 2.001)	43.78 – 1.953 (2.023 – 1.953)	44.53 – 1.9 (1.968 – 1.9)
Total reflections	363,814 (33,322)	669,493 (66,368)	1,120,312 (103,858)
Unique reflections	41,669 (4,050)	33,335 (3,277)	82,699 (8,143)
*R*merge	7.69 (68.15)	14.08 (129.2)	11.04 (189)
I / σI	16.5 (2.5)	19.9 (2.9)	19.9 (1.4)
Completeness (%)	99.6 (98.2)	99.9 (99.3)	99.9 (99.2)
Multiplicity	8.7 (8.2)	20.1 (20.3)	13.5 (12.8)
CC(1/2)	0.999 (0.953)	0.999 (0.871)	0.999 (0.538)
**Refinement**			
Resolution (Å)	45.09 – 2.001	43.78 – 1.953	44.53 – 1.9
No. reflections	41,595 (4,029)	33,321 (3,274)	82,699 (8,135)
*R*work / *R*free (%)	17.1 / 21.5	29.1 / 33.0**	17.2 / 19.6
No. atoms			
Protein	4,821	2,405	5,092
Ligand	–	–	73
Water	175	140	508
B-factors			
Protein	43.3	28.4	33.9
Ligand	–	–	41.3
Water	41.2	32.7	40.0
R.m.s deviations			
Bond lengths (Å)	0.008	0.007	0.007
Bond angles (º)	1.01	0.92	0.84
PDB ID	6S6E	6S97	6YN2

* One crystal was used for each structure. Values in parentheses are for the highest-resolution shell.

** Higher *R*work and *R*free values are due to the void (uninterpretable) electron density present in the asymmetric unit of the crystal.

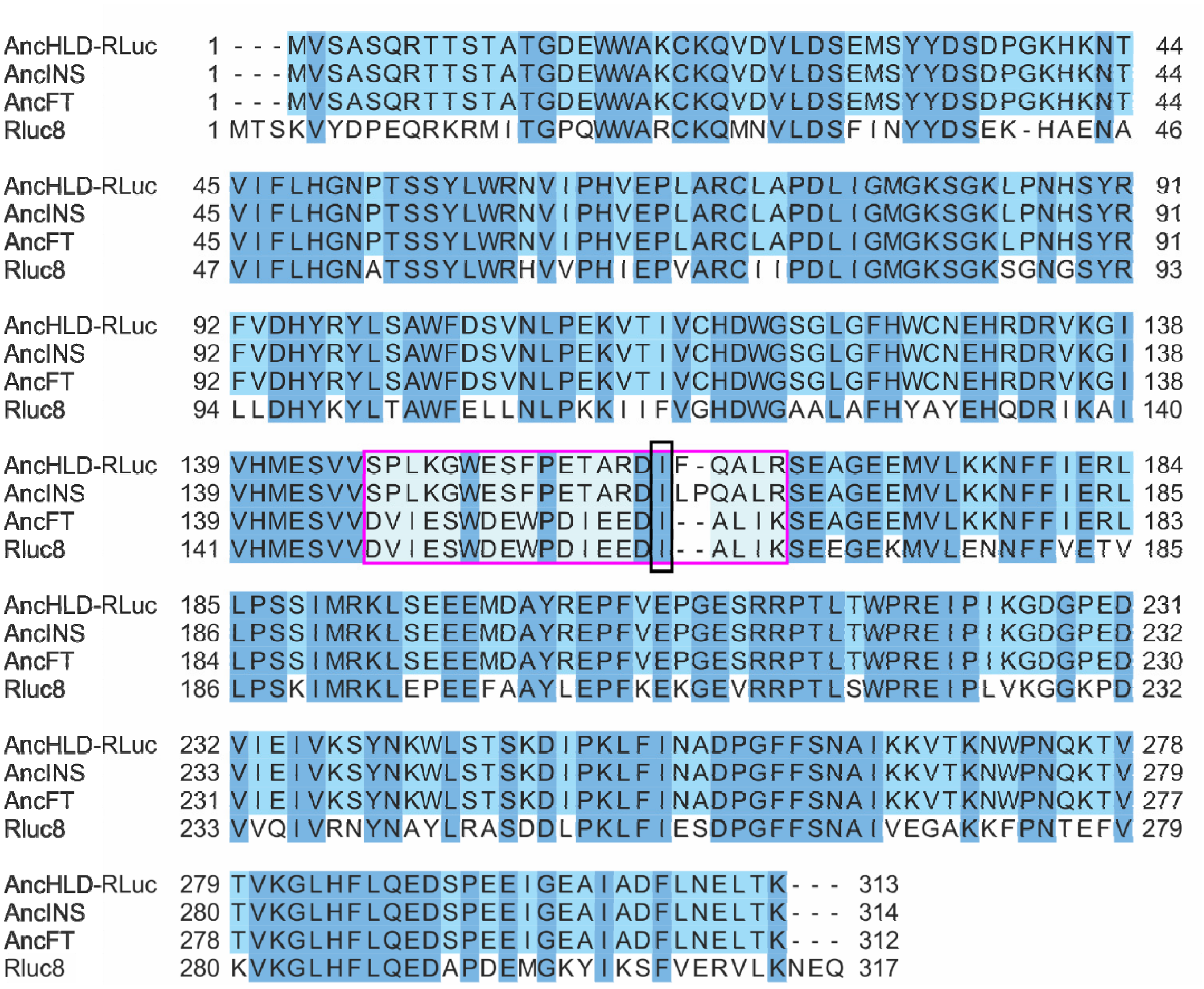

Supplementary Figure 9. Sequence alignment of Anc$^{HLD\text{-}RLuc}$, AncINS, AncFT and RLuc8. The region selected for fragment transplantation is highlighted in a magenta rectangle. I161 (I163 in RLuc) is highlighted in a black rectangle.

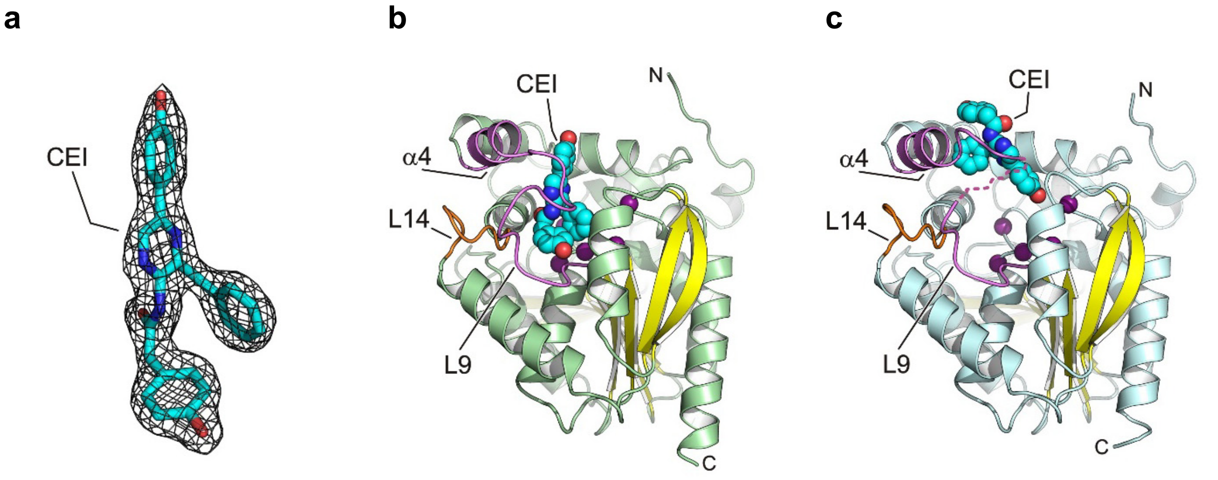

Supplementary Figure 10. Binding mode of coelenteramide to RLuc8-W121F/E144Q. a Simulated annealing omit electron density map contoured at 2 σ for coelenteramide (CEI). **b** Structure of the CEI bound RLuc8-W121F/E144Q (PDB ID: 6YN2). The CEI is shown as cyan space-filling spheres. **c** Structure of the CEI bound RLuc8 (PDB ID: 2PSJ). The CEI is shown as cyan space-filling spheres. The residues of the conserved catalytic pentad are shown as purple spheres; the central eight-stranded β-sheet is in yellow; the α4 helix and L9 loop are in violet, and L14 loop is in orange.

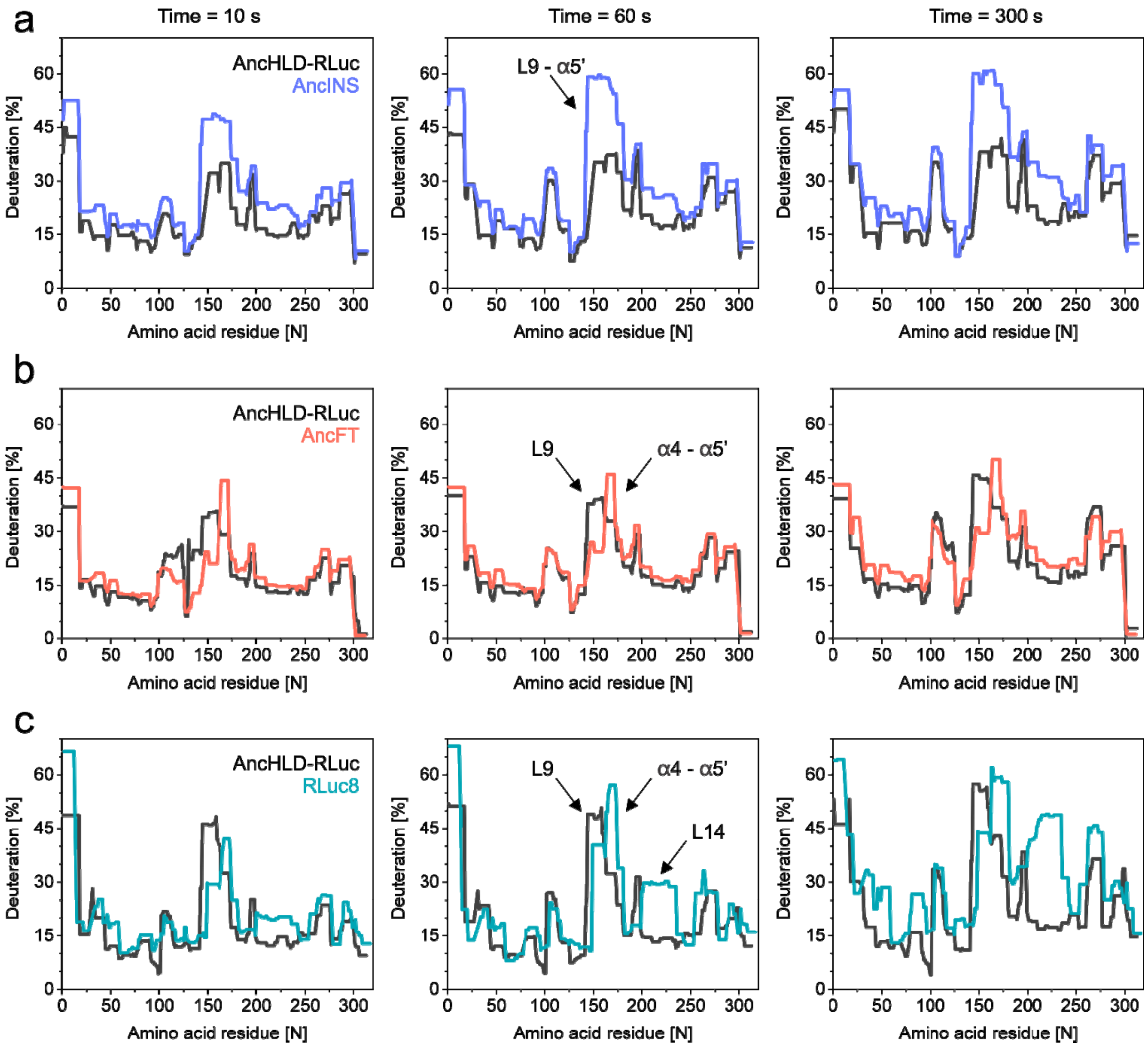

Supplementary Figure 11. Hydrogen-deuterium exchange mass spectrometry (HDX-MS) reveals changes in solvation and dynamics. The HDX was carried out at room temperature and was quenched at 10 s, 60 s, and 300 s by the addition of 1 M HCl in 1 M glycine with pepsin. Since the analysis of AncINS (slate), AncFT (salmon), and RLuc8 (teal) was done in different time periods, a freshly prepared sample of $Anc^{HLD\text{-}RLuc}$ (black) was always used as a template. **a** Deuteration of AncINS is notably higher in the cap domain between position 142 (L9 loop) and 174 (α5' helix) compared to $Anc^{HLD\text{-}RLuc}$. This region includes the α4 helix where the insertion and substitution took place. **b** The biggest difference in deuteration between $Anc^{HLD\text{-}RLuc}$ and AncFT is in the fragment transplanted region of the L9 loop and the α4 helix. In $Anc^{HLD\text{-}RLuc}$, deuteration is high in the L9 region and is decreasing towards α4–α5'. Contrarily, in AncFT deuteration in L9 is lower and is increasing towards α4–α5'. **c** RLuc8 has similar deuteration profile like AncFT in the region of L9–α5', while deuteration of L14 is higher compared to $Anc^{HLD\text{-}RLuc}$, as well as AncINS and AncFT. HDX-MS was measured for each variant once, $Anc^{HLD\text{-}RLuc}$ was measured in triplicate.

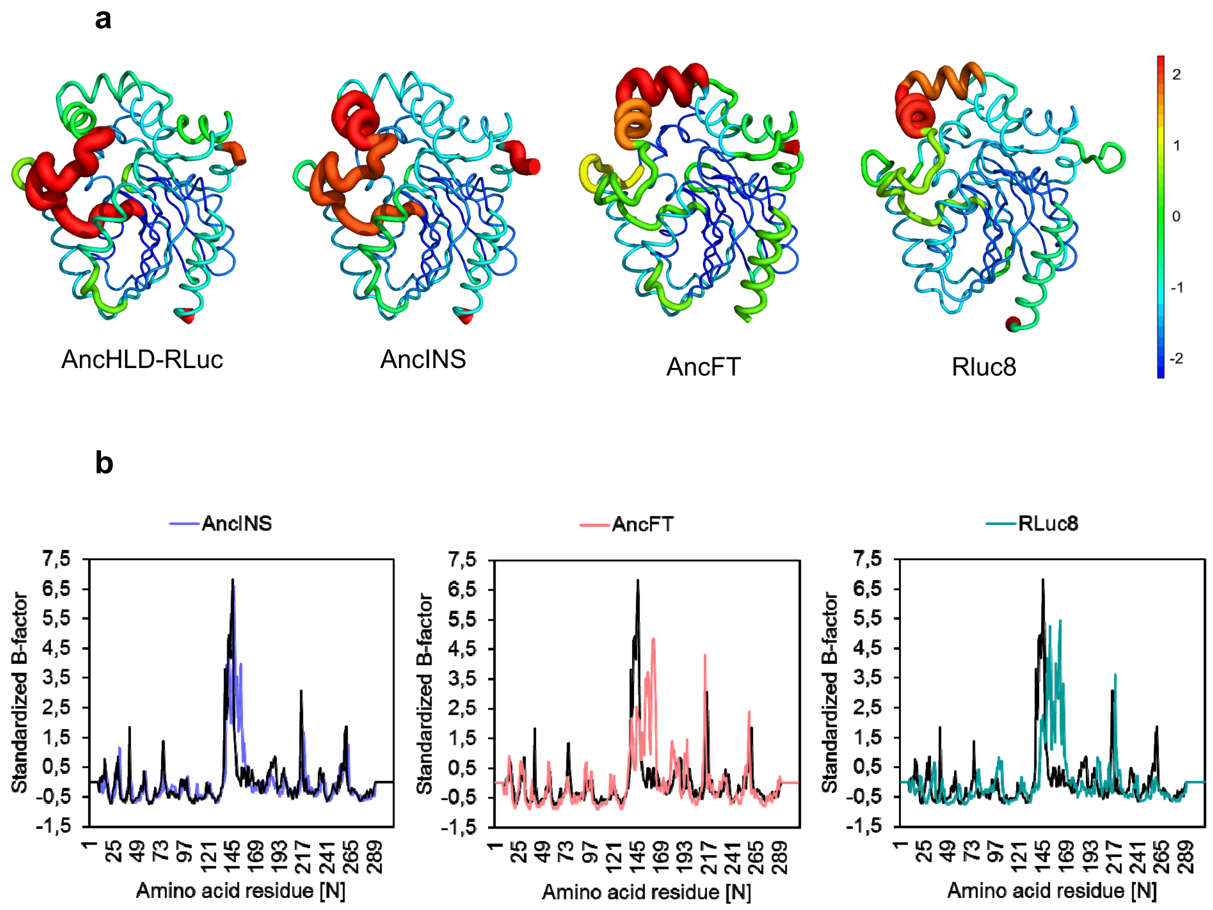

Supplementary Figure 12. Conformational dynamics of studied proteins observed in molecular dynamics simulations. a B-factors of backbone atoms standardized across all protein variants, ranging from -2 (blue) to 2 (red). B-factor values standardized per each protein are indicated by the thickness of the lines representing the protein backbone. Values were averaged per secondary structure elements. **b** Standardized B-factors for backbone atoms. $Anc^{HLD\text{-}RLuc}$ template is depicted as a black line.

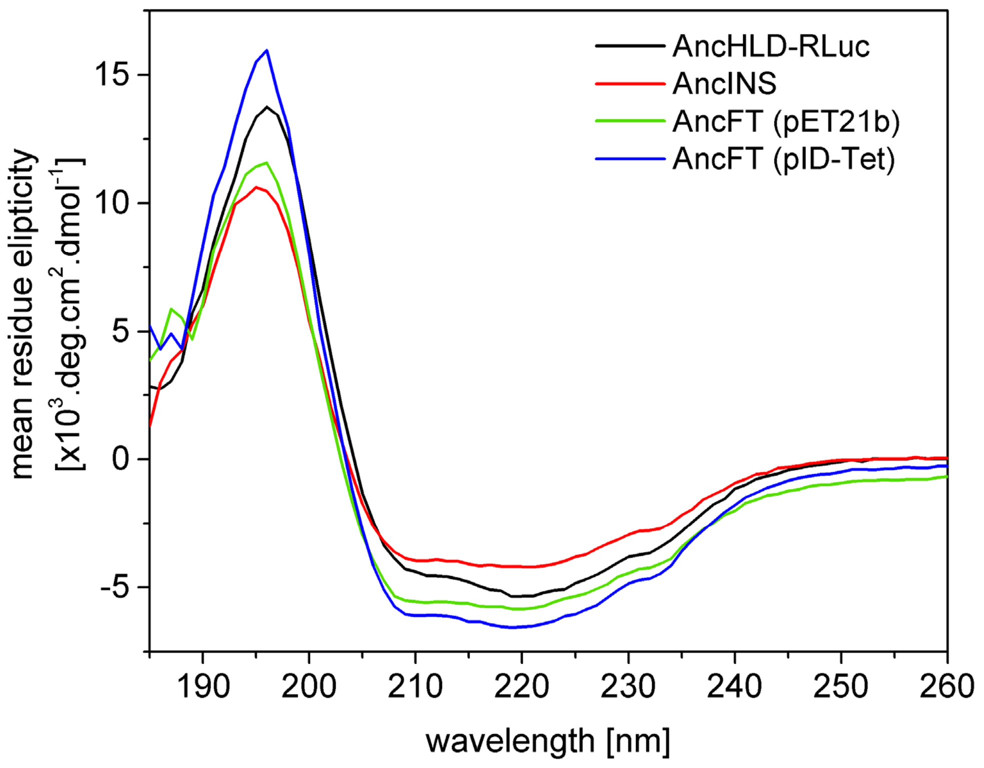

Supplementary Figure 13. Far-UV circular dichroism spectra of Anc$^{HLD-RLuc}$ (black), AncINS (red), and AncFT expressed either from pET21b (green) or pID-Tet (blue) based construct. All the spectra show one positive peak at 195 nm and two negative minima at 210 and 220 nm, characteristic of α-helical content. Circular dichroism (CD) spectra were recorded at 20 °C using a spectropolarimeter (Chirascan, Applied Photophysics, United Kingdom). Data were collected from 185 to 260 nm, at 100 nm/min, with a 1 s response time and a 1 nm bandwidth in a 0.1 cm quartz cuvette. Each spectrum shown is the average of five individual scans and was corrected for the absorbance caused by the buffer.

Supplementary Table 7. Stability of bioluminescence signal. Half-life ($t_{1/2}$) and an initial signal steadiness evaluated from the bioluminescence kinetic data of proteins purified from bacterial cells cultures. Experiments were repeated independently three times with consistent results.

	Half-life (min)	Initial signal stability [a] (min)	Initial signal (rel. RLU)
AncHLD-Rluc	n.d.	n.d.	0.0002
AncINS	10.3 ± 0.1	3.0	0.03
AncFT	19.5 ± 0.1	6.9	0.03
RLuc8	0.17 ± 0.01	0.03	1

[a] the time for which the signal stays greater than 80% of initial value

n.d. not determined

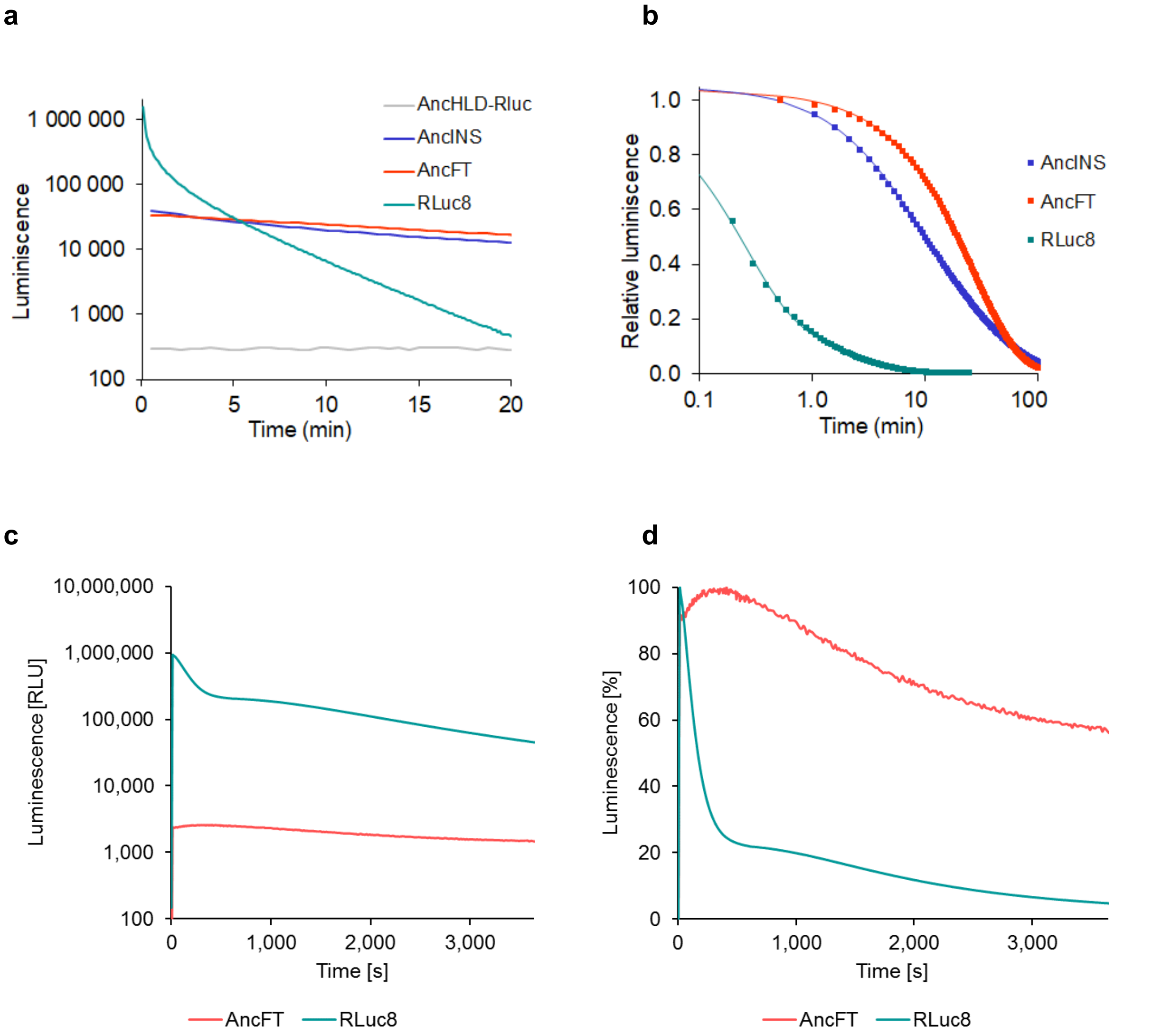

Supplementary Figure 14. Kinetics of bioluminescence activity. The initial bioluminescence signal steadiness **a** and the full decay kinetics **b** of the conversion of 2.2 µM coelenterazine by 50 nM of RLuc8 (teal), Anc$^{HLD\text{-}RLuc}$ (grey), AncINS (slate), and AncFT (salmon) purified from bacterial cultures. Note that the data in B are presented as relative values to initial luminescence; Anc$^{HLD\text{-}RLuc}$ is not plotted in relative scale due to low activity leading to signal scattering. All reactions were performed in 100 mM phosphate buffer at pH 7.5 and temperature 37 ^{o}C. Each trace was measured in 3 replicates and then averaged. Solid lines represent the best fit to the data. **c** Bioluminescence signal steadiness in absolute and **d** relative values in lysates from mammalian cells expressing AncFT and RLuc8. Activity measured with 25 µL of cell lysate with the addition of 225 µL 100 mM PBS pH=7.5 with 5 µM CTZ. Luminescence signal in lysates from mammalian cells expressing Anc$^{HLD\text{-}RLuc}$ and AncINS was not detectable under tested conditions. Experiments were performed in three independent replicates with consistent results.

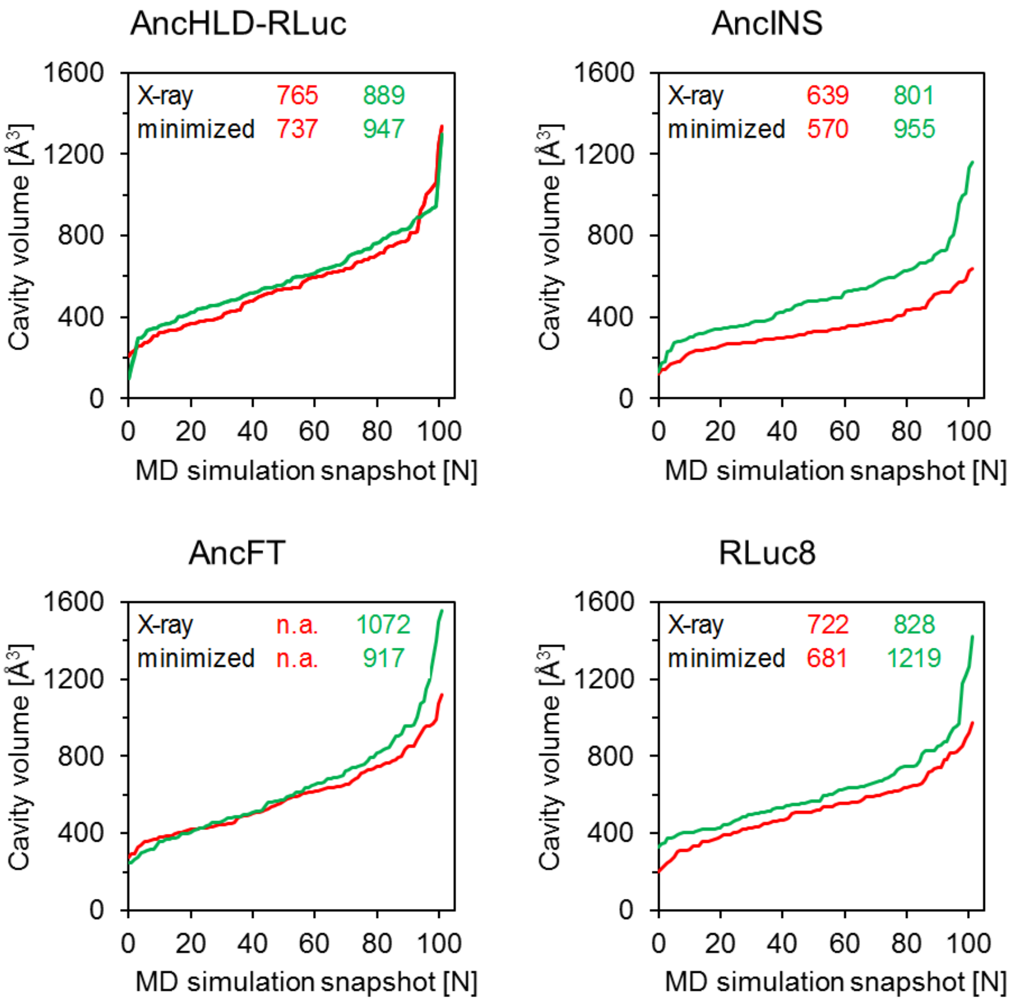

Figure 15. Analysis of the active site cavity volume of the 100 snapshots of molecular dynamics (MD) simulations starting from open and closed conformations. Red numbers and lines correspond to the cavity volume of a closed conformation (α4 helix in), and green numbers and lines correspond to the cavity volume of an open conformation (α4 helix out). AncFT has only one monomer in the asymmetric unit and therefore values for closed conformation for X-ray and minimized structures are not available. This analysis confirms that the catalytic properties of studied proteins do not correlate with the active site volumes.

Supplementary Table 8. Primers used for mutagenesis. Primer names for AncFT correspond to Supplementary Figure 14. FWD stands for forward and RVS for reverse primer.

PCR	Primer	Length (nucleotides)	Sequence 5´→3´
1	RLuc8-W121F-FWD1	43	TCATCTTTGTTGGTCATGATTTTGGTGCAGCACTGGCATTTCA
	RLuc8-W121F-RVS1	43	TGAAATGCCAGTGCTGCACCAAAATCATGACCAACAAAGATGA
2	RLuc-E144Q-FWD2	33	GCCATTGTTCACATGCAGAGCGTTGTGGATGTT
	RLuc-E144Q-RVS2	33	AACATCCACAACGCTCTGCATGTGAACAATGGC
1	AncFT-FWD1	20	TAATACGACTCACTATAGGG
	AncFT-RVS1	24	AACGCTTTCCATGTGAACAATACC
2	AncFT-FWD2	108	GGTATTGTTCACATGGAAAGCGTTGTGGATGTTATTGAAAGCTGGGATGAATGGCCTGATATCGAAGAAGATATTGCCCTGATTAAAAGCGAAGCCGGTGAAGAAATGGTTCTG
	AncFT-RVS2	19	GCTAGTTATTGCTCAGCGG

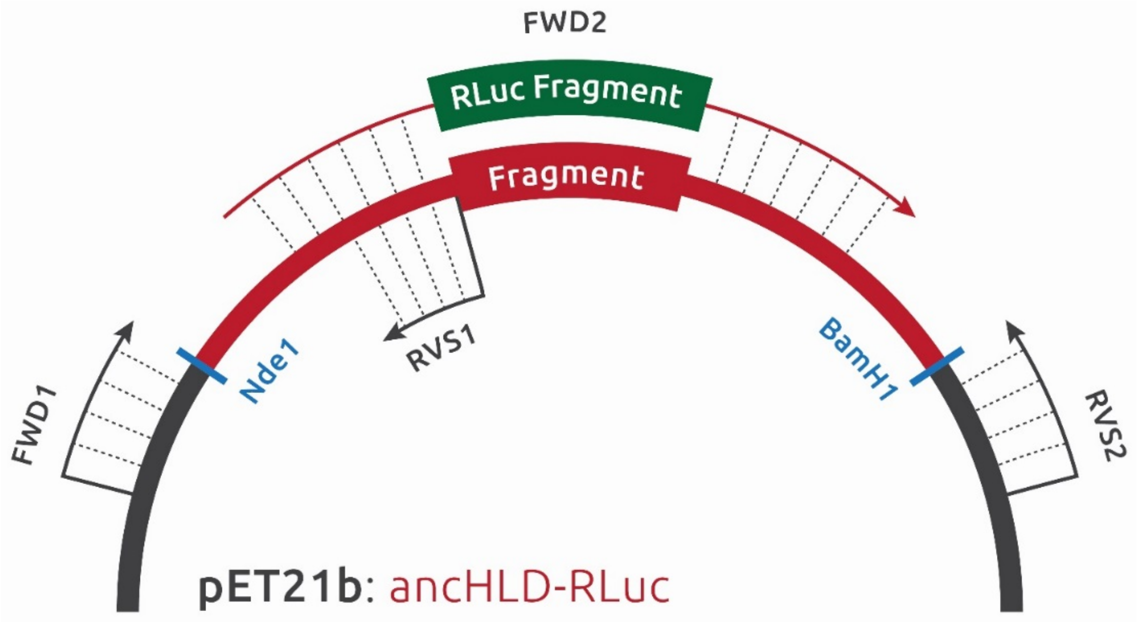

Supplementary Figure 16. Schematic representation of hybridization of primers designed for fragment transplantation from RLuc (green) onto Anc$^{HLD-RLuc}$ (red). Primer names (grey) correspond to AncFT primers in the Supplementary Table S8. FWD stands for forward and RVS for reverse primer. Restriction sites are shown in blue, plasmid pET21b is shown in grey.

Supplementary Note 7. AncINS PDB validation report.

Full wwPDB X-ray Structure Validation Report (i)

Aug 20, 2019 – 02:22 pm BST

PDB ID	:	6S6E
Title	:	Crystal structure of the engineered ancestor of haloalkane dehalogenases and Renilla luciferase (AncHLD-RLuc I161_F162PinsL)
Deposited on	:	2019-07-03
Resolution	:	2.00 Å(reported)

This is a Full wwPDB X-ray Structure Validation Report.

This report is produced by the wwPDB biocuration pipeline after annotation of the structure.

We welcome your comments at *validation@mail.wwpdb.org*
A user guide is available at
https://www.wwpdb.org/validation/2017/XrayValidationReportHelp
with specific help available everywhere you see the (i) symbol.

The following versions of software and data (see references (i)) were used in the production of this report:

MolProbity	:	4.02b-467
Xtriage (Phenix)	:	1.13
EDS	:	2.4
Percentile statistics	:	20171227.v01 (using entries in the PDB archive December 27th 2017)
Refmac	:	5.8.0158
CCP4	:	7.0 (Gargrove)
Ideal geometry (proteins)	:	Engh & Huber (2001)
Ideal geometry (DNA, RNA)	:	Parkinson et al. (1996)
Validation Pipeline (wwPDB-VP)	:	2.4

CONFIDENTIAL VALIDATION REPORT

1 Overall quality at a glance (i)

The following experimental techniques were used to determine the structure:
X-RAY DIFFRACTION

The reported resolution of this entry is 2.00 Å.

Percentile scores (ranging between 0-100) for global validation metrics of the entry are shown in the following graphic. The table shows the number of entries on which the scores are based.

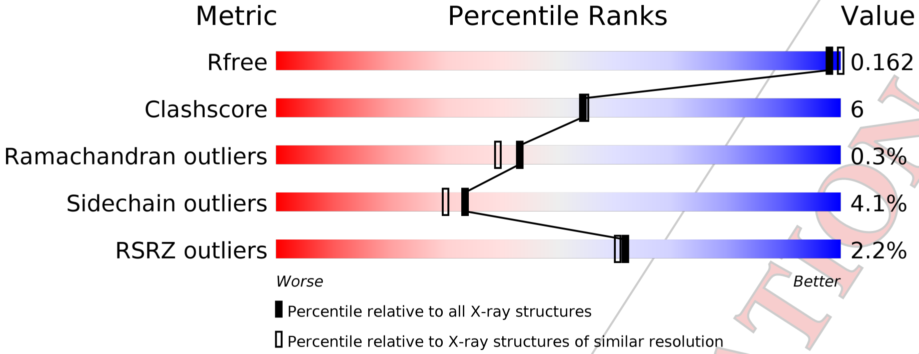

Metric	Whole archive (#Entries)	Similar resolution (#Entries, resolution range(Å))
R_{free}	111664	7193 (2.00-2.00)
Clashscore	122126	8267 (2.00-2.00)
Ramachandran outliers	120053	8166 (2.00-2.00)
Sidechain outliers	120020	8165 (2.00-2.00)
RSRZ outliers	108989	7011 (2.00-2.00)

The table below summarises the geometric issues observed across the polymeric chains and their fit to the electron density. The red, orange, yellow and green segments on the lower bar indicate the fraction of residues that contain outliers for >=3, 2, 1 and 0 types of geometric quality criteria. A grey segment represents the fraction of residues that are not modelled. The numeric value for each fraction is indicated below the corresponding segment, with a dot representing fractions <=5% The upper red bar (where present) indicates the fraction of residues that have poor fit to the electron density. The numeric value is given above the bar.

Mol	Chain	Length	Quality of chain
1	A	298	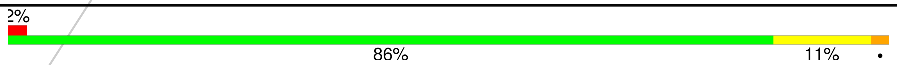
1	B	298	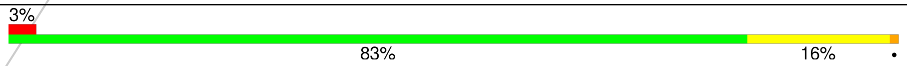

CONFIDENTIAL VALIDATION REPORT

2 Entry composition (i)

There are 2 unique types of molecules in this entry. The entry contains 4990 atoms, of which 0 are hydrogens and 0 are deuteriums.

In the tables below, the ZeroOcc column contains the number of atoms modelled with zero occupancy, the AltConf column contains the number of residues with at least one atom in alternate conformation and the Trace column contains the number of residues modelled with at most 2 atoms.

- Molecule 1 is a protein called Engineered ancestor of haloalkane dehalogenases and Renilla luciferase (AncHLD-RLuc I161_F162PinsL).

Mol	Chain	Residues	Atoms					ZeroOcc	AltConf	Trace
			Total	C	N	O	S			
1	A	297	2402	1538	408	446	10	0	0	0
1	B	297	2413	1545	410	448	10	0	1	0

- Molecule 2 is water.

Mol	Chain	Residues	Atoms		ZeroOcc	AltConf
			Total	O		
2	A	104	104	104	0	0
2	B	71	71	71	0	0

CONFIDENTIAL VALIDATION REPORT

3 Residue-property plots (i)

These plots are drawn for all protein, RNA and DNA chains in the entry. The first graphic for a chain summarises the proportions of the various outlier classes displayed in the second graphic. The second graphic shows the sequence view annotated by issues in geometryand electron density. Residues are color-coded according to the number of geometric quality criteria for which they contain at least one outlier: green = 0, yellow = 1, orange = 2 and red = 3 or more. A red dot above a residue indicates a poor fit to the electron density (RSRZ > 2). Stretches of 2 or more consecutive residues without any outlier are shown as a green connector. Residues present in the sample, but not in the model, are shown in grey.

• Molecule 1: Engineered ancestor of haloalkane dehalogenases and Renilla luciferase (AncHLD-RLuc I161_F162PinsL)

Chain A:

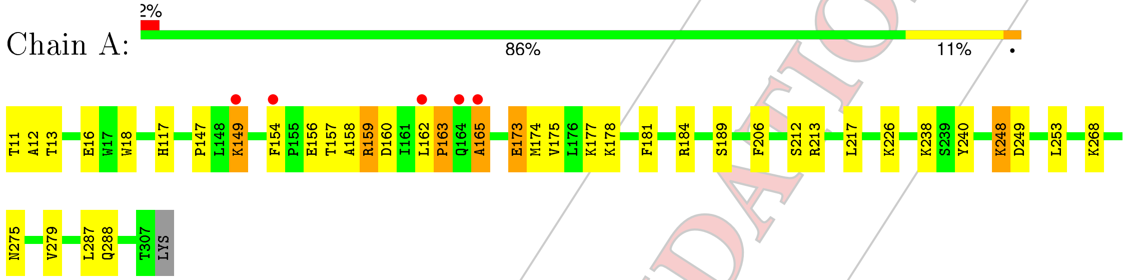

• Molecule 1: Engineered ancestor of haloalkane dehalogenases and Renilla luciferase (AncHLD-RLuc I161_F162PinsL)

Chain B:

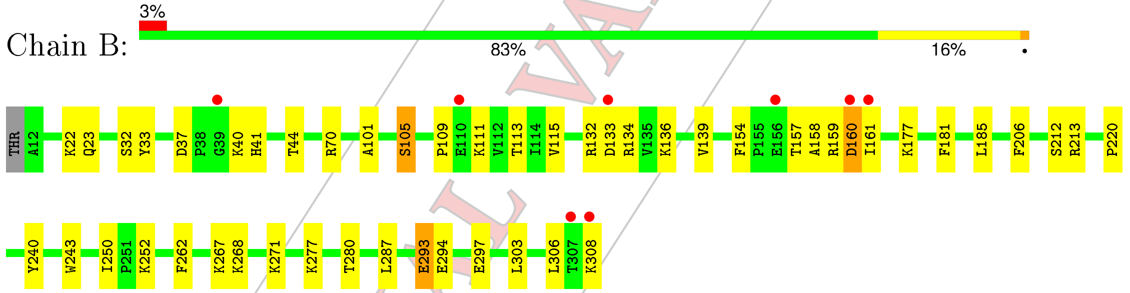

CONFIDENTIAL VALIDATION REPORT

4 Data and refinement statistics (i)

Property	Value	Source
Space group	P 21 21 21	Depositor
Cell constants<br>a, b, c, α, β, γ	44.71Å 84.17Å 160.22Å<br>90.00° 90.00° 90.00°	Depositor
Resolution (Å)	45.09 – 2.00<br>45.09 – 2.00	Depositor<br>EDS
% Data completeness<br>(in resolution range)	99.6 (45.09-2.00)<br>99.6 (45.09-2.00)	Depositor<br>EDS
R_{merge}	0.08	Depositor
R_{sym}	(Not available)	Depositor
$< I/\sigma(I) >$ [1]	2.35 (at 2.00Å)	Xtriage
Refinement program	PHENIX 1.14_3260	Depositor
R, R_{free}	0.170 , 0.214<br>0.173 , 0.162	Depositor<br>DCC
R_{free} test set	1984 reflections (4.77%)	wwPDB-VP
Wilson B-factor (Å^2)	32.1	Xtriage
Anisotropy	0.677	Xtriage
Bulk solvent k_{sol}(e/Å^3), B_{sol}(Å^2)	0.33 , 45.1	EDS
L-test for twinning[2]	$< \|L\| >= 0.49$, $< L^2 >= 0.33$	Xtriage
Estimated twinning fraction	No twinning to report.	Xtriage
F_o,F_c correlation	0.97	EDS
Total number of atoms	4990	wwPDB-VP
Average B, all atoms (Å^2)	43.0	wwPDB-VP

Xtriage's analysis on translational NCS is as follows: *The largest off-origin peak in the Patterson function is 6.60% of the height of the origin peak. No significant pseudotranslation is detected.*

[1] Intensities estimated from amplitudes.

[2] Theoretical values of $< |L| >$, $< L^2 >$ for acentric reflections are 0.5, 0.333 respectively for untwinned datasets, and 0.375, 0.2 for perfectly twinned datasets.

CONFIDENTIAL VALIDATION REPORT

5 Model quality (i)

5.1 Standard geometry (i)

The Z score for a bond length (or angle) is the number of standard deviations the observed value is removed from the expected value. A bond length (or angle) with $|Z| > 5$ is considered an outlier worth inspection. RMSZ is the root-mean-square of all Z scores of the bond lengths (or angles).

Mol	Chain	Bond lengths		Bond angles	
		RMSZ	#$|Z|$ >5	RMSZ	#$|Z|$ >5
1	A	0.43	0/2473	0.57	0/3358
1	B	0.44	0/2484	0.57	0/3371
All	All	0.43	0/4957	0.57	0/6729

Chiral center outliers are detected by calculating the chiral volume of a chiral center and verifying if the center is modelled as a planar moiety or with the opposite hand.A planarity outlier is detected by checking planarity of atoms in a peptide group, atoms in a mainchain group or atoms of a sidechain that are expected to be planar.

Mol	Chain	#Chirality outliers	#Planarity outliers
1	A	0	1

There are no bond length outliers.

There are no bond angle outliers.

There are no chirality outliers.

All (1) planarity outliers are listed below:

Mol	Chain	Res	Type	Group
1	A	159	ARG	Sidechain

5.2 Too-close contacts (i)

In the following table, the Non-H and H(model) columns list the number of non-hydrogen atoms and hydrogen atoms in the chain respectively. The H(added) column lists the number of hydrogen atoms added and optimized by MolProbity. The Clashes column lists the number of clashes within the asymmetric unit, whereas Symm-Clashes lists symmetry related clashes.

Mol	Chain	Non-H	H(model)	H(added)	Clashes	Symm-Clashes
1	A	2402	0	2341	26	0
1	B	2413	0	2352	33	0
2	A	104	0	0	2	0

Continued on next page...

CONFIDENTIAL VALIDATION REPORT

Continued from previous page...

Mol	Chain	Non-H	H(model)	H(added)	Clashes	Symm-Clashes
2	B	71	0	0	0	0
All	All	4990	0	4693	54	0

The all-atom clashscore is defined as the number of clashes found per 1000 atoms (including hydrogen atoms). The all-atom clashscore for this structure is 6.

All (54) close contacts within the same asymmetric unit are listed below, sorted by their clash magnitude.

Atom-1	Atom-2	Interatomic distance (Å)	Clash overlap (Å)
1:A:158:ALA:O	1:A:162:LEU:HD23	1.70	0.91
1:A:13:THR:HG23	1:A:16:GLU:H	1.42	0.82
1:B:157:THR:HB	1:B:161:ILE:HG13	1.67	0.76
1:A:158:ALA:O	1:A:162:LEU:CD2	2.37	0.72
1:A:156:GLU:HA	1:A:159:ARG:HH11	1.54	0.71
1:A:149:LYS:HA	1:A:149:LYS:HE3	1.81	0.62
1:A:12:ALA:HB2	2:A:450:HOH:O	1.99	0.61
1:B:293[A]:GLU:O	1:B:297:GLU:HG3	1.99	0.61
1:A:157:THR:HG22	1:B:280:THR:HG22	1.82	0.61
1:B:293[A]:GLU:CD	1:B:293[A]:GLU:H	2.04	0.60
1:B:293[A]:GLU:HG2	1:B:294:GLU:OE1	2.01	0.60
1:A:165:ALA:HB1	1:A:174:MET:SD	2.43	0.59
1:B:293[B]:GLU:O	1:B:297:GLU:HG3	2.01	0.59
1:B:41:HIS:HA	1:B:109:PRO:HG3	1.85	0.58
1:A:287:LEU:H	1:A:287:LEU:HD23	1.68	0.57
1:B:101:ALA:O	1:B:105:SER:OG	2.25	0.55
1:A:154:PHE:CD2	1:A:162:LEU:HD22	2.41	0.55
1:A:175:VAL:HG11	1:A:217:LEU:HB2	1.87	0.55
1:A:160:ASP:O	1:A:163:PRO:HD2	2.10	0.52
1:A:156:GLU:CA	1:A:159:ARG:HH11	2.22	0.52
1:B:44:THR:HG23	1:B:70:ARG:HD3	1.92	0.52
1:B:287:LEU:HD23	1:B:287:LEU:H	1.73	0.52
1:B:250:ILE:O	1:B:252:LYS:NZ	2.42	0.50
1:A:11:THR:HG22	2:A:450:HOH:O	2.11	0.49
1:B:157:THR:O	1:B:161:ILE:CG1	2.61	0.48
1:B:154:PHE:HB3	1:B:158:ALA:CB	2.45	0.47
1:B:268:LYS:O	1:B:271:LYS:HG3	2.14	0.47
1:A:253:LEU:HD11	1:A:279:VAL:HG13	1.97	0.47
1:A:173:GLU:OE1	1:A:178:LYS:HD2	2.16	0.46
1:B:113:THR:HG23	1:B:136:LYS:HB3	1.99	0.45
1:B:160:ASP:OD1	1:B:160:ASP:N	2.50	0.45
1:A:184:ARG:HH22	1:B:297:GLU:CD	2.20	0.44

Continued on next page...

CONFIDENTIAL VALIDATION REPORT

Continued from previous page...

Atom-1	Atom-2	Interatomic distance (Å)	Clash overlap (Å)
1:A:117:HIS:CG	1:A:288:GLN:HE22	2.36	0.44
1:B:303:LEU:HD23	1:B:306:LEU:HD12	1.98	0.44
1:B:161:ILE:O	1:B:185:LEU:HD12	2.17	0.44
1:B:267:LYS:O	1:B:271:LYS:HG2	2.19	0.43
1:B:22:LYS:HE2	1:B:33:TYR:OH	2.18	0.43
1:A:147:PRO:HB2	1:A:226:LYS:NZ	2.34	0.43
1:A:184:ARG:HD2	1:B:294:GLU:OE2	2.19	0.43
1:B:157:THR:O	1:B:161:ILE:HG13	2.20	0.42
1:B:111:LYS:HA	1:B:134:ARG:O	2.20	0.42
1:A:248:LYS:HE3	1:A:249:ASP:HB3	2.02	0.42
1:B:308:LYS:HE2	1:B:308:LYS:HB2	1.80	0.42
1:B:23:GLN:HG3	1:B:32:SER:HB2	2.01	0.42
1:B:206:PHE:HB3	1:B:213:ARG:HG2	2.00	0.42
1:A:147:PRO:HG2	1:A:238:LYS:HG3	2.01	0.42
1:A:184:ARG:HD2	1:B:294:GLU:CD	2.41	0.41
1:B:154:PHE:CE1	1:B:262:PHE:HE2	2.39	0.41
1:A:206:PHE:HB3	1:A:213:ARG:HG2	2.02	0.41
1:B:181:PHE:CE2	1:B:220:PRO:HG3	2.56	0.41
1:A:156:GLU:HA	1:A:159:ARG:HG3	2.01	0.41
1:B:115:VAL:HG22	1:B:139:VAL:HB	2.02	0.41
1:A:184:ARG:HD2	1:B:294:GLU:OE1	2.21	0.40
1:B:132:ARG:HG2	1:B:243:TRP:CE2	2.56	0.40

There are no symmetry-related clashes.

5.3 Torsion angles (i)

5.3.1 Protein backbone (i)

In the following table, the Percentiles column shows the percent Ramachandran outliers of the chain as a percentile score with respect to all X-ray entries followed by that with respect to entries of similar resolution.

The Analysed column shows the number of residues for which the backbone conformation was analysed, and the total number of residues.

Mol	Chain	Analysed	Favoured	Allowed	Outliers	Percentiles	
1	A	295/298 (99%)	279 (95%)	14 (5%)	2 (1%)	24	17
1	B	296/298 (99%)	284 (96%)	12 (4%)	0	100	100
All	All	591/596 (99%)	563 (95%)	26 (4%)	2 (0%)	43	39

CONFIDENTIAL VALIDATION REPORT

All (2) Ramachandran outliers are listed below:

Mol	Chain	Res	Type
1	A	163	PRO
1	A	165	ALA

5.3.2 Protein sidechains (i)

In the following table, the Percentiles column shows the percent sidechain outliers of the chain as a percentile score with respect to all X-ray entries followed by that with respect to entries of similar resolution.

The Analysed column shows the number of residues for which the sidechain conformation was analysed, and the total number of residues.

Mol	Chain	Analysed	Rotameric	Outliers	Percentiles	
1	A	266/267 (100%)	255 (96%)	11 (4%)	33	30
1	B	267/267 (100%)	255 (96%)	12 (4%)	30	26
All	All	533/534 (100%)	510 (96%)	23 (4%)	33	28

All (23) residues with a non-rotameric sidechain are listed below:

Mol	Chain	Res	Type
1	A	18	TRP
1	A	149	LYS
1	A	173	GLU
1	A	177	LYS
1	A	181	PHE
1	A	189	SER
1	A	212	SER
1	A	240	TYR
1	A	248	LYS
1	A	268	LYS
1	A	275	ASN
1	B	37	ASP
1	B	40	LYS
1	B	105	SER
1	B	133	ASP
1	B	159	ARG
1	B	160	ASP
1	B	177	LYS
1	B	212	SER
1	B	240	TYR
1	B	277	LYS

Continued on next page...

CONFIDENTIAL VALIDATION REPORT

Continued from previous page...

Mol	Chain	Res	Type
1	B	293[A]	GLU
1	B	293[B]	GLU

Some sidechains can be flipped to improve hydrogen bonding and reduce clashes. All (3) such sidechains are listed below:

Mol	Chain	Res	Type
1	A	164	GLN
1	A	275	ASN
1	A	304	ASN

5.3.3 RNA (i)

There are no RNA molecules in this entry.

5.4 Non-standard residues in protein, DNA, RNA chains (i)

There are no non-standard protein/DNA/RNA residues in this entry.

5.5 Carbohydrates (i)

There are no carbohydrates in this entry.

5.6 Ligand geometry (i)

There are no ligands in this entry.

5.7 Other polymers (i)

There are no such residues in this entry.

5.8 Polymer linkage issues (i)

There are no chain breaks in this entry.

CONFIDENTIAL VALIDATION REPORT

6 Fit of model and data (i)

6.1 Protein, DNA and RNA chains (i)

In the following table, the column labelled '#RSRZ> 2' contains the number (and percentage) of RSRZ outliers, followed by percent RSRZ outliers for the chain as percentile scores relative to all X-ray entries and entries of similar resolution. The OWAB column contains the minimum, median, 95th percentile and maximum values of the occupancy-weighted average B-factor per residue. The column labelled 'Q< 0.9' lists the number of (and percentage) of residues with an average occupancy less than 0.9.

Mol	Chain	Analysed	<RSRZ>	#RSRZ>2			OWAB(Å^2)	Q<0.9
1	A	297/298 (99%)	-0.21	5 (1%)	70	68	20, 36, 67, 96	0
1	B	297/298 (99%)	-0.02	8 (2%)	54	53	25, 43, 81, 101	0
All	All	594/596 (99%)	-0.12	13 (2%)	62	60	20, 38, 76, 101	0

All (13) RSRZ outliers are listed below:

Mol	Chain	Res	Type	RSRZ
1	A	164	GLN	4.4
1	B	307	THR	3.4
1	B	156	GLU	3.4
1	B	39	GLY	3.3
1	A	165	ALA	3.0
1	B	161	ILE	2.9
1	A	162	LEU	2.7
1	A	154	PHE	2.5
1	B	308	LYS	2.4
1	A	149	LYS	2.2
1	B	110	GLU	2.2
1	B	160	ASP	2.1
1	B	133	ASP	2.0

6.2 Non-standard residues in protein, DNA, RNA chains (i)

There are no non-standard protein/DNA/RNA residues in this entry.

6.3 Carbohydrates (i)

There are no carbohydrates in this entry.

CONFIDENTIAL VALIDATION REPORT

6.4 Ligands (i)

There are no ligands in this entry.

6.5 Other polymers (i)

There are no such residues in this entry.

CONFIDENTIAL VALIDATION REPORT

Supplementary Note 8. AncFT PDB validation report.

Full wwPDB X-ray Structure Validation Report (i)

Jul 11, 2019 – 04:46 pm BST

PDB ID	:	6S97
Title	:	Fragment transplantation onto a hyperstable ancestor of haloalkane dehalogenases and Renilla luciferase (Anc-FT)
Deposited on	:	2019-07-11
Resolution	:	1.95 Å(reported)

This is a Full wwPDB X-ray Structure Validation Report.

This report is produced by the wwPDB biocuration pipeline after annotation of the structure.

We welcome your comments at *validation@mail.wwpdb.org*
A user guide is available at
https://www.wwpdb.org/validation/2017/XrayValidationReportHelp
with specific help available everywhere you see the (i) symbol.

The following versions of software and data (see references (i)) were used in the production of this report:

MolProbity	:	4.02b-467
Xtriage (Phenix)	:	1.13
EDS	:	2.4
Percentile statistics	:	20171227.v01 (using entries in the PDB archive December 27th 2017)
Refmac	:	5.8.0158
CCP4	:	7.0 (Gargrove)
Ideal geometry (proteins)	:	Engh & Huber (2001)
Ideal geometry (DNA, RNA)	:	Parkinson et al. (1996)
Validation Pipeline (wwPDB-VP)	:	2.4

CONFIDENTIAL VALIDATION REPORT

1 Overall quality at a glance (i)

The following experimental techniques were used to determine the structure:
X-RAY DIFFRACTION

The reported resolution of this entry is 1.95 Å.

Percentile scores (ranging between 0-100) for global validation metrics of the entry are shown in the following graphic. The table shows the number of entries on which the scores are based.

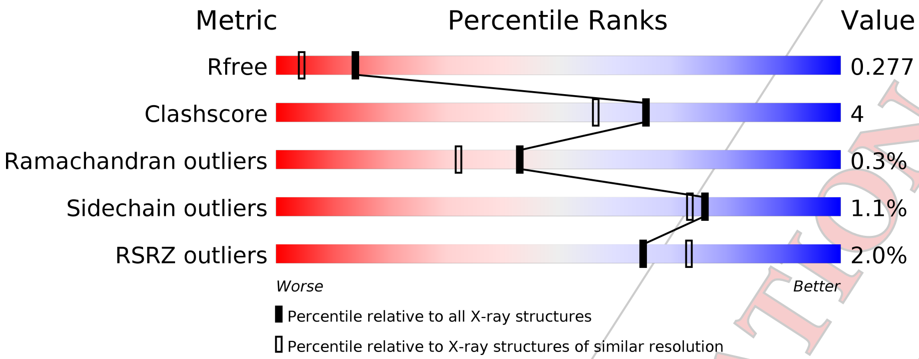

Metric	Whole archive (#Entries)	Similar resolution (#Entries, resolution range(Å))
R_{free}	111664	2220 (1.96-1.96)
Clashscore	122126	2333 (1.96-1.96)
Ramachandran outliers	120053	2314 (1.96-1.96)
Sidechain outliers	120020	2314 (1.96-1.96)
RSRZ outliers	108989	2174 (1.96-1.96)

The table below summarises the geometric issues observed across the polymeric chains and their fit to the electron density. The red, orange, yellow and green segments on the lower bar indicate the fraction of residues that contain outliers for >=3, 2, 1 and 0 types of geometric quality criteria. A grey segment represents the fraction of residues that are not modelled. The numeric value for each fraction is indicated below the corresponding segment, with a dot representing fractions <=5% The upper red bar (where present) indicates the fraction of residues that have poor fit to the electron density. The numeric value is given above the bar.

Mol	Chain	Length	Quality of chain
1	A	294	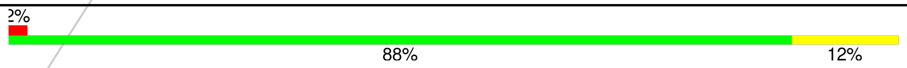

CONFIDENTIAL VALIDATION REPORT

2 Entry composition (i)

There are 2 unique types of molecules in this entry. The entry contains 2545 atoms, of which 0 are hydrogens and 0 are deuteriums.

In the tables below, the ZeroOcc column contains the number of atoms modelled with zero occupancy, the AltConf column contains the number of residues with at least one atom in alternate conformation and the Trace column contains the number of residues modelled with at most 2 atoms.

- Molecule 1 is a protein called Fragment transplantation onto hyperstable ancestor of haloalkane dehalogenases and Renilla luciferase (Anc-FT).

Mol	Chain	Residues	Atoms					ZeroOcc	AltConf	Trace
			Total	C	N	O	S			
1	A	294	2405	1539	401	455	10	0	2	0

- Molecule 2 is water.

Mol	Chain	Residues	Atoms		ZeroOcc	AltConf
			Total	O		
2	A	140	140	140	0	0

CONFIDENTIAL VALIDATION REPORT

3 Residue-property plots (i)

These plots are drawn for all protein, RNA and DNA chains in the entry. The first graphic for a chain summarises the proportions of the various outlier classes displayed in the second graphic. The second graphic shows the sequence view annotated by issues in geometryand electron density. Residues are color-coded according to the number of geometric quality criteria for which they contain at least one outlier: green = 0, yellow = 1, orange = 2 and red = 3 or more. A red dot above a residue indicates a poor fit to the electron density (RSRZ > 2). Stretches of 2 or more consecutive residues without any outlier are shown as a green connector. Residues present in the sample, but not in the model, are shown in grey.

• Molecule 1: Fragment transplantation onto hyperstable ancestor of haloalkane dehalogenases and Renilla luciferase (Anc-FT)

Chain A:

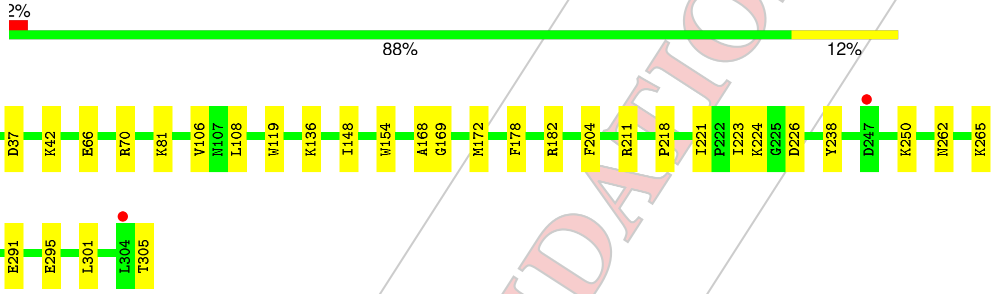

CONFIDENTIAL VALIDATION REPORT

4 Data and refinement statistics (i)

Property	Value	Source
Space group	P 31 2 1	Depositor
Cell constants<br>a, b, c, α, β, γ	87.56Å 87.56Å 102.12Å<br>90.00° 90.00° 120.00°	Depositor
Resolution (Å)	43.78 – 1.95<br>43.78 – 1.95	Depositor<br>EDS
% Data completeness<br>(in resolution range)	99.9 (43.78-1.95)<br>99.9 (43.78-1.95)	Depositor<br>EDS
R_{merge}	0.14	Depositor
R_{sym}	(Not available)	Depositor
$< I/\sigma(I) >$ [1]	1.73 (at 1.95Å)	Xtriage
Refinement program	PHENIX 1.14_3260	Depositor
R, R_{free}	0.291 , 0.330<br>0.294 , 0.277	Depositor<br>DCC
R_{free} test set	1694 reflections (5.08%)	wwPDB-VP
Wilson B-factor (Å^2)	26.9	Xtriage
Anisotropy	0.475	Xtriage
Bulk solvent k_{sol}(e/Å^3), B_{sol}(Å^2)	0.34 , 58.5	EDS
L-test for twinning[2]	$< \|L\| > = 0.44$, $< L^2 > = 0.26$	Xtriage
Estimated twinning fraction	0.058 for -h,-k,l	Xtriage
F_o,F_c correlation	0.87	EDS
Total number of atoms	2545	wwPDB-VP
Average B, all atoms (Å^2)	28.0	wwPDB-VP

Xtriage's analysis on translational NCS is as follows: *The largest off-origin peak in the Patterson function is 14.73% of the height of the origin peak. No significant pseudotranslation is detected.*

[1] Intensities estimated from amplitudes.
[2] Theoretical values of $< |L| >$, $< L^2 >$ for acentric reflections are 0.5, 0.333 respectively for untwinned datasets, and 0.375, 0.2 for perfectly twinned datasets.

CONFIDENTIAL VALIDATION REPORT

5 Model quality (i)

5.1 Standard geometry (i)

The Z score for a bond length (or angle) is the number of standard deviations the observed value is removed from the expected value. A bond length (or angle) with $|Z| > 5$ is considered an outlier worth inspection. RMSZ is the root-mean-square of all Z scores of the bond lengths (or angles).

Mol	Chain	Bond lengths		Bond angles	
		RMSZ	#$|Z|$ >5	RMSZ	#$|Z|$ >5
1	A	0.40	0/2475	0.55	0/3363

There are no bond length outliers.

There are no bond angle outliers.

There are no chirality outliers.

There are no planarity outliers.

5.2 Too-close contacts (i)

In the following table, the Non-H and H(model) columns list the number of non-hydrogen atoms and hydrogen atoms in the chain respectively. The H(added) column lists the number of hydrogen atoms added and optimized by MolProbity. The Clashes column lists the number of clashes within the asymmetric unit, whereas Symm-Clashes lists symmetry related clashes.

Mol	Chain	Non-H	H(model)	H(added)	Clashes	Symm-Clashes
1	A	2405	0	2320	21	0
2	A	140	0	0	2	0
All	All	2545	0	2320	21	0

The all-atom clashscore is defined as the number of clashes found per 1000 atoms (including hydrogen atoms). The all-atom clashscore for this structure is 4.

All (21) close contacts within the same asymmetric unit are listed below, sorted by their clash magnitude.

Atom-1	Atom-2	Interatomic distance (Å)	Clash overlap (Å)
1:A:273:ASN:ND2	2:A:402:HOH:O	2.23	0.71
1:A:291:GLU:O	1:A:295:GLU:HG3	2.04	0.58
1:A:223:ILE:HB	1:A:226:ASP:HB2	1.90	0.53
1:A:262:ASN:HA	1:A:265:LYS:HE3	1.90	0.53

Continued on next page...

CONFIDENTIAL VALIDATION REPORT

Continued from previous page...

Atom-1	Atom-2	Interatomic distance (Å)	Clash overlap (Å)
1:A:119:TRP:HZ2	1:A:221:ILE:HD13	1.76	0.51
1:A:169:GLY:HA2	1:A:172:MET:HE2	1.94	0.50
1:A:178:PHE:CD1	1:A:182:ARG:HD3	2.48	0.49
1:A:204:PHE:HB3	1:A:211:ARG:HG2	1.94	0.49
1:A:148:ILE:HG23	1:A:154:TRP:CD1	2.50	0.47
1:A:178:PHE:HD1	1:A:182:ARG:HD3	1.80	0.46
1:A:119:TRP:CZ2	1:A:221:ILE:HD13	2.50	0.46
1:A:168:ALA:O	1:A:172:MET:HG3	2.15	0.46
1:A:23:GLN:NE2	2:A:412:HOH:O	2.50	0.45
1:A:42:LYS:HE2	1:A:70:ARG:N	2.32	0.45
1:A:218:PRO:O	1:A:221:ILE:HG12	2.18	0.44
1:A:34:TYR:OH	1:A:66:GLU:OE2	2.20	0.43
1:A:81:LYS:HD3	1:A:81:LYS:HA	1.86	0.43
1:A:266:LYS:O	1:A:269:LYS:HG2	2.19	0.43
1:A:301:LEU:O	1:A:305:THR:HG23	2.20	0.42
1:A:106:VAL:HG23	1:A:108:LEU:HG	2.01	0.41
1:A:250:LYS:HG3	1:A:250:LYS:HZ3	1.59	0.40

There are no symmetry-related clashes.

5.3 Torsion angles (i)

5.3.1 Protein backbone (i)

In the following table, the Percentiles column shows the percent Ramachandran outliers of the chain as a percentile score with respect to all X-ray entries followed by that with respect to entries of similar resolution.

The Analysed column shows the number of residues for which the backbone conformation was analysed, and the total number of residues.

Mol	Chain	Analysed	Favoured	Allowed	Outliers	Percentiles
1	A	294/294 (100%)	282 (96%)	11 (4%)	1 (0%)	43 32

All (1) Ramachandran outliers are listed below:

Mol	Chain	Res	Type
1	A	224	LYS

CONFIDENTIAL VALIDATION REPORT

5.3.2 Protein sidechains (i)

In the following table, the Percentiles column shows the percent sidechain outliers of the chain as a percentile score with respect to all X-ray entries followed by that with respect to entries of similar resolution.

The Analysed column shows the number of residues for which the sidechain conformation was analysed, and the total number of residues.

Mol	Chain	Analysed	Rotameric	Outliers	Percentiles	
1	A	267/265 (101%)	263 (98%)	4 (2%)	67	63

All (4) residues with a non-rotameric sidechain are listed below:

Mol	Chain	Res	Type
1	A	37[A]	ASP
1	A	37[B]	ASP
1	A	136	LYS
1	A	238	TYR

Some sidechains can be flipped to improve hydrogen bonding and reduce clashes. All (2) such sidechains are listed below:

Mol	Chain	Res	Type
1	A	23	GLN
1	A	262	ASN

5.3.3 RNA (i)

There are no RNA molecules in this entry.

5.4 Non-standard residues in protein, DNA, RNA chains (i)

There are no non-standard protein/DNA/RNA residues in this entry.

5.5 Carbohydrates (i)

There are no carbohydrates in this entry.

5.6 Ligand geometry (i)

There are no ligands in this entry.

CONFIDENTIAL VALIDATION REPORT

5.7 Other polymers (i)

There are no such residues in this entry.

5.8 Polymer linkage issues (i)

There are no chain breaks in this entry.

CONFIDENTIAL VALIDATION REPORT

6 Fit of model and data (i)

6.1 Protein, DNA and RNA chains (i)

In the following table, the column labelled '#RSRZ> 2' contains the number (and percentage) of RSRZ outliers, followed by percent RSRZ outliers for the chain as percentile scores relative to all X-ray entries and entries of similar resolution. The OWAB column contains the minimum, median, 95th percentile and maximum values of the occupancy-weighted average B-factor per residue. The column labelled 'Q< 0.9' lists the number of (and percentage) of residues with an average occupancy less than 0.9.

Mol	Chain	Analysed	<RSRZ>	#RSRZ>2	OWAB(Å^2)	Q<0.9
1	A	294/294 (100%)	0.12	6 (2%) 65 73	15, 27, 42, 51	0

All (6) RSRZ outliers are listed below:

Mol	Chain	Res	Type	RSRZ
1	A	269	LYS	3.7
1	A	13	THR	3.0
1	A	12	ALA	2.3
1	A	247	ASP	2.2
1	A	304	LEU	2.1
1	A	270	ASN	2.0

6.2 Non-standard residues in protein, DNA, RNA chains (i)

There are no non-standard protein/DNA/RNA residues in this entry.

6.3 Carbohydrates (i)

There are no carbohydrates in this entry.

6.4 Ligands (i)

There are no ligands in this entry.

6.5 Other polymers (i)

There are no such residues in this entry.

CONFIDENTIAL VALIDATION REPORT

Supplementary Note 9. RLuc8-W121F/E144Q PDB validation report.

Preliminary Full wwPDB X-ray Structure Validation Report (i)

Apr 10, 2020 – 03:27 PM BST

Deposition ID : D_1292107896

This is a Preliminary Full wwPDB X-ray Structure Validation Report.

This report is produced by the wwPDB Deposition System during initial deposition but before annotation of the structure.

We welcome your comments at *validation@mail.wwpdb.org*
A user guide is available at
https://www.wwpdb.org/validation/2017/XrayValidationReportHelp
with specific help available everywhere you see the (i) symbol.

PRELIMINARY VALIDATION REPORT

The following versions of software and data (see references (i)) were used in the production of this report:

MolProbity	:	4.02b-467
Mogul	:	1.8.5 (274361), CSD as541be (2020)
Xtriage (Phenix)	:	1.13
EDS	:	2.10.1
buster-report	:	1.1.7 (2018)
Percentile statistics	:	20171227.v01 (using entries in the PDB archive December 27th 2017)
Refmac	:	5.8.0158
CCP4	:	7.0.044 (Gargrove)
Ideal geometry (proteins)	:	Engh & Huber (2001)
Ideal geometry (DNA, RNA)	:	Parkinson et al. (1996)
Validation Pipeline (wwPDB-VP)	:	2.10.1

1 Overall quality at a glance (i)

The following experimental techniques were used to determine the structure:
X-RAY DIFFRACTION

The reported resolution of this entry is 1.90 Å.

Percentile scores (ranging between 0-100) for global validation metrics of the entry are shown in the following graphic. The table shows the number of entries on which the scores are based.

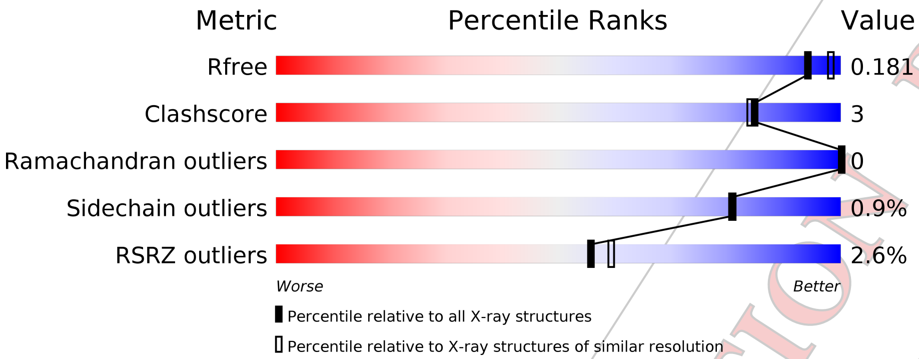

Metric	Whole archive (#Entries)	Similar resolution (#Entries, resolution range(Å))
R_{free}	111664	5502 (1.90-1.90)
Clashscore	122126	6115 (1.90-1.90)
Ramachandran outliers	120053	6048 (1.90-1.90)
Sidechain outliers	120020	6048 (1.90-1.90)
RSRZ outliers	108989	5379 (1.90-1.90)

The table below summarises the geometric issues observed across the polymeric chains and their fit to the electron density. The red, orange, yellow and green segments on the lower bar indicate the fraction of residues that contain outliers for >=3, 2, 1 and 0 types of geometric quality criteria respectively. A grey segment represents the fraction of residues that are not modelled. The numeric value for each fraction is indicated below the corresponding segment, with a dot representing fractions <=5% The upper red bar (where present) indicates the fraction of residues that have poor fit to the electron density. The numeric value is given above the bar.

Mol	Chain	Length	Quality of chain
1	A	317	3% / 92% 5% •
1	B	317	2% / 89% 8% •

PRELIMINARY VALIDATION REPORT

2 Entry composition (i)

There are 6 unique types of molecules in this entry. The entry contains 5673 atoms, of which 0 are hydrogens and 0 are deuteriums.

In the tables below, the ZeroOcc column contains the number of atoms modelled with zero occupancy, the AltConf column contains the number of residues with at least one atom in alternate conformation and the Trace column contains the number of residues modelled with at most 2 atoms.

- Molecule 1 is a protein.

Mol	Chain	Residues	Atoms					ZeroOcc	AltConf	Trace
			Total	C	N	O	S			
1	A	310	2552	1652	428	463	9	0	2	0
1	B	309	2540	1644	428	459	9	0	1	0

- Molecule 2 is N-[3-BENZYL-5-(4-HYDROXYPHENYL)PYRAZIN-2-YL]-2-(4-HYDROXY PHENYL)ACETAMIDE (three-letter code: CEI) (formula: $C_{25}H_{21}N_3O_3$).

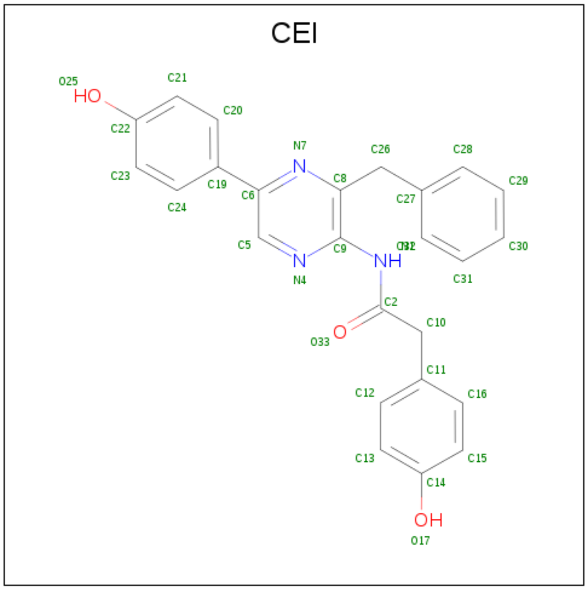

Mol	Chain	Residues	Atoms				ZeroOcc	AltConf
			Total	C	N	O		
2	A	1	31	25	3	3	0	0
2	B	1	31	25	3	3	0	0

- Molecule 3 is GLYCEROL (three-letter code: GOL) (formula: $C_3H_8O_3$).

PRELIMINARY VALIDATION REPORT

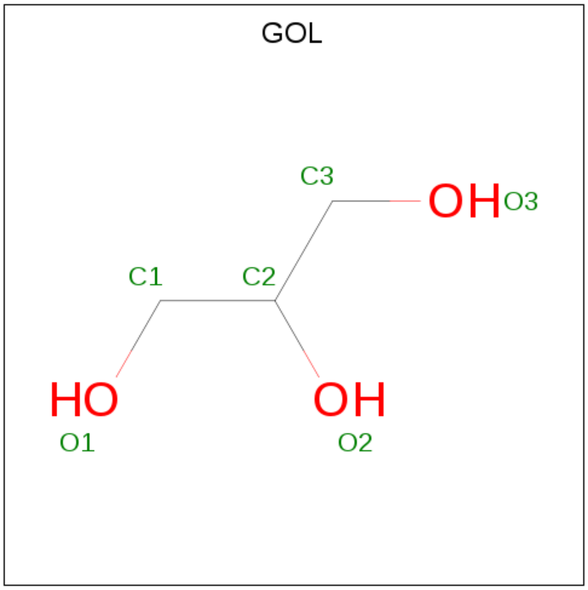

Mol	Chain	Residues	Atoms			ZeroOcc	AltConf
			Total	C	O		
3	A	1	6	3	3	0	0

- Molecule 4 is ACETATE ION (three-letter code: ACT) (formula: $C_2H_3O_2$).

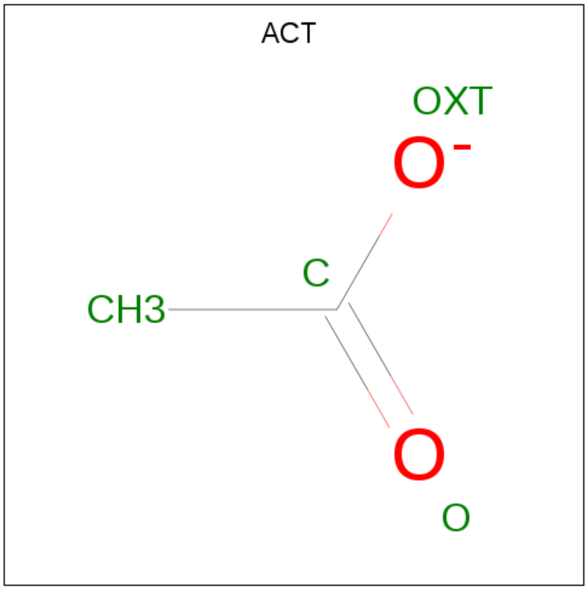

Mol	Chain	Residues	Atoms			ZeroOcc	AltConf
			Total	C	O		
4	A	1	4	2	2	0	0

- Molecule 5 is POTASSIUM ION (three-letter code: K) (formula: K).

PRELIMINARY VALIDATION REPORT

Mol	Chain	Residues	Atoms		ZeroOcc	AltConf
			Total	K		
5	B	1	1	1	0	0

- Molecule 6 is water.

Mol	Chain	Residues	Atoms		ZeroOcc	AltConf
			Total	O		
6	D	507	508	508	0	1

PRELIMINARY VALIDATION REPORT

3 Residue-property plots (i)

These plots are drawn for all protein, RNA and DNA chains in the entry. The first graphic for a chain summarises the proportions of the various outlier classes displayed in the second graphic. The second graphic shows the sequence view annotated by issues in geometry and electron density. Residues are color-coded according to the number of geometric quality criteria for which they contain at least one outlier: green = 0, yellow = 1, orange = 2 and red = 3 or more. A red dot above a residue indicates a poor fit to the electron density (RSRZ > 2). Stretches of 2 or more consecutive residues without any outlier are shown as a green connector. Residues present in the sample, but not in the model, are shown in grey.

- Molecule 1:

Chain A:
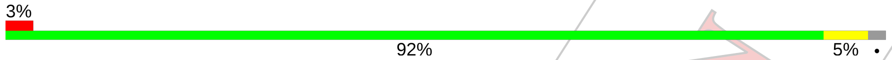

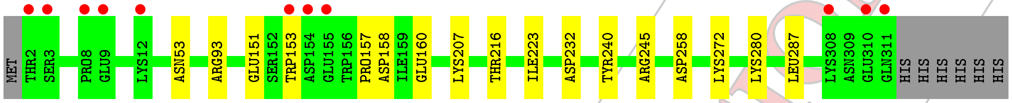

- Molecule 1:

Chain B:
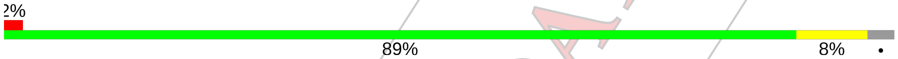

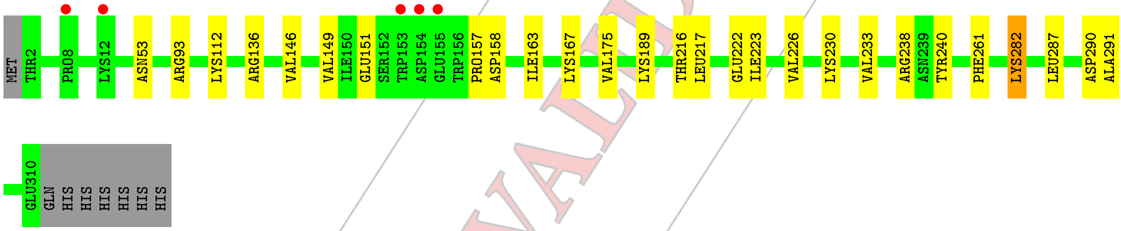

PRELIMINARY VALIDATION REPORT

4 Data and refinement statistics (i)

Property	Value	Source
Space group	I 2 2 2	Depositor
Cell constants<br>a, b, c, α, β, γ	112.77Å 131.08Å 142.03Å<br>90.00° 90.00° 90.00°	Depositor
Resolution (Å)	44.53 – 1.90<br>44.53 – 1.90	Depositor<br>EDS
% Data completeness<br>(in resolution range)	99.9 (44.53-1.90)<br>99.9 (44.53-1.90)	Depositor<br>EDS
R_{merge}	0.12	Depositor
R_{sym}	(Not available)	Depositor
$< I/\sigma(I) >$ [1]	1.08 (at 1.89Å)	Xtriage
Refinement program	PHENIX	Depositor
R, R_{free}	0.172 , 0.196<br>0.173 , 0.181	Depositor<br>DCC
R_{free} test set	4032 reflections (4.88%)	wwPDB-VP
Wilson B-factor (Å^2)	31.3	Xtriage
Anisotropy	0.301	Xtriage
Bulk solvent k_{sol}(e/Å^3), B_{sol}(Å^2)	0.37 , 44.5	EDS
L-test for twinning[2]	$< \|L\| >= 0.49$, $< L^2 > = 0.32$	Xtriage
Estimated twinning fraction	No twinning to report.	Xtriage
F_o,F_c correlation	0.97	EDS
Total number of atoms	5673	wwPDB-VP
Average B, all atoms (Å^2)	34.0	wwPDB-VP

Xtriage's analysis on translational NCS is as follows: *The largest off-origin peak in the Patterson function is 3.90% of the height of the origin peak. No significant pseudotranslation is detected.*

[1] Intensities estimated from amplitudes.
[2] Theoretical values of $< |L| >$, $< L^2 >$ for acentric reflections are 0.5, 0.333 respectively for untwinned datasets, and 0.375, 0.2 for perfectly twinned datasets.

PRELIMINARY VALIDATION REPORT

5 Model quality (i)

5.1 Standard geometry (i)

Bond lengths and bond angles in the following residue types are not validated in this section: GOL, K, CEI, ACT

The Z score for a bond length (or angle) is the number of standard deviations the observed value is removed from the expected value. A bond length (or angle) with $|Z| > 5$ is considered an outlier worth inspection. RMSZ is the root-mean-square of all Z scores of the bond lengths (or angles).

Mol	Chain	Bond lengths		Bond angles	
		RMSZ	#|Z| >5	RMSZ	#|Z| >5
1	A	0.37	0/2623	0.54	0/3550
1	B	0.39	0/2611	0.54	0/3533
All	All	0.38	0/5234	0.54	0/7083

There are no bond length outliers.

There are no bond angle outliers.

There are no chirality outliers.

There are no planarity outliers.

5.2 Too-close contacts (i)

In the following table, the Non-H and H(model) columns list the number of non-hydrogen atoms and hydrogen atoms in the chain respectively. The H(added) column lists the number of hydrogen atoms added and optimized by MolProbity. The Clashes column lists the number of clashes within the asymmetric unit, whereas Symm-Clashes lists symmetry related clashes.

Mol	Chain	Non-H	H(model)	H(added)	Clashes	Symm-Clashes
1	A	2552	0	2517	10	0
1	B	2540	0	2507	17	0
2	A	31	0	21	1	0
2	B	31	0	21	3	0
3	A	6	0	8	0	0
4	A	4	0	3	0	0
5	B	1	0	0	0	0
6	D	508	0	0	1	0
All	All	5673	0	5077	26	0

The all-atom clashscore is defined as the number of clashes found per 1000 atoms (including hydrogen atoms). The all-atom clashscore for this structure is 3.

PRELIMINARY VALIDATION REPORT

All (26) close contacts within the same asymmetric unit are listed below, sorted by their clash magnitude.

Atom-1	Atom-2	Interatomic distance (Å)	Clash overlap (Å)
1:B:151:GLU:HG3	1:B:226:VAL:HG21	1.67	0.76
1:B:282:LYS:HD3	1:B:291:ALA:HB2	1.78	0.64
1:B:93:ARG:HD2	1:B:222:GLU:OE2	2.04	0.57
1:A:158:ASP:HB3	1:B:157:PRO:HB3	1.85	0.57
1:B:230:LYS:HB2	1:B:233:VAL:HG12	1.88	0.55
1:A:223:ILE:HG13	2:A:350:CEI:H102	1.90	0.54
1:A:153:TRP:HZ2	1:A:160:GLU:HG3	1.76	0.50
1:B:282:LYS:HE2	1:B:290:ASP:O	2.13	0.49
1:B:163:ILE:O	1:B:167:LYS:HG3	2.14	0.48
1:B:282:LYS:HD2	6:D:261:HOH:O	2.14	0.48
1:B:287:LEU:HD23	1:B:287:LEU:H	1.78	0.48
1:B:112:LYS:HE2	1:B:136:ARG:HD2	1.98	0.46
1:A:287:LEU:H	1:A:287:LEU:HD23	1.81	0.45
1:B:175:VAL:HG11	1:B:217:LEU:HB2	2.00	0.44
1:A:245:ARG:CZ	1:A:272:LYS:HD3	2.47	0.43
2:B:350:CEI:H12	2:B:350:CEI:C31	2.48	0.43
1:A:93:ARG:NH2	1:A:232:ASP:OD2	2.46	0.42
1:B:149:VAL:HG11	1:B:238[A]:ARG:HG3	2.00	0.42
1:B:189:LYS:HE3	1:B:261:PHE:CE1	2.54	0.42
1:A:157:PRO:HB3	1:B:158:ASP:HB3	2.01	0.42
1:A:258:ASP:HB2	1:A:280:LYS:HD2	2.03	0.41
1:A:53:ASN:OD1	1:A:216:THR:HA	2.20	0.41
1:B:146:VAL:HA	2:B:350:CEI:C13	2.51	0.41
1:B:53:ASN:OD1	1:B:216:THR:HA	2.20	0.41
1:B:223:ILE:HG13	2:B:350:CEI:H102	2.01	0.41
1:A:153:TRP:CZ2	1:A:160:GLU:HG3	2.56	0.40

There are no symmetry-related clashes.

5.3 Torsion angles (i)

5.3.1 Protein backbone (i)

In the following table, the Percentiles column shows the percent Ramachandran outliers of the chain as a percentile score with respect to all X-ray entries followed by that with respect to entries of similar resolution.

The Analysed column shows the number of residues for which the backbone conformation was analysed, and the total number of residues.

PRELIMINARY VALIDATION REPORT

Mol	Chain	Analysed	Favoured	Allowed	Outliers	Percentiles	
1	A	310/317 (98%)	299 (96%)	11 (4%)	0	100	100
1	B	308/317 (97%)	298 (97%)	10 (3%)	0	100	100
All	All	618/634 (98%)	597 (97%)	21 (3%)	0	100	100

There are no Ramachandran outliers to report.

5.3.2 Protein sidechains (i)

In the following table, the Percentiles column shows the percent sidechain outliers of the chain as a percentile score with respect to all X-ray entries followed by that with respect to entries of similar resolution.

The Analysed column shows the number of residues for which the sidechain conformation was analysed, and the total number of residues.

Mol	Chain	Analysed	Rotameric	Outliers	Percentiles	
1	A	276/281 (98%)	273 (99%)	3 (1%)	76	75
1	B	274/281 (98%)	272 (99%)	2 (1%)	85	86
All	All	550/562 (98%)	545 (99%)	5 (1%)	81	81

All (5) residues with a non-rotameric sidechain are listed below:

Mol	Chain	Res	Type
1	A	151	GLU
1	A	207	LYS
1	A	240	TYR
1	B	240	TYR
1	B	282	LYS

Some sidechains can be flipped to improve hydrogen bonding and reduce clashes. All (1) such sidechains are listed below:

Mol	Chain	Res	Type
1	A	309	ASN

5.3.3 RNA (i)

There are no RNA molecules in this entry.

PRELIMINARY VALIDATION REPORT

5.4 Non-standard residues in protein, DNA, RNA chains (i)

There are no non-standard protein/DNA/RNA residues in this entry.

5.5 Carbohydrates (i)

There are no carbohydrates in this entry.

5.6 Ligand geometry (i)

Of 5 ligands modelled in this entry, 1 is monoatomic - leaving 4 for Mogul analysis.

In the following table, the Counts columns list the number of bonds (or angles) for which Mogul statistics could be retrieved, the number of bonds (or angles) that are observed in the model and the number of bonds (or angles) that are defined in the Chemical Component Dictionary. The Link column lists molecule types, if any, to which the group is linked. The Z score for a bond length (or angle) is the number of standard deviations the observed value is removed from the expected value. A bond length (or angle) with $|Z| > 2$ is considered an outlier worth inspection. RMSZ is the root-mean-square of all Z scores of the bond lengths (or angles).

Mol	Type	Chain	Res	Link	Bond lengths			Bond angles		
					Counts	RMSZ	$\#\|Z\| > 2$	Counts	RMSZ	$\#\|Z\| > 2$
3	GOL	A	400	-	5,5,5	0.55	0	5,5,5	0.34	0
4	ACT	A	500	-	1,3,3	4.07	1 (100%)	0,3,3	0.00	-
2	CEI	B	350	-	33,34,34	1.43	4 (12%)	43,46,46	1.36	7 (16%)
2	CEI	A	350	-	33,34,34	1.48	5 (15%)	43,46,46	1.30	4 (9%)

In the following table, the Chirals column lists the number of chiral outliers, the number of chiral centers analysed, the number of these observed in the model and the number defined in the Chemical Component Dictionary. Similar counts are reported in the Torsion and Rings columns. '-' means no outliers of that kind were identified.

Mol	Type	Chain	Res	Link	Chirals	Torsions	Rings
3	GOL	A	400	-	-	0/4/4/4	-
2	CEI	B	350	-	-	3/16/16/16	0/4/4/4
2	CEI	A	350	-	-	4/16/16/16	0/4/4/4

All (10) bond length outliers are listed below:

Mol	Chain	Res	Type	Atoms	Z	Observed(Å)	Ideal(Å)
2	A	350	CEI	C2-N1	4.52	1.45	1.35
2	B	350	CEI	C2-N1	4.50	1.45	1.35

Continued on next page...

PRELIMINARY VALIDATION REPORT

Continued from previous page...

Mol	Chain	Res	Type	Atoms	Z	Observed(Å)	Ideal(Å)
4	A	500	ACT	CH3-C	4.07	1.53	1.48
2	B	350	CEI	C9-N1	3.74	1.46	1.39
2	A	350	CEI	C9-N1	3.40	1.46	1.39
2	A	350	CEI	C26-C8	3.36	1.54	1.51
2	A	350	CEI	C19-C6	3.21	1.54	1.48
2	B	350	CEI	C26-C8	2.88	1.53	1.51
2	B	350	CEI	C19-C6	2.87	1.53	1.48
2	A	350	CEI	O33-C2	-2.14	1.18	1.23

All (11) bond angle outliers are listed below:

Mol	Chain	Res	Type	Atoms	Z	Observed(^{o})	Ideal(^{o})
2	B	350	CEI	C8-C9-N1	3.85	123.69	118.40
2	B	350	CEI	C10-C2-N1	3.34	121.66	114.77
2	A	350	CEI	C10-C2-N1	3.21	121.40	114.77
2	A	350	CEI	C8-C9-N1	3.20	122.80	118.40
2	B	350	CEI	C11-C10-C2	2.98	121.39	112.57
2	A	350	CEI	C11-C10-C2	2.73	120.65	112.57
2	B	350	CEI	O33-C2-N1	-2.55	118.98	123.63
2	B	350	CEI	C8-C9-N4	-2.52	119.91	122.25
2	A	350	CEI	C19-C6-N7	2.40	119.43	116.02
2	B	350	CEI	C6-N7-C8	2.25	120.31	118.35
2	B	350	CEI	C5-N4-C9	2.06	120.19	116.05

There are no chirality outliers.

All (7) torsion outliers are listed below:

Mol	Chain	Res	Type	Atoms
2	B	350	CEI	C8-C9-N1-C2
2	B	350	CEI	C27-C26-C8-C9
2	B	350	CEI	C27-C26-C8-N7
2	A	350	CEI	C8-C9-N1-C2
2	A	350	CEI	C27-C26-C8-C9
2	A	350	CEI	C27-C26-C8-N7
2	A	350	CEI	C11-C10-C2-N1

There are no ring outliers.

2 monomers are involved in 4 short contacts:

Mol	Chain	Res	Type	Clashes	Symm-Clashes
2	B	350	CEI	3	0

Continued on next page...

PRELIMINARY VALIDATION REPORT

Continued from previous page...

Mol	Chain	Res	Type	Clashes	Symm-Clashes
2	A	350	CEI	1	0

The following is a two-dimensional graphical depiction of Mogul quality analysis of bond lengths, bond angles, torsion angles, and ring geometry for all instances of the Ligand of Interest. In addition, ligands with molecular weight > 250 and outliers as shown on the validation Tables will also be included. For torsion angles, if less then 5% of the Mogul distribution of torsion angles is within 10 degrees of the torsion angle in question, then that torsion angle is considered an outlier. Any bond that is central to one or more torsion angles identified as an outlier by Mogul will be highlighted in the graph. For rings, the root-mean-square deviation (RMSD) between the ring in question and similar rings identified by Mogul is calculated over all ring torsion angles. If the average RMSD is greater than 60 degrees and the minimal RMSD between the ring in question and any Mogul-identified rings is also greater than 60 degrees, then that ring is considered an outlier. The outliers are highlighted in purple. The color gray indicates Mogul did not find sufficient equivalents in the CSD to analyse the geometry.

Ligand CEI B 350

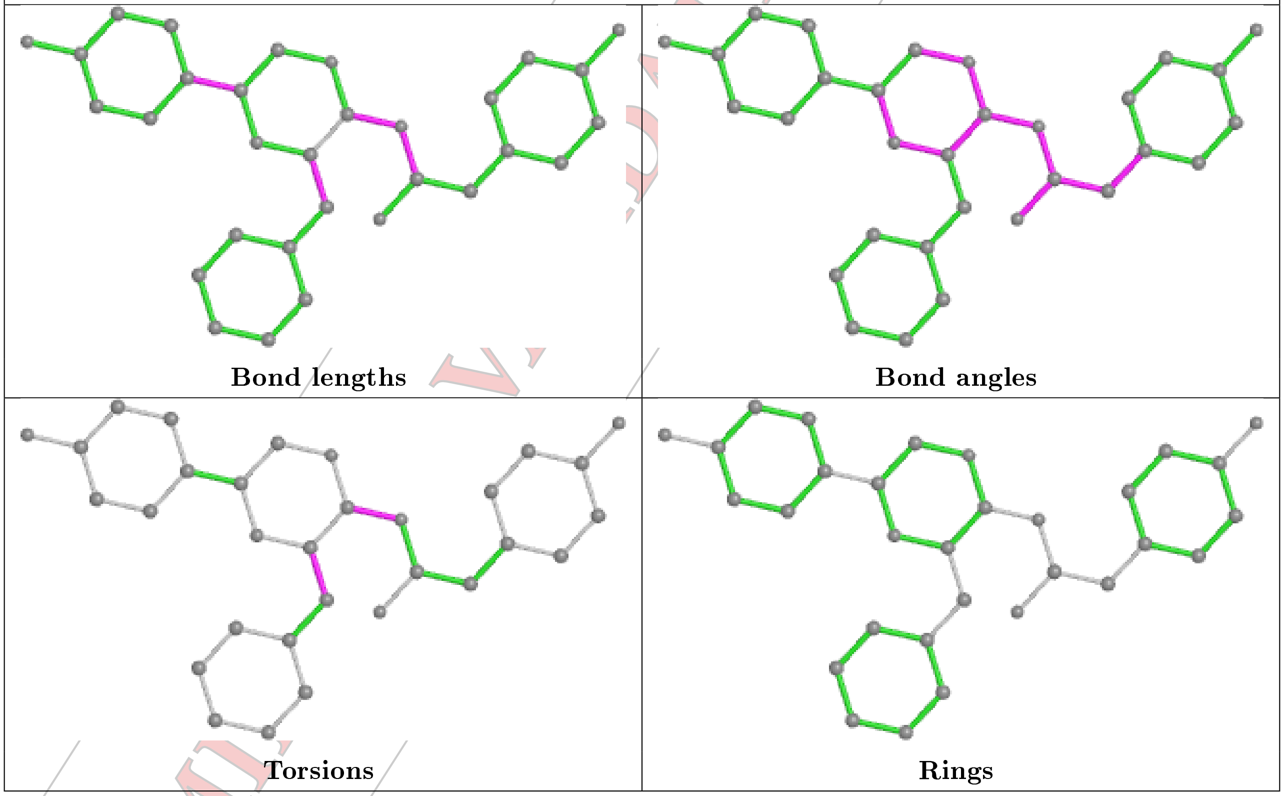

PRELIMINARY VALIDATION REPORT

Ligand CEI A 350

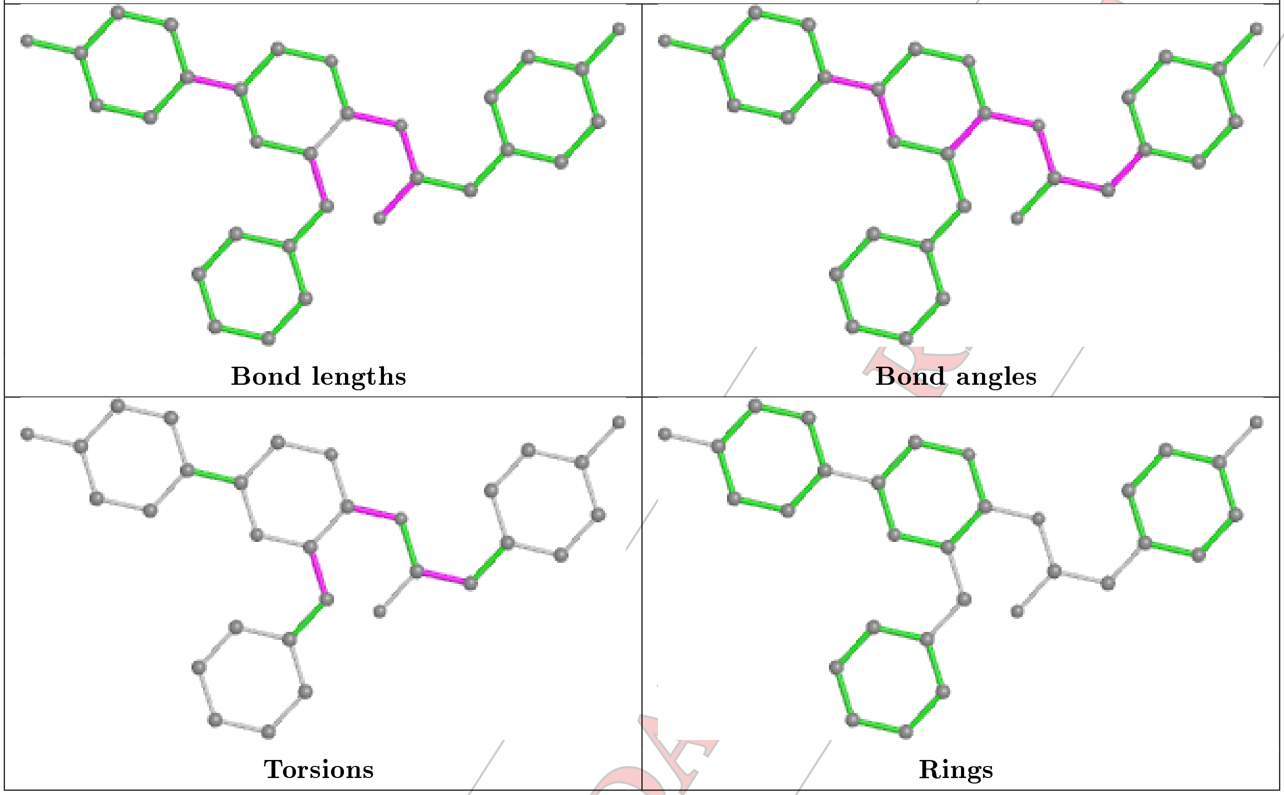

5.7 Other polymers (i)

There are no such residues in this entry.

5.8 Polymer linkage issues (i)

There are no chain breaks in this entry.

PRELIMINARY VALIDATION

6 Fit of model and data (i)

6.1 Protein, DNA and RNA chains (i)

In the following table, the column labelled '#RSRZ> 2' contains the number (and percentage) of RSRZ outliers, followed by percent RSRZ outliers for the chain as percentile scores relative to all X-ray entries and entries of similar resolution. The OWAB column contains the minimum, median, 95th percentile and maximum values of the occupancy-weighted average B-factor per residue. The column labelled 'Q< 0.9' lists the number of (and percentage) of residues with an average occupancy less than 0.9.

Mol	Chain	Analysed	<RSRZ>	#RSRZ>2			OWAB(Å^2)	Q<0.9
1	A	310/317 (97%)	-0.14	11 (3%)	44	47	23, 32, 52, 74	0
1	B	309/317 (97%)	-0.25	5 (1%)	72	75	23, 31, 46, 65	0
All	All	619/634 (97%)	-0.19	16 (2%)	56	59	23, 31, 50, 74	0

All (16) RSRZ outliers are listed below:

Mol	Chain	Res	Type	RSRZ
1	A	154	ASP	4.6
1	A	3	SER	4.0
1	A	12	LYS	3.9
1	B	154	ASP	3.7
1	A	311	GLN	3.6
1	A	153	TRP	3.5
1	A	8	PRO	3.4
1	B	155	GLU	3.4
1	B	12	LYS	3.3
1	A	2	THR	2.7
1	A	155	GLU	2.4
1	A	9	GLU	2.3
1	A	308	LYS	2.2
1	A	310	GLU	2.1
1	B	8	PRO	2.0
1	B	153	TRP	2.0

6.2 Non-standard residues in protein, DNA, RNA chains (i)

There are no non-standard protein/DNA/RNA residues in this entry.

PRELIMINARY VALIDATION REPORT

6.3 Carbohydrates (i)

There are no carbohydrates in this entry.

6.4 Ligands (i)

In the following table, the Atoms column lists the number of modelled atoms in the group and the number defined in the chemical component dictionary. The B-factors column lists the minimum, median, 95th percentile and maximum values of B factors of atoms in the group. The column labelled 'Q< 0.9' lists the number of atoms with occupancy less than 0.9.

Mol	Type	Chain	Res	Atoms	RSCC	RSR	B-factors($Å^2$)	Q<0.9
2	CEI	A	350	31/?	0.91	0.11	31,43,51,62	0
4	ACT	A	500	4/?	0.94	0.17	35,43,46,47	0
2	CEI	B	350	31/?	0.95	0.09	32,40,47,52	0
3	GOL	A	400	6/?	0.97	0.07	27,31,35,35	0
5	K	B	450	1/?	0.97	0.10	35,35,35,35	0

The following is a graphical depiction of the model fit to experimental electron density of all instances of the Ligand of Interest. In addition, ligands with molecular weight > 250 and outliers as shown on the geometry validation Tables will also be included. Each fit is shown from different orientation to approximate a three-dimensional view.

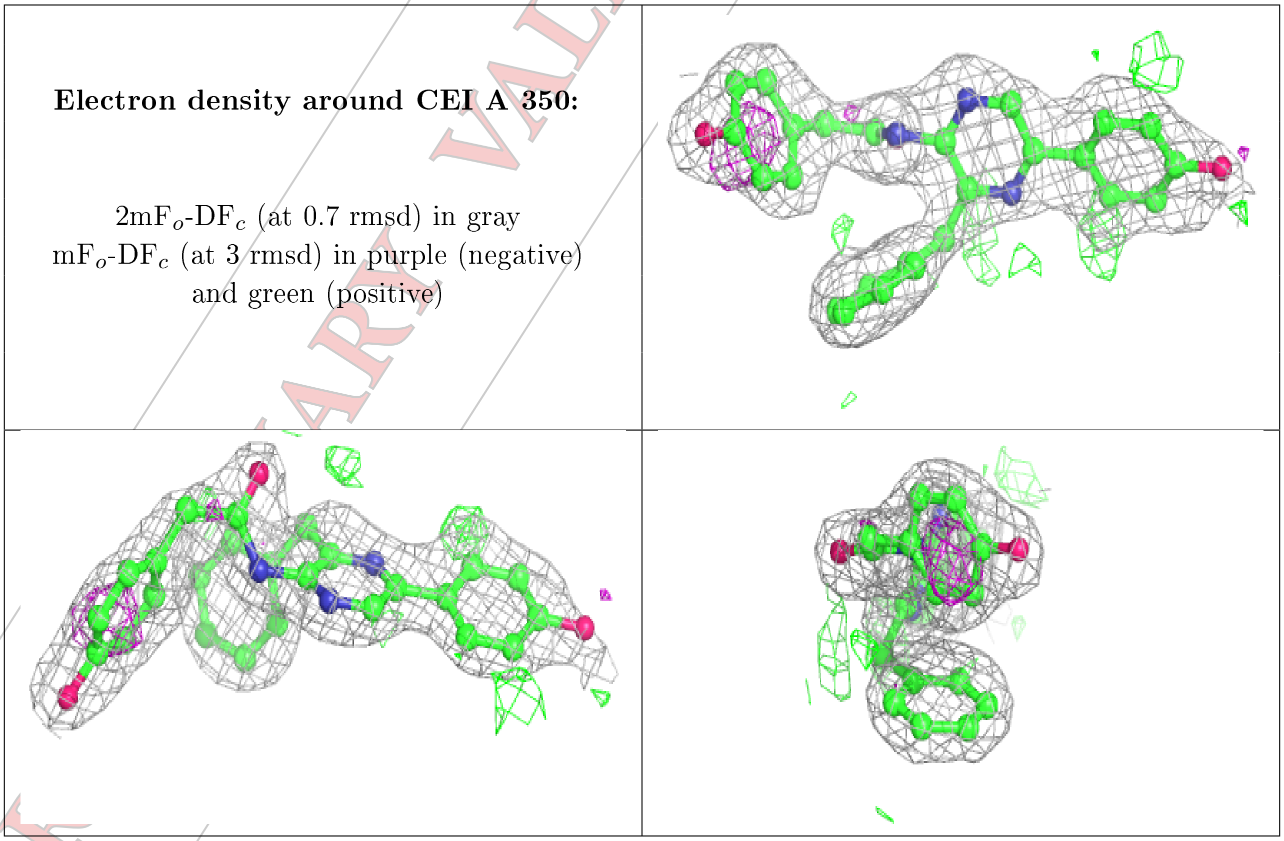

PRELIMINARY VALIDATION REPORT

Electron density around CEI B 350:

2mF$_o$-DF$_c$ (at 0.7 rmsd) in gray
mF$_o$-DF$_c$ (at 3 rmsd) in purple (negative)
and green (positive)

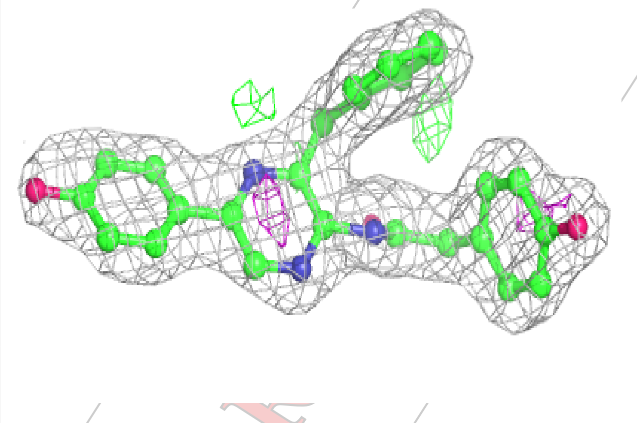

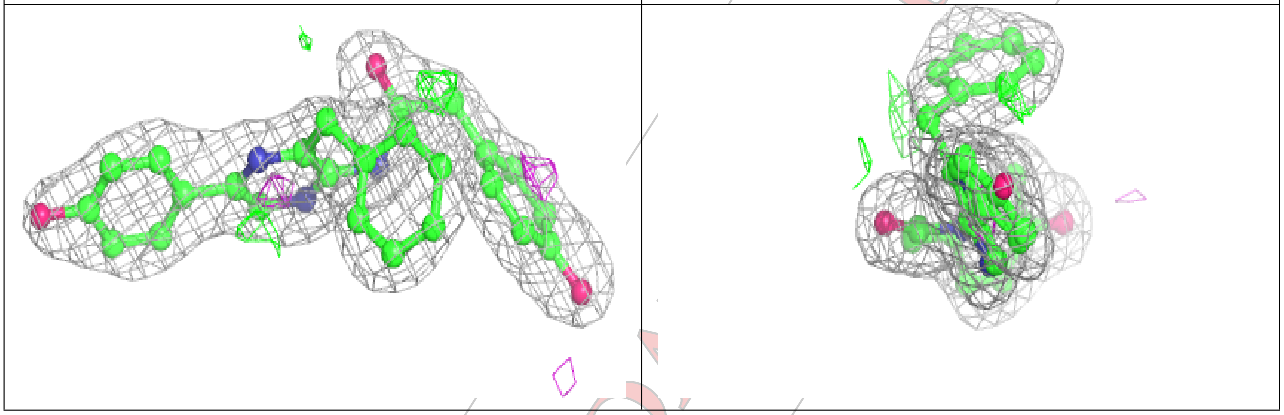

6.5 Other polymers ⓘ

There are no such residues in this entry.

PRELIMINARY VALIDATION

References

1. Emond, S. *et al.* Accessing unexplored regions of sequence space in directed enzyme evolution via insertion/deletion mutagenesis. *Nat. Commun.* **11**, 1–14 (2020).
2. Green, B., Bouchier, C., Fairhead, C., Craig, N. L. & Cormack, B. P. Insertion site preference of Mu, Tn5, and Tn7 transposons. *Mob. DNA* **3**, 3 (2012).
3. Doruker, P., Atilgan, A. R. & Bahar, I. Dynamics of proteins predicted by molecular dynamics simulations and analytical approaches: Application to α-amylase inhibitor. *Proteins Struct. Funct. Bioinforma.* **40**, 512–524 (2000).
4. Eyal, E., Yang, L.-W. & Bahar, I. Anisotropic network model: systematic evaluation and a new web interface. *Bioinformatics* **22**, 2619–2627 (2006).
5. Tirion, M. M. Large Amplitude Elastic Motions in Proteins from a Single-Parameter, Atomic Analysis. *Phys. Rev. Lett.* **77**, 1905–1908 (1996).
6. Rojewska, D. & Elber, R. Molecular dynamics study of secondary structure motion in proteins: Application to myohemerythrin. *Proteins Struct. Funct. Bioinforma.* **7**, 265–279 (1990).
7. Loening, A. M., Fenn, T. D. & Gambhir, S. S. Crystal structures of the luciferase and green fluorescent protein from Renilla reniformis. *J. Mol. Biol.* **374**, 1017–1028 (2007).
8. Chaloupkova, R. *et al.* Light-emitting dehalogenases: Reconstruction of multifunctional biocatalysts. *ACS Catal.* **9**, 4810–4823 (2019).
9. Wold, S. & Dunn, W. J. Multivariate quantitative structure-activity relationships (QSAR): conditions for their applicability. *J. Chem. Inf. Comput. Sci.* **23**, 6–13 (1983).
10. Wold, S. Validation of QSAR's. *Quant. Struct.-Act. Relatsh.* **10**, 191–193 (1991).
11. *3D QSAR in Drug Design: Volume 1: Theory Methods and Applications*. (Springer Netherlands, 1994).
12. Loening, A. M., Fenn, T. D., Wu, A. M. & Gambhir, S. S. Consensus guided mutagenesis of Renilla luciferase yields enhanced stability and light output. *Protein Eng. Des. Sel.* **19**, 391–400 (2006).
13. Johnson, K. A. Chapter 23 Fitting Enzyme Kinetic Data with KinTek Global Kinetic Explorer. in *Methods in Enzymology* (eds. Johnson, M. L. & Brand, L.) vol. 467 601–626 (Academic Press, 2009).
14. Johnson, K. A., Simpson, Z. B. & Blom, T. Global Kinetic Explorer: A new computer program for dynamic simulation and fitting of kinetic data. *Anal. Biochem.* **387**, 20–29 (2009).

15. Johnson, K. A., Simpson, Z. B. & Blom, T. FitSpace Explorer: An algorithm to evaluate multidimensional parameter space in fitting kinetic data. *Anal. Biochem.* **387**, 30–41 (2009).
